# Proteome and Physiological Characterization of Halotolerant Nodule Endophytes: The Case of *Rahnella aquatilis* and *Serratia plymuthica*

**DOI:** 10.3390/microorganisms10050890

**Published:** 2022-04-24

**Authors:** Giorgia Novello, Elisa Gamalero, Nadia Massa, Patrizia Cesaro, Guido Lingua, Valeria Todeschini, Alice Caramaschi, Francesco Favero, Davide Corà, Marcello Manfredi, Emilio Marengo, Micaela Pelagi, Loredana Pangaro, Giuseppina Caffiero, Fulvia Milano, Elisa Bona

**Affiliations:** 1 Dipartimento di Scienze e Innovazione Tecnologica, Università del Piemonte Orientale, 15121 Alessandria, Italy; elisa.gamalero@uniupo.it (E.G.); nadia.massa@uniupo.it (N.M.); patrizia.cesaro@uniupo.it (P.C.); guido.lingua@uniupo.it (G.L.); valeria.todeschini@uniupo.it (V.T.); emilio.marengo@uniupo.it (E.M.); 2 Dipartimento per lo Sviluppo Sostenibile e la Transizione Ecologica, University of Piemonte Orientale, 13100 Vercelli, Italy; alice.caramaschi@uniupo.it; 3 Center on Autoimmune and Allergic Diseases (CAAD), Università del Piemonte Orientale, 28100 Novara, Italy; francesco.favero@uniupo.it (F.F.); davide.cora@uniupo.it (D.C.); marcello.manfredi@uniupo.it (M.M.); 4 Dipartimento di Medicina Traslazionale, Università del Piemonte Orientale, 28100 Novara, Italy; 5 Laboratorio Analisi e Microbiologia, ASL-VC Ospedale Sant’Andrea, 13100 Vercelli, Italy; micaela.pelagi@aslvc.piemonte.it (M.P.); loredana.pangaro@aslvc.piemonte.it (L.P.); giuseppina.caffiero@aslvc.piemonte.it (G.C.); fulvia.milano@aslvc.piemonte.it (F.M.)

**Keywords:** bacterial endophytes, legume nodules, salt tolerance, plant beneficial activities, proteome

## Abstract

Bacterial endophytes were isolated from nodules of pea and fava bean. The strains were identified and characterized for plant beneficial activities (phosphate solubilization, synthesis of indole acetic acid and siderophores) and salt tolerance. Based on these data, four strains of *Rahnella aquatilis* and three strains of *Serratia plymuthica* were selected. To shed light on the mechanisms underlying salt tolerance, the proteome of the two most performant strains (Ra4 and Sp2) grown in the presence or not of salt was characterized. The number of proteins expressed by the endophytes was higher in the presence of salt. The modulated proteome consisted of 302 (100 up-regulated, 202 down-regulated) and 323 (206 up-regulated, 117 down-regulated) proteins in Ra4 and Sp2, respectively. Overall, proteins involved in abiotic stress responses were up-regulated, while those involved in metabolism and flagellum structure were down-regulated. The main up-regulated proteins in Sp2 were thiol: disulfide interchange protein DsbA, required for the sulfur binding formation in periplasmic proteins, while in Ra4 corresponded to the soluble fraction of ABC transporters, having a role in compatible solute uptake. Our results demonstrated a conserved response to salt stress in the two taxonomically related species.

## 1. Introduction

Until the early 2000s, rhizobia were considered the sole bacteria able to colonize legume root nodules. This idea quickly changed when several works highlighted the occurrence of other bacterial strains inside the root nodules [1,2]. These microorganisms, living inside nodules but unable to form them, are collectively called Non-Rhizobia Endophytic (NRE) bacteria and have been classified as opportunistic colonizers [3]. It has been proposed that NRE bacteria may enter the nodule through infection threads, whose formation is induced in legume roots by rhizobia [3,4]. Therefore, rhizobia and other bacterial species frequently coexist in nodules [5,6].

NRE bacteria mainly belong to alpha- (*Agrobacterium*, *Methylobacterium*, *Ochrobactrum*, *Microvirga*, *Phyllobacterium*, *Devosia*, *Shinella*), beta- (*Herbaspirillum*), and gamma- (*Acinetobacter*, *Enterobacter*, *Pantoea*, *Pseudomonas*, *Stenotrophomonas*) Proteobacteria, Firmicutes (*Bacillus, Paenibacillus, Staphylococcus*), and Actinobacteria (*Micromonospora, Microbacterium, Arthrobacter, Mycobacterium*) [1]. The biodiversity of the culturable bacteria isolated from nodules of spontaneous *Sulla coronaria* has been studied by Amplified Ribosomal DNA Restriction Analysis (ARDRA) and 16S rDNA sequencing techniques. The results obtained highlighted that *Rhizobium sullae*, the typical rhizobium associated with field cropped varieties, occurred only in 25 out of 60 nodules, while the remaining 35 were colonized by unculturable strains of *Pseudomonas* sp., *Microbacterium* sp., and *Pantoea agglomerans* [7]. More recently, it has been reported that only 10% of the bacterial strains isolated from nodules of wild *Lotus parviflorus* plants are able to nodulate legumes (*Bradyrhizobium canariense* and *Bradyrhizobium* sp.), while 90% are represented by opportunistic nodule associated bacteria classified in alpha- (*Rhizobium/Agrobacterium*), beta- (*Massilia*), and gamma- (*Pseudomonas, Lysobacter, Luteibacter, Stenotrophomonas,* and *Rahnella*) Proteobacteria [8]. A study performed on cowpea cultivated in two semi-arid regions of Brazil with different soil characteristics (a silty-clay Haplic Vertisol and a sandy-loam Red Yellow Dystrophic Ultisol) demonstrated that the soil type is the main factor affecting the biodiversity of the nodule microbiome [9], with *Chryseobacterium* spp. as the dominant genus inhabiting cowpea nodules. Since *Chryseobacterium* strains were previously reported to enhance the tolerance of *Phaseolus vulgaris* to moderate saline stress [10], it has been hypothesized that NRE bacterial strains belonging to this genus may support cowpea plant growth in the presence of saline stress, a condition that frequently occurs in semi-arid regions.

While the role of nodule endophytes is unclear, it is widely accepted that they usually do not induce negative effects on plant growth. Contrarily, since their discovery, some papers have underlined a direct beneficial activity on legume plants, such as an increase in the nodulation level and improvement of nitrogen fixation efficiency [11,12,13], as well as indirect biocontrol activity against phytopathogenic fungi and nematodes [14,15]. Several strains of NRE can synthesize auxins and siderophores, solubilize phosphate, and fix nitrogen and some of them showed the capability to increase plant tolerance to stress [16,17,18,19].

Plants belonging to the Fabaceae family are usually classified as sensitive or moderately sensitive to abiotic stress, especially salinity [20]. In fact, salt stress not only affects different early phases of the symbiosis establishment with rhizobia, nodule formation, and function (i.e., nitrogen fixation) but also plant growth and root development [21]. However, rhizobia and NRE bacteria seem to be more tolerant to salt stress than the host legume [22,23]. Overall, our knowledge of NRE bacteria is still scanty: less than 100 papers have been published on bacteria living in this peculiar habitat. Information on the mechanisms by which NRE bacteria tolerate mild salt stress and possibly contribute to reducing the intensity of the stressful condition for the symbiosis is completely lacking.

This work has two main purposes: (i) to isolate, identify and characterize salt stress-tolerant nitrogen-fixing bacteria, and (ii) to shed light on the molecular mechanisms at the basis of salt stress tolerance using a proteomic approach.

## 2. Materials and Methods

### 2.1. Isolation of Bacterial Strains from Vicia fava and Pisum sativum Nodules

**Figure 1:**
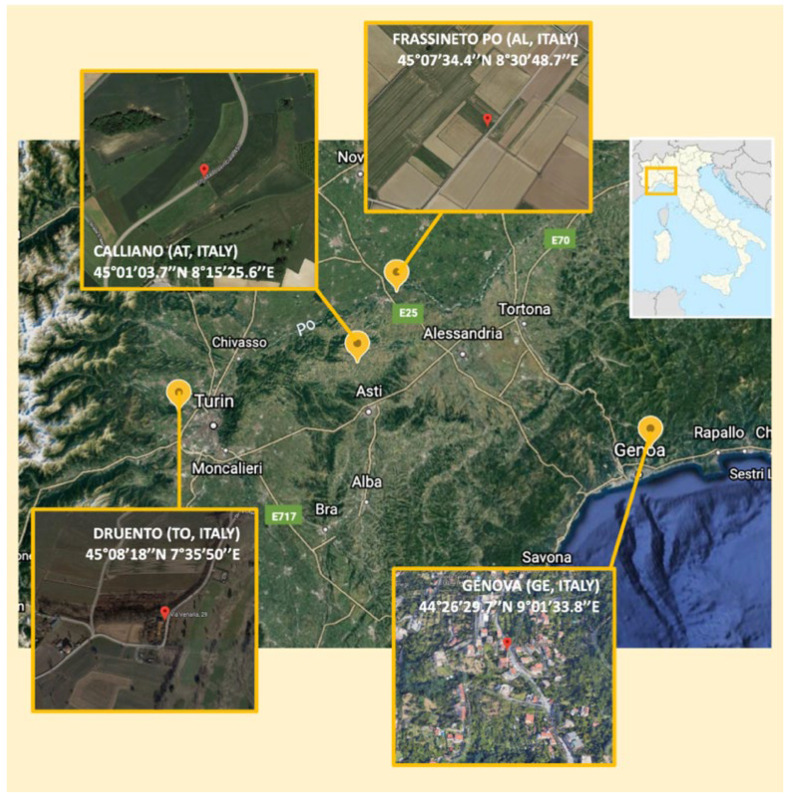
View of the sampling map. The image was produced using Google Earth’s online version.

The isolation of the endophytic bacterial strains was performed from nodules of fava bean (*Vicia fava* L.) and pea (*Pisum sativum* L.) cultivated in different geographical areas of northern Italy. In particular, three fava bean plants in each site were sampled: in Genoa (44°26′29.7″ N, 9°01′33.8″ E), in Calliano (Asti, Italy) (45°01′03.7″ N, 8° 15′25.6″ E), and in Frassineto Po (Alessandria, Italy) (45°07′34.4″ N, 8° 30′48.7″ E), while three pea plants were sampled in Calliano (Asti, Italy) (45°01′03.7″ N, 8° 15′25.6″ E) and Druento (TO) (45°08′18″ N, 7°35′50″ E), respectively (Figure 1).

Roots of pea and fava bean (3 plants in each considered site) were harvested and washed with tap water. Nodules were separated from roots with a sterile scalpel and placed in a 50 mL sterile tube to which 30 mL of sterile deionized water was added for 15 min, under slow shaking. This procedure was repeated 4 times to reduce bacterial density on the nodule surface. Then, the nodules were surface sterilized in 1% sodium hypochlorite for 5 min. Next, 5 more washing steps (1 min each) were performed in sterile physiological solution (NaCl 0.9%). To check the nodule surface sterilization efficacy, 5 nodules per sample were incubated on Trypticase Soy Agar (VWR, Milan, Italy) for 5 days at 28 °C. Nodules were then homogenized in 10 mL of MgSO_4_, 0.1 M, with a Tissue Lyser at 560 rpm for 5 min. Finally, 100 µL of the suspension was spread, in triplicate, onto Yeast Mannitol Agar (YMA) plates added with cycloheximide (100 µg mL^−1^) (Sigma Aldrich, Milan, Italy) [24]. This growth medium is widely accepted as selective for nitrogen-fixing bacteria.

### 2.2. Identification of Bacterial Endophytes

Pure colonies grown on YMA were described for their morphology and subjected to Gram staining.

Identification of the bacterial strains was performed both by mass spectrometry MALDI (Matrix-Assisted Laser Desorption/Ionization) and TOF/TOF (UltrafleXtreme, Bruker) equipped with Biotyper identification software (version 2.0; Bruker Daltonics) and by 16S rDNA sequencing.

A pure bacterial colony was spotted on an MTP 384 target plate polished steel (Bruker Daltonics, Milan, Italy). The spot was firstly overlaid with 70% Formic acid (Sigma Aldrich, Milan, Italy), after drying with 1 μL HCCA (alpha-cyano-4-hydroxycinnamic acid) (Bruker, Milan, Italy) in 50% acetonitrile–2.5% trifluoroacetic acid in water as matrix solution. The preparation was dried at room temperature until co-crystallization with the sample. The resulting mass spectra were analyzed by the BioTyper software and referred to standard matching. The obtained results were further validated by 16S rDNA sequencing.

Nucleo Spin tissue DNA purification kit (Macherey-Nagel, M-Medical, Cornaredo, Milan) was employed to extract genomic DNA of the bacterial endophytes, following the manufacturer’s instructions. PCR amplification of 16S rDNA was performed using the fD1/RP2 primer pair, whose sequence has been reported in Weisburg et al. [25]. The PCR reaction was carried out as described in Gamalero et al. [26]. Specifically, initial denaturation at 94 °C for 5 min; 34 cycles at 94 °C for 1 min, 60 °C for 1 min, and 72 °C for 1 min 30 s, with 72 °C for 10 min as the final extension time. The resulting amplified product was purified with Nucleo Spin Extract II kit (Macherey-Nagel) and sequenced by BMR Genomics (Padua, Italy). Electropherograms were assembled using Sequencing Analysis Software version 5.1 (Applied Biosystems, Milan, Italy) and transformed into a text file for further bioinformatic analyses.

DNA sequences were compared against all bacterial 16S rDNA reference sequences available at the NCBI World Wide Web database using BLAST. The identified sequences were loaded in GenBank.

### 2.3. Physiological Characterization of Plant Beneficial Traits

All the nodule endophytic bacterial strains were characterized for the physiological activities at the base of the plant growth promotion effect.

The capability to synthesize siderophores was assessed on universal Chrome Azurol S (CAS) agar [27]. A colony of each bacterial strain was inoculated at the center of the CAS medium. Plates were incubated at 28 °C for 5 days and monitored daily for the appearance of an orange halo around the colony, indicating siderophore synthesis. The measurements were done in triplicate, and siderophore production was expressed as a ratio between the two diameters of the halo and the two diameters of the colony.

Phosphate solubilization capability was assessed on 2 media containing dicalcium phosphate (DCP) (Sigma Aldrich, Milan, Italy) or tricalcium phosphate (TCP) (Sigma Aldrich, Milan, Italy). A colony of each strain was spotted at the center of the plate and incubated at 28 °C for 15 days. Solubilization of DCP and TCP was indicated by the formation of a clarification halo under the colony and by colony growth, respectively [24].

The synthesis of indole-3-acetic acid (IAA) was performed according to De Brito Alvarez et al. [28]. Briefly, a single colony of each bacterial strain was inoculated onto a nitrocellulose disk placed on 10% TSA (VWR, Milan, Italy) added with 5 mM L-tryptophan (Sigma Aldrich, Milan, Italy). After 3 days of incubation at 28 °C, nitrocellulose membranes were dipped in 20 mL of Salkowsky’s reagent (composed of 2% FeCl_3_ in 35% perchloric acid) (Sigma Aldrich, Milan, Italy). The possible development of a red/pink halo around the colony spot indicated the capability to produce IAA.

### 2.4. Tolerance to Temperature and Antibiotic Resistance Profile

In order to assess temperature tolerance, bacterial colonies were transplanted on TSA and Mueller Hinton Agar (MH) (VWR, Milan, Italy) plates and incubated for 2 days at 4, 28, 37, or 42 °C.

The antibiotic resistance profile was characterized through both agar disk diffusion and Minimal Inhibitory Concentration (MIC) assays according to the EUCAST method with some modifications.

Bacterial suspensions containing 10^8^ colony-forming units/mL (cfu/mL) (optical density 0.5 at 600 nm of wavelength) prepared in MgSO_4_, 0.1 M, were swabbed on MH medium. Disks containing erythromycin 15 µg, chloramphenicol 30 µg, rifampicin 30 µg, streptomycin 10 µg, neomycin 30 µg, kanamycin 30 µg, Cefepime (30 µg), Cefotaxime 5 µg, Ceftazidime (10 µg), Ciprofloxacin (5 µg), Gentamicin 10 µg, Trimethoprim-Sulphamethazole (1.25 + 23.75 µg), Fosfomycin (200 µg), Amoxicillin (3 µg), Meropenem (10 µg), Piperacillin/Tazobactam (36 µg) were deposited on the agar surface. Then, the plates were incubated at 28 °C for 24 h, considering their optimal growth temperature. The diameter of the growth inhibition halo was measured with a caliper (mm). The results were interpreted according to Stock et al. [29], Massa et al. [24], and EUCAST Clinical Breakpoint Tables (v. 12.0) for Enterobacterales.

The MICs of the 15 antibacterial drugs Amikacin, Amoxicillin/Clavulanic Acid, Cefepime, Cefotaxime, Ceftazidime, Ciprofloxacin, Ertapenem, Gentamicin, Mero-penem, Piperacillin/Tazobactam, Trimethoprim-Sulphamethazole, Ampicillin, Aztre-onam, Colistin, Levofloxacin, Piperacillin, Ticarcillin, Tigecycline, and Tobramycin were measured by VITEK^®^ 2 AST card using the VITEK^®^ 2 automated system (BioMerieux, Lyon, France). Briefly, strain suspensions obtained in physiological solution were adjusted to 0.5 McFarland by measuring absorbance at 600 nm. These suspensions were then loaded into the instrument in VITEK^®^ 2 AST cards that provided a series of antibiograms and tests to detect resistance.

### 2.5. Salt Tolerance and Growth Curve

The salt tolerance of each bacterial strain was evaluated through the determination of the Minimum Inhibitory Concentration (MIC) using a microdilution method in a 96-well microtiter plate, according to the EUCAST method modified by adding NaCl into the medium. A 24 h culture of each bacterial strain was diluted in MgSO_4_ (0.1 M) to reach 10^8^ cfu/mL (optical density 0.5 at 600 nm wavelength). First, sodium chloride (Fluka, Milan, Italy) was dissolved in tryptic soy broth (TSB) (VWR, Milan, Italy) with a concentration ranging from 14.4 to 0.014%. In a second step, to better understand the true range of bacterial tolerance, NaCl was diluted from 7.2 to 2.4%. A negative control, containing TSB with each NaCl dilution, as well as a positive control represented by TSB inoculated with the bacterial strains at the same NaCl concentrations, were set up. All plates were incubated at 28 °C for 48 h. A well was considered positive when the formation of a visible bacterial cell pellet occurred. Each experiment was performed in triplicate. Salt tolerance assay results were used to select the best performing strains to investigate the salt response by proteomic analysis.

### 2.6. 1-Aminocyclopropane-1-carboxylic Acid (ACC) Deaminase Assay

Two strains (*Rahnella aquatilis* Ra4 and *Serratia plymuthica* Sp2) were selected according to the results obtained by salt tolerance assay and were tested for their 1-aminocyclopropane-1-carboxylic acid (ACC) deaminase capability using the method described by Penrose and Glick [30]. Briefly, bacterial cells were grown for 24 h at 28 °C in tryptic soy broth (TSB medium) and then in Dworkin and Foster (DF) [31] salts minimal medium added of 45 µL 0.5 M ACC (Sigma Aldrich, Milan, Italy) solution. The bacteria were first washed with 0.1 M Tris-HCl, pH 7.6, suspended in 0.1 M Tris-HCl, pH 8.5, and then in toluene (Sigma Aldrich, Milan, Italy). An aliquot of the cells treated with toluene was stored for protein assay. The remaining aliquot was incubated at 30 °C for 15 min in the presence or the absence of ACC. A total of 1 milliliter of 0.56 M HCl was added to the suspension and centrifuged at 16,000× *g* at room temperature for 10 min. Then, 1 milliliter of the supernatant was vortexed together with 800 µL of 0.56 M HCl and 300 µL of the 2,4-dinitrophenylhydrazine reagent. After 30 min of incubation at 30 °C, 2 mL 2 N NaOH was added, and the absorbance of the mixture was measured at 540 nm. *Pseudomonas migulae* 8R6 [32], able to synthesize ACC deaminase and to produce alpha-ketobutyrate and its mutant, unable to synthesize this enzyme, were used as positive and negative controls, respectively.

### 2.7. Proteome Analysis

Salt response was investigated in Ra4 and Sp2 strains comparing the proteome produced by treated cells (4.4% NaCl) versus untreated cells. The experiment was conducted in triplicate in TSB. In each strain, both the control (TSB) and 4.4% NaCl (treated) solution were inoculated with an optical density of 10^8^ cfu/mL diluted 1:1000, reaching a final bacterial concentration of 10^5^ cfu/mL. The cultures were incubated at 28 °C under shaking for 24 h. At the end of incubation, the samples were centrifuged for 15 min RT at 1500× *g*. To recover proteins, 5 volumes of 0.1 M ammonium acetate in methanol were added to 10 mL of supernatant and incubated overnight at −20 °C.

The precipitated proteins were recovered by centrifugation for 50 min at 4 °C, 1500× *g*. The resulting protein pellet was resuspended in 400 μL of 50 mM ammonium bicarbonate and quantified by the Bradford method [33] using Bovine Serum Albumin (Sigma Aldrich, Milan, Italy) as standard (from 2 to 0.031 µg µL^−1^). Proteins were Trypsin digested as fully reported in Bona et al. [34,35].

The mass spectrometry analyses were performed using a micro-LC Eksigent Technologies (Dublin, OH, USA) system with a Halo Fused C18 column (0.5 × 100 mm, 2.7 μm) as a stationary phase. The injection volume was 4.0 μL, and the oven temperature was set at 40 °C. The mobile phase was a mixture of 0.1% (*v/v*) formic acid in water (A) and 0.1% (*v/v*) formic acid in acetonitrile (B), eluting at a flow rate of 15.0 μL min^−1^ at an increasing concentration of solvent B from 2% to 40% in 30 min. The LC system was interfaced with a 5600+ TripleTOF system (AB Sciex, Concord, ON, Canada) equipped with a DuoSpray Ion Source. The samples were subjected to the traditional data-dependent acquisition (DDA): mass spectrometer analysis was performed using a mass range of 100–1500 Da, followed by an MS/MS product ion scan from 200 to 1250 Da with the abundance threshold set at 30 counts per second (cps) (35 candidate ions can be monitored during every cycle).

For label-free quantification, samples were then subjected to cyclic data independent analysis (DIA) of the mass spectra using a 25-Da window. A 50-ms survey scan (TOF-MS) was performed, followed by MS/MS experiments on all precursors. These MS/MS experiments were performed in a cyclic manner using an accumulation time of 40 ms per 25-Da swath (36 swaths in total) for a total cycle time of 1.5408 s.

The ions were fragmented for each MS/MS experiment in the collision cell using the rolling collision energy. The MS data were acquired with Analyst TF 1.7 (AB Sciex) [36]. Mass data were analyzed using Mascot (Matrix Science Inc., Boston, MA, USA) against the NCBI databases of *Rahnella aquatilis* (71,017 sequences, 16 September 2019) and *Serratia plymuthica* (130,272 sequences, 16 September 2019).

The search was performed on Mascot v. 2.3.0; the digestion enzyme selected was Trypsin, with 3 maximum missed cleavages; a search tolerance of 120 ppm was specified for the peptide mass tolerance, and 0.6 Da for the MS/MS tolerance. The charges of the peptides to search for were set to 2+, 3+, and 4+, and the search was set on monoisotopic mass. The following modifications were used: oxidized methionine and deamidation (NQ) as variable modifications. Proteins with at least 1 peptide with an ion score higher than the homology or identity ion score value were considered significant.

Moreover, the quantification was performed by integrating the extracted ion chromatogram of all the unique ions for a given peptide. The quantification was carried out with PeakView 2.2 and MarkerView 1.2. (Sciex, Concord, ON, Canada). A total of 6 peptides per protein and 6 transitions per peptide were extracted from the SWATH files. Shared peptides were excluded as well as peptides with modifications. Peptides with FDR lower than 1.0% were exported in MarkerView for the *t*-test [37].

### 2.8. Bioinformatic Analysis

All identified proteins were annotated with eggNOG-mapper software (v. 2.0.1) (Cantalapiedra CP), and both KEGG pathway terms and GO Terms were associated with each protein.

Differentially expressed proteins were considered for both *R. aquatilis* Ra4 and *S. plymutica* Sp2 using a |log2FC| > 1 and *p*-value < 0.05 as cutoffs for differential expression. T-tests between Ra4 treated with salt (4.4% NaCl) against the corresponding control and Sp2 treated with salt (4.4% NaCl) against the corresponding control were performed.

For differential expressed proteins, functional enrichment for KEGG Pathways and GO Terms associated with each protein were tested by a hypergeometric test using the R “phyper” function (*p*-value < 0.05, Fisher’s test). Volcano plots were generated using the “plot” function in R (R core 2020).

### 2.9. Statistical Analysis

Shapiro–Wilk and Levene tests were employed to assess data normality and homogeneity of variance, respectively. Based on the results obtained, parametric (ANOVA and Welch one-way ANOVA) or non-parametric (Kruskal–Wallis and per-mutation test) one-way tests were used to compare controls and 4.4% NaCl in the different bacterial strains. Differences were considered significant for *p* < 0.05. All statistical analyses were performed using R (v. 3.5.1) [R Core Team (2018)].

## 3. Results and Discussion

### 3.1. Isolation, Identification, and Physiological Characterization of Bacterial Strains

A total of 14 and 8 strains were isolated from the nodules cut from the roots of fava bean and pea plants, respectively (Table 1), and sampled as reported in paragraph 2.1 (Figure 1).

Of these, 9 strains developed small pink mucoid colonies, round shape with convex elevation and entire margins, while 7 strains showed large mucoid and pink colonies with entire margins, and 7 strains showed orange-red colonies (Table 1).

These features are typical of rhizobia strains on YMA. All 22 strains were Gram-negative. None of the isolates were able to solubilize TCP, while 18 strains showed solubilization activity on DCP (Table 1). Only the PSEB1A1 and PSEN2B1 strains synthesized siderophores on CAS agar, while 9 strains produced IAA (Table 1).

Based on the characteristics of the colony and the results of the physiological activities, 7 strains (highlighted in green in Table 1) were selected for further analyses.

The nearest neighbors search against all bacterial 16S rDNA reference sequences, available at the NCBI World Wide Web database, allowed the identification of Ra1-4 as *R. aquatilis* and Sp1-3 as *S. plymuthica*. Bacterial 16S rDNA reference sequences of the isolated bacteria are available at the NCBI World Wide Web database GenBank with the accession numbers reported in Table 2. All the isolates were able to grow at 4 °C and 28 °C, showing swarming colonies. At 37 °C, they showed a reduced growth rate and motility loss, while none of the strains grew at 42 °C (Table 2). The swarming capability at 28 °C, but not at 37 °C, suggests that 28 °C is their optimal growth temperature. Consequently, they do not have a pathogenic potential toward animals and humans. Although strains belonging to *R. aquatilis,* and less frequently, *S. plymuthica,* are reported to cause human infections, bacteriemia and sepsis [39,40,41], these bacterial species are considered ubiquitous in the environment and more spread in soil, water and plants. Moreover, these strains could be considered moderate halophilic bacteria as they can grow in the presence of NaCl, ranging from 5.8 to 6.4% of NaCl (Table 2). In literature, it has been reported that *Pantoea ananatis* tolerate 8.15% of NaCl concentration, and *R. aquatilis* survives in up to 6.3% NaCl [42].

Table 3, Table 4 and Table 5 report the results of the response to antibiotics (both disc diffusion and MIC assays), demonstrated by different isolated strains. In particular, the isolates showed an intrinsic resistance only to erythromycin and cefepime, while they were differently sensitive to the other tested antibiotics. These results partly agree with the literature reporting strains of *R. aquatilis* as naturally resistant to many antibiotics, including amoxicillin, ampicillin, rifampicin, fosfomycin, cefotaxime, cephalothin, piperacillin, streptomycin, sulfamethoxazole, ticarcillin, and trimethoprim [29,43,44]. The substantial sensitivity profile of the isolated strains confirms their non-pathogenic nature and therefore makes it possible to use them to relieve salt stress in crop plants.

**Figure 2:**
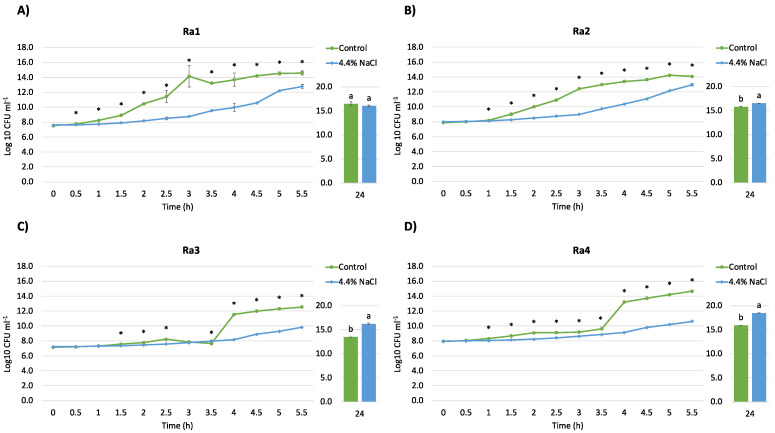
Growth curves of *R. aquatilis* strains. Effect induced by the treatment with 4.4% NaCl (light blue) compared to untreated cells (green) in the first 5.5 h and at 24 h of incubation (28 °C in shaking) on the growth (Log10 cfu/mL) of the different bacterial strains: (**A**) *R. aquatilis* Ra1; (**B**) *R. aquatilis* Ra2; (**C**) *R. aquatilis* Ra3; (**D**) *R. aquatilis* Ra4. Asterisks and different letters indicate statistically significant differences between controls and salt-treated bacteria (*p*-value < 0.05).

Growth curves of selected *R. aquatilis* strains treated or not with 4.4% NaCl are shown in Figure 2. This salt amount was identified (considering the MIC) as a sub-lethal concentration allowing a sufficient bacterial biomass production. The growth of *R. aquatilis* strains was generally inhibited by salt during the first 5.5 h of treatment. However, at 24 h, the growth of Ra 2, 3, and 4 was significantly higher in the presence of 4.4% NaCl than in TSB. This result suggests an adaptation of the strains to the stress probably mediated by the synthesis of compatible solutes such as proline, trehalose, glycine betaine, and other molecules regulating the osmotic responses. This hypothesis is supported by the proteome analysis highlighting the up-regulation of glycine betaine/L-proline ABC transporter substrate-binding protein ProX.

**Figure 3:**
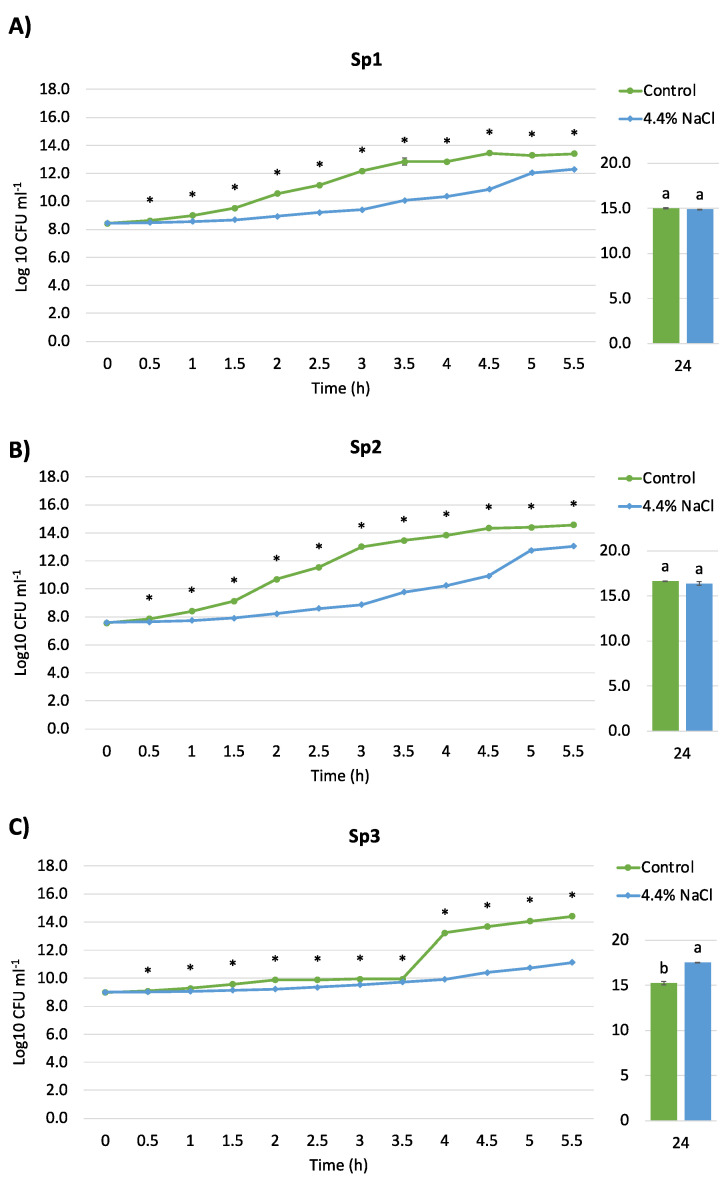
Growth curves of *Serratia plymuthica* strains. Effect induced by the treatment with 4.4% NaCl (light blue) compared to untreated cells (green) in the first 5.5 h and at 24 h of incubation (28 °C in shaking) on the growth (Log10 cfu/mL) of the different bacterial strains: (**A**) *Serratia plymuthica* Sp1; (**B**) *S. plymuthica* Sp2; (**C**) *S. plymuthica* Sp3. Asterisks and different letters indicate statistically significant differences between controls and salt-treated bacteria (*p*-value < 0.05).

All *S. plymuthica* strains were sensitive to salt in the first 5.5 h of treatment (Figure 3). The growth of the strains Sp1 and Sp2 was unaffected by salt stress (Figure 3A,B). Like what was recorded for *R. aquatilis* strains, at 24 h, the density of *S. plymuthica* Sp3 in the presence of salt was higher than that in TSB (Figure 3C)**.** Considering the growth kinetics and the degree of salt tolerance, *R. aquatilis* Ra4 and *S. plymuthica* Sp2 were chosen for further analyses and the evaluation of the proteome response to salt stress.

### 3.2. Proteome Response to Salt Stress

**Figure 4:**
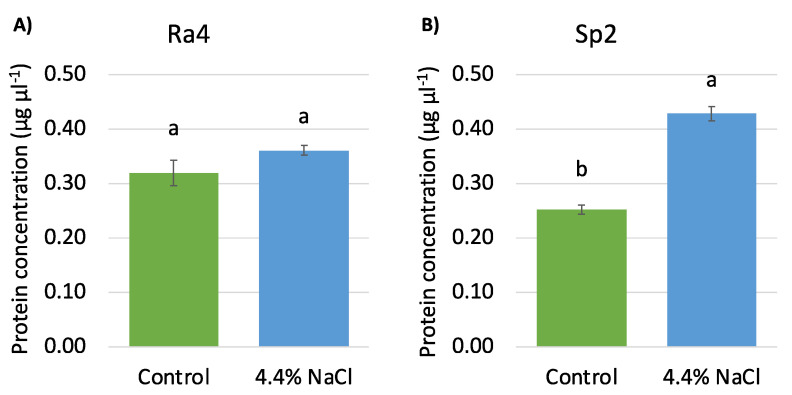
Quantification of proteins (concentration µg µL^−1^) (precipitated from the growth supernatant); Control: bacteria growth for 24 h under shaking in TSB medium at 28 °C; treated cells: bacteria grown in TSB medium added with 4.4% NaCl at 24 h of incubation (28 °C in shaking): (**A**) *Rahnella aquatilis* Ra4; (**B**) *Serratia plymuthica* Sp2. Different letters indicate statistically significant differences between values in 4.4% NaCl in respect to control (*p*-value < 0.05).

Figure 4 shows extracellular protein concentration present in the growth medium after 24 h of bacterial growth. *R. aquatilis* Ra4 grown in the presence of 4.4% NaCl produced a number of proteins comparable to non-stressed cells, while the concentration of protein synthesized by *S. plymuthica* Sp2 was higher in the presence of salt stress. Considering both the bacterial density at 24 h in the presence of salt (Figure 2D) and the quantity of produced proteins (Figure 4A), Ra4 showed a higher growth rate when subjected to salt stress (after 24 h of incubation) and a synthesis of proteins comparable to the control. However, similar amounts of proteins released do not necessarily mean similar protein patterns. On the contrary, the growth of Sp2 was unaffected by salt stress at 24 h, but it was coupled with a 70% increase in protein synthesis. This observation suggests that the two bacterial strains had a different sensitivity to moderate salt stress leading to a different protein expression. To explain these data, we must consider that the colony counting method used to evaluate bacterial density does not consider the number of cells that may have lost cultivability. In fact, it is well known that environmental stresses (low temperature and pH, starvation, salinity) can induce the cells to enter a Viable But Not Culturable (VBNC) state [45]. Based on this information, we can hypothesize that the density enhancement observed in Ra4 cells exposed to salinity can be overshadowed in Sp2 from the entry into the VBNC state. Consistently, Ra4 cells seem to be less sensitive than Sp2 to salt stress, while Sp2 would be more responsive in terms of protein production. To the best of our knowledge, increased protein synthesis in the presence of moderate saline stress has not been reported in the literature yet. Our work highlights that salt tolerance can lead to increased protein production. The modulation of the secretome in response to salt stress has also been reported in *Burkoldera pseudomallei* [46].

**Figure 5:**
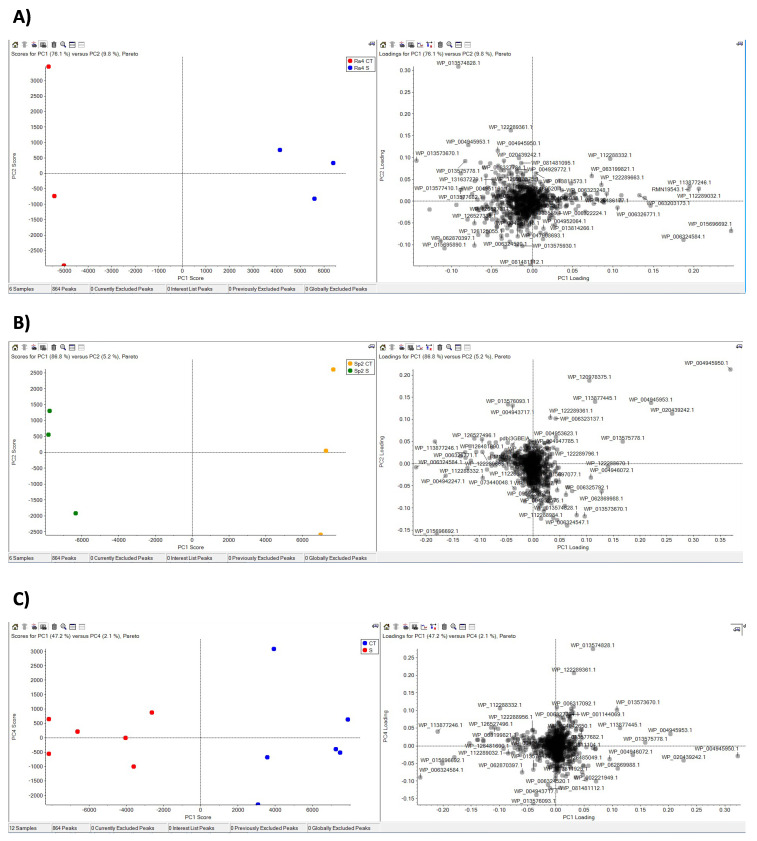
Principal Component Analysis (PCA) of the protein expression in (**A**) *Rahnella aquatilis* Ra4; (**B**) *Serratia plymuthica* Sp2, and (**C**) both bacterial strains grown in TSB medium added with 4.4% NaCl at 24 h of incubation (28 °C in shaking).

PCA analysis results of the expressed proteins are presented in Figure 5. Based on PC1, all the samples were well separated, both considering Ra4 (Figure 5A) and Sp2 (Figure 5B) or Ra4 + Sp2 (Figure 5C) responses. PC1 explained 78% of the variability for Ra4 and 87% for Sp2. The response to salt treatment was significant for both strains.

**Figure 6:**
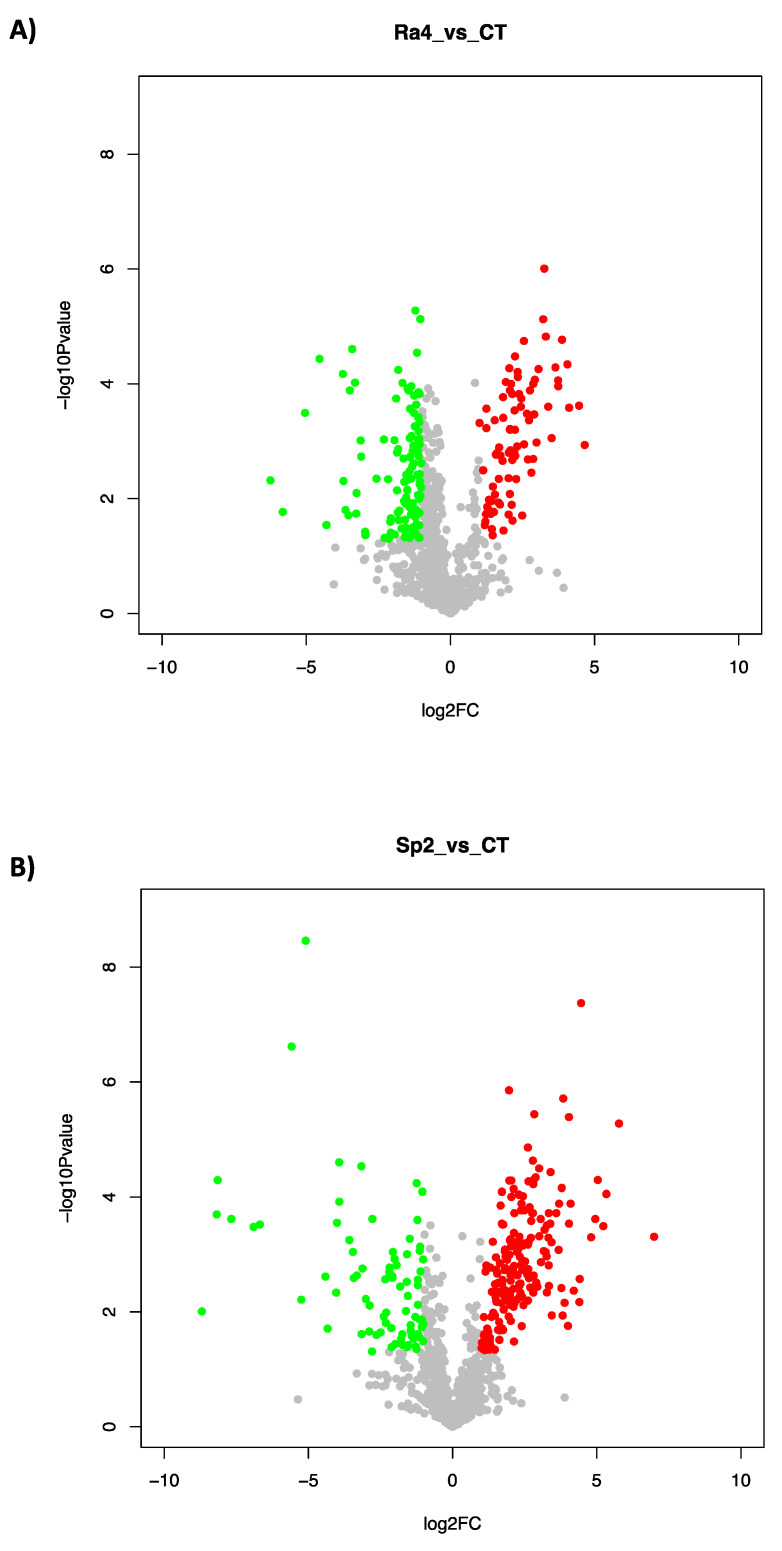
Analysis of differential proteins (DPs) in (**A**) *Rahnella aquatilis* and (**B**) *Serratia plymuthica,* 4.4% NaCl vs. Control (untreated cells). The *X*-axis represents the protein Log2fold change between the 2 groups; the *Y*-axis represents −1xlog10 *p*-value (*p*-value from *t*-test). The scattered points stand for each protein. No significant difference is indicated by grey color, significantly up-regulated and down-regulated proteins are indicated by red and green colors, respectively.

In the 2 analyzed strains, a different number of proteins was identified: 470 in Ra4 (Table S1) and 851 in Sp2 (Table S2). Among these, in Ra4, 302 proteins were significantly modulated (100 up-regulated and 202 down-regulated, Table S3), while in Sp2, 323 (206 up-regulated and 117 down-regulated, Table S4). This different protein modulation was also described in Figure 6, which shows the protein log2fold change between the two treatments. In Figure 6, scattered points stand for each protein, and no significant difference is indicated by the grey color. Moreover, significantly up-regulated and down-regulated proteins are indicated by red and green colors, respectively. Based on these results, the Sp2 strain showed a higher protein up-regulation compared to Ra4.

The results of Gene Ontology analysis showed that 116 GO terms were commonly up-regulated in the 2 analyzed strains in NaCl treated cells compared to control (Table S5). In Ra4, 26 GO terms were specifically up-regulated (Table S6) and 81 in Sp2 (Table S7). Moreover, 18 GO terms were commonly down-regulated (Table S8), and 200 and 88 GO terms were specifically down-regulated in Ra4 (Table S9) and Sp2 (Table S10), respectively.

In general, in this proteome study, also considering the GO terms, the up-regulated proteins were involved in the response to abiotic stress and the transport of solutes, while the down-regulated ones were related to the metabolism and bacterial structure and to the flagellum. Our results partly agree with Rubiano-Labrador et al. [47], identifying the proteins related to protein folding, membrane lipid alterations, signal transduction, and the transport of compatible solutes as the most involved in a 4% salt stress response of *Tistlia consotensis* (Gram-negative, aerobic, mesophilic, non-spore-forming, chemotrophic, nitrogen-fixing alpha proteobacterium [48]). In detail, our results highlight that in the Ra4 and Sp2 proteome, different trans-membrane ABC transporters (ATP-binding cassettes) were the most represented among the up-regulated proteins. Thanks to their characteristic ability to bind and hydrolyze ATP in order to transport substrates through the lipid bilayer, they play an important role in the absorption of compatible solutes in many bacteria [49]. In fact, the most up-regulated protein (fold-change 25.3 NaCl vs. Control, Table S3 in Ra4) was glycine betaine/L-proline ABC transporter substrate-binding protein ProX which mediates the import of glycine betaine, proline betaine, homobetaine, and marine osmolyte dimethylsulfoniopropionate (DMSP) and it is reported to be involved in the osmotic response in bacteria belonging to the *Bacillus* genus [50]. Other up-regulated ABC transporters were sulfate ABC transporter substrate-binding protein, zinc ABC transporter substrate-binding protein ZnuA, glutathione ABC transporter substrate-binding protein GsiB, oligopeptide ABC transporter substrate-binding protein OppA, cystine ABC transporter substrate-binding protein, and ribose ABC transporter substrate-binding protein RbsB (Table S3). Moreover, among the most up-regulated proteins, (i) the molecular chaperone OsmY acts as an autotransporter, and it is reported to be induced by osmotic stress [51]; (ii) serine endoprotease DegP is induced by stress conditions (high temperatures, extreme pH and osmotic shock) and removes or corrects damaged and/or misfolded proteins [52]; (iii) FKBP-type peptidyl-prolyl cis-trans isomerase is involved in many biological processes (such as gene expression, signal transduction, protein secretion, tissue development, and regeneration) and catalyzes protein folding [53]; (iv) glucans biosynthesis protein MdoG is one of the systems developed by enterobacteria to deal with osmotic stress [54] and finally, (v) penicillin-binding protein activator LpoB is known to regulate the peptidoglycan synthesis, thus being involved in the maintenance of osmotic stability [55].

All the up-regulated proteins in Ra4 are related to the cellular response to salinity conditions. Considering their GO terms they are involved in abiotic stress response, reactive oxygen species response, protein refolding, hyperosmotic response, and autolysis.

Among the proteins that were down-regulated under salt stress in Ra4 were: (i) the FliC/FljB family flagellin, whose down-regulation significantly decreased the diameter of the flagella [56]; (ii) the chemotaxis protein CheW, which is part of the chemotaxis system and allows bacteria to swim towards nutrients and far away from repellants [57]; (iii) deformylase peptide, cutting the terminal formyl group of proteins, which is lethal for prokaryotes [58]; (iv) flagellar hook-assembly protein FlgD, a scaffolding protein required for flagellar assembly [59], and the flagellar hook-associated protein FlgK, essential for the formation and determination of the flagellar filament length [60]; (v) phosphoenolpyruvate phosphotransferase (PTS) glucose transporter subunit IIA, a system that coordinates the bacterial response to the availability of carbohydrates through direct interactions of its components with protein targets. One of these components, the specific glucose enzyme IIA (EIIAGlc), coordinates bacterial metabolism, nutrient absorption, and interaction with cytoplasmic and membrane proteins [61].

Repression of the genes coding flagellar proteins, related to down-regulation of chemotaxis genes, has been previously observed in *Bacillus cereus* ATCC14579 exposed to severe salt stress (5% salt for 10 to 60 min) without any consequent changes in the chemotactic behavior of the treated cells [62,63]. Consistently, our results demonstrated that the down-regulation of CheW protein was also observed in *R. aquatilis* Ra4 exposed to salinity.

The most up-regulated protein in Sp2 was the thiol: disulfide interchange protein DsbA which is required for disulfide bond formation in some periplasmic proteins such as PhoA or OmpA [64]. The second most up-regulated protein was the ATP-independent periplasmic protein-refolding chaperone, which is one of the main protein families involved in cellular protection and facilitating protein folding without ATP consumption. Self-protection is the natural and immediate response of cells towards any sudden threat, such as the saline stress induced in this work. The cellular response to stress is therefore linked to molecular chaperones, which are the first line of defense to effectively recognize misfolded proteins and prevent their aggregation [65]. Further up-regulated proteins were Porin OmpA and Porin OmpC, which are associated with peptidoglycan and play a role in stabilizing the outer membrane of Gram-negative bacteria and maintaining bacterial cell shape [66]. External factors such as phosphate, nutrient deficiency, or osmolarity can affect the synthesis of proteins of the outer membrane (omps). In *E. coli* or *Salmonella typhimurium,* the synthesis of OmpC proteins is controlled by osmolarity and temperature [67,68]. Moreover, in these bacterial species, the level of OmpC increases with increasing osmolarity [69]. Osmotically-inducible lipoprotein OsmE is an osmotic regulator whose expression is induced by a high osmolarity [70]. Another up-regulated protein was FKBP-type peptidyl-prolyl cis-trans isomerase, whose function is to catalyze the folding phase of the protein. As reported before for Ra4, this enzyme is involved in different biological processes [71]. Finally, other up-regulated proteins in Sp2 were (i) the penicillin-binding protein activator LpoB, regulating the formation of peptidoglycan [55], and (ii) proteins that are sensors against various abiotic stresses, including stress induced by heavy metals and reactive oxygen species: periplasmic heavy metal sensor and oxidative stress defense protein.

As observed in Ra4, the most down-regulated proteins in Sp2 are those involved in the formation of the bacterial flagellum and involved FliC/FljB family flagellin with a log (fold change) value around −2. Only in Sp2 was it observed that the down-regulation protein is flagellar filament capping protein FliD, which is a protein that makes up the cap of the flagellum, so a reduction in function causes the bacterium to be not very mobile, as the flagellar filaments are missing [72]. Two other down-regulated proteins, as also reported before for Ra4, are flagellar hook assembly proteins FlgD and flagellar hook-associated proteins FlgK. FlgD is a scaffold protein necessary for the assembly of the flagellar hook but not for its own export [59], while FlgK controls the length of the flagellar hook and detects when it has reached its optimal length in order to initiate export [73]. Similar to what was observed in *R. aquatilis* Ra4, exposure to salt stress induced the down-regulation of proteins involved in flagellar apparatus in *S. plymutica* Sp2. However, in this bacterial species, the expression of proteins involved in chemotaxis remained unaffected by salinity. The loss of bacterial motility can be differently modulated according to salt concentration, as demonstrated in *Exiguobacterium* sp. SH31, a halotolerant bacterium isolated from sediments in Salar de Huasco (Chile).

## 4. Conclusions

This work demonstrated that taxonomically correlated non-rhizobia endophytic bacteria were present in nodules of the same plant species grown in distant geographical areas, suggesting an ecological selection of these bacteria in field conditions. In the present paper, 22 NRE bacteria were isolated, identified, and characterized for their antibiotic resistance profiles and their physiological activities, especially their response to salt stress. To contribute to filling the gap that exists in the knowledge of NRE bacteria, we analyzed and compared the proteome of two halotolerant bacterial strains (*Rahnella aquatilis* Ra4 and *Serratia plymuthica* Sp2) grown under optimal and moderate salt stress conditions. The proteomic approach employed in the present study demonstrated a conserved response to salt stress in two nodule endophytic strains belonging to different species but isolated in the same ecological niche. It is evident that proteins involved in the response to abiotic stress and the transport of solutes were up-regulated, while the down-regulated ones were related to the metabolism and structure of the bacterium, the flagellum, and the chemotaxis system. The two selected strains demonstrated different interesting features, from nitrogen-fixing ability to salt stress tolerance. For all these reasons, they represent good candidates for future investigations regarding genome characterization to go deeper into their environmental role in protecting plants from salt stress and nutrient starvation.

## Figures and Tables

**Table 1:** Characterization of different strains isolated from compared to untreated cells (green) *Vicia fava* and *Pisum sativum* nodules.

Strain	Strain Label	Host Plant	Sampling Site	Colony Description (YMA)	Gram Staining	DCP *	CAS @	IAA ^§^
FV_Fr4B	Ra1	*V. fava*	Genova	Small colony, round shape, convex elevation, entire margin, mucoid and pink	Negative	2.26	0.00	3
FV_Fr4C		*V. fava*	Genova	Small colony, round shape, convex elevation, entire margin, mucoid and pink	Negative	0.00	0.00	neg
FV_Fr5B	Ra2	*V. fava*	Genova	Small colony, round shape, convex elevation, entire margin, mucoid with pink core	Negative	2.89	0.00	1
FV_Fr6A		*V. fava*	Genova	Round shape, entire margin, mucoid and orange	Negative	0.00	0.00	neg
FV_Fr6B		*V. fava*	Genova	Small colony, round shape, convex elevation, entire margin, mucoid and pink	Negative	0.00	0.00	0
FV_EB1A_1	Sp1	*V. fava*	Calliano	Small colony, round shape, convex elevation, entire margin, mucoid with pink core	Negative	1.60	0.00	2
FV_EB2A_1		*V. fava*	Calliano	Large colony, entire margin, mucoid and pink	Negative	2.82	0.00	neg
FV_EB2C_1		*V. fava*	Calliano	Small colony, entire margin, mucoid and dark pink	Negative	0.00	0.00	neg
FV_EB1B_1	Sp2	*V. fava*	Calliano	Small colony, round shape, convex elevation, entire margin, mucoid with dark orange core	Negative	1.78	0.00	1
FV_EB1C_1		*V. fava*	Calliano	Small colony, round shape with a dark orange core	Negative	1.75	0.00	0
PS_EB1A_1		*P. sativum*	Calliano	Large colony, entire margin, mucoid and pink	Negative	3.32	1.19	neg
PS_EB1A_2		*P. sativum*	Calliano	Large colony, entire margin, mucoid and pink	Negative	2.00	0.00	2
PS_EB1C_1		*P. sativum*	Calliano	Mucoid, with a dark red core	Negative	1.58	0.00	neg
FV_EB2B_1		*V. fava*	Calliano	Mucoid, with a light pink core	Negative	2.97	0.00	neg
FV_VT2B_1	Ra3	*V. fava*	Frassineto	Small colony, round shape, convex elevation, entire margin, mucoid with pink core	Negative	2.93	0.00	2
FV_VT1C_1	Sp3	*V. fava*	Frassineto	Large colony, entire margin, mucoid and pink	Negative	2.34	0.00	0
FV_VT1B_1		*V. fava*	Frassineto	Mucoid with a red core	Negative	2.90	0.00	neg
PS_EN1B_1		*P. sativum*	Druento	Large colony, entire margin, mucoid and pink	Negative	2.08	0.00	1
PS_EN1B_2		*P. sativum*	Druento	Large colony, entire margin, mucoid and pink	Negative	1.80	0.00	neg
PS_EN1A_1		*P. sativum*	Druento	Round shape, smooth, with a dark red core	Negative	2.06	0.00	1
PS_EN1C_1	Ra4	*P. sativum*	Druento	Large colony, entire margin, mucoid with a dark red core	Negative	2.73	0.00	3
PS_EN2B_1		*P. sativum*	Druento	Large colony, entire margin, mucoid and pink with a red core	Negative	2.94	1.02	neg

* Phosphate solubilization was assessed according to Goldstein (1986) [38], using two different media: one containing dicalcium phosphate (DCP) and one containing tricalcium phosphate (TCP), after 15 days of culture. * DCP: Dicalcium phosphate; @ CAS: Siderophore production was assessed on universal Chrome Azurol S (CAS) agar (Schwyn and Neilands, 1987) [27] after 7 days of culture.; ^§^ IAA: Indole-3-Acetic Acid (IAA) production was quantified according to De Brito Alvarez et al. (1995) [28] after 3 days of culture. The values indicate the reaction intensity (0 = no color, 1 = light pink, 2 = pink, 3 = light red, 4 = red). Green background color highlights the selected strains and their characteristics.

**Table 2:** Identification, growth capabilities, and salt tolerance of the different strains isolated from fava bean and pea nodules.

Strain	Strain Label	BLAST Identification	Seq. Cov (%) ^a^	Id (%) ^b^	Genbank Accesion Number	4 °C	28 °C	37 °C	42 °C	Salt MIC (%)
FV_Fr4B	Ra1	*R. aquatilis*	99	99.3	MK157042	+	+	± ^§^	-	6.4
FV_Fr5B	Ra2	*R. aquatilis*	98	99.1	MK156743	+	+	±	-	6.4
FV_VT2B1	Ra3	*R. aquatilis*	98	99.5	MK157017	+	+	±	-	6.4
PS_EN1C1	Ra4	*R. aquatilis*	100	99.8	OK614097	+	+	±	-	5.8
FV EB 1A1	Sp1	*S. plymuthica*	98	99.5	MK156722	+	+	±	-	5.8
FV EB 1B1	Sp2	*S. plymuthica*	98	99.6	MK157024	+	+	±	-	6.2
FV VT 1C1	Sp3	*S. plymuthica*	98	99.1	MK157016	+	+	±	-	5.8

**^a^** BLAST sequence coverage obtained comparing each bacterial 16S rDNA sequence to all the reference sequences available at the NCBI. **^b^** BLAST identity results obtained comparing each bacterial 16S rDNA sequence to all the reference sequences available at the NCBI. **^§^** the use of the symbol ± stands for limited growth.

**Table 3:** Intrinsic antibiotic resistance of the different *R. aquatilis* strains, isolated from fava bean and pea nodules, determined using the disc diffusion method on solid MH medium. Values indicate the halo diameter (mm) (mean and standard errors, *n* = 3 dishes).

Antibiotic	Ra1	I ^1^	I ^2^	I ^3^	Ra2	I ^1^	I ^2^	I ^3^	Ra3	I ^1^	I ^2^	I ^3^	Ra4	I ^1^	I ^2^	I ^3^
Amoxicillin	6.0 ± 0.1			R	6.0 ± 0.1			R	6.0 ± 0.1			R	6.0 ± 0.1			R
Cefepime	6.0 ± 0.1			R	6.0 ± 0.1			R	8.0 ± 0.1			R	6.0 ± 0.1			R
Cefotaxime	18.0 ± 0.6			I	18.0 ± 0.7			I	17.0 ± 0.6			I	24.0 ± 0.8			S
Ceftazidime	22.0 ± 0.3			S	22.0 ± 0.4			S	25.0 ± 0.3			S	22.0 ± 0.5			S
Chloramphenicol	26.7 ± 1.8	S	S		26.7 ± 1.3	S	S		24.3 ± 0.3	S	S		24.0 ± 1.0	S	S	
Ciprofloxacin	34.0 ± 0.5			S	40.0 ± 0.7			S	28.0 ± 0.4			S	35.0 ± 0.6			S
Erythromycin	8.3 ± 1.8	R	R		6.3 ± 0.3	R	R		8.0 ± 0.6	R	R		6.7 ± 0.3	R	R	
Kanamycin	16.3 ± 0.9	S	S		18.3 ± 0.3	S	S		19.0 ± 0.6	S	S		16.3 ± 0.3	S	S	
Meropenem	34.0 ± 0.7			S	18.0 ± 0.6			I	28.0 ± 0.7			S	29.0 ± 0.6			S
Neomycin	10.3 ± 0.9	S	S		11.0 ± 0.6	S	S		11.7 ± 0.3	S	S		11.3 ± 0.3	S	S	
Piperacillin/Tazobactam	23.0 ± 0.5			S	22.0 ± 0.6			S	27.0 ± 0.6			S	24.0 ± 0.7			S
Rifampicin	20.0 ± 0.0		S		19.0 ± 0.5		S		21.3 ± 0.7		S		22.3 ± 0.9		S	
Streptomycin	14.0 ± 1.0	S	S		16.7 ± 0.7	S	S		15.3 ± 0.3	S	S		15.0 ± 0.0	S	S	
Trimetoprim/Sulfametoxazol	24.0 ± 0.7				32.0 ± 0.6				28.0 ± 0.6				32.0 ± 0.5			

^1^ Indicates the results interpreted according to Stock et al., 2000 [29], which state that all *Rahnella* strains are naturally sensitive or intermediate to Chloramphenicol and to aminoglycosides of which Streptomycin, Neomycin, and Kanamycin are part, intrinsically resistant to macrolides of which Erythromycin is part. ^2^ indicates the results interpreted according to Massa et al., 2000 [24]. An inhibition zone lower than 7 mm was considered as an index of no sensitivity to the tested drug. ^3^ Indicates the results interpreted according to EUCAST Clinical Breakpoint Tables v. 12. Green background color highlights the selected strains and their characteristics.

**Table 4:** Intrinsic antibiotic resistance of the different *S. plymuthica* strains, isolated from fava bean and pea nodules, determined using the disc diffusion method on solid MH medium. Values indicate the halo diameter (mm) (mean and standard errors, *n* = 3 dishes).

Antibiotic	Sp1	I ^1^	I ^2^	Sp2	I ^1^	I ^2^	Sp3	I ^1^	I ^2^
Amoxicillin	6.0 ± 0.1		R	6.0 ± 0.1		R	6.0 ± 0.1		R
Cefepime	7.0 ± 0.1		R	6.0 ± 0.1		R	6.0 ± 0.1		R
Cefotaxime	25.0 ± 0.7		S	24.0 ± 0.6		S	23.0 ± 0.7		S
Ceftazidime	22.0 ± 0.3		S	23.0 ± 0.3		S	19.0 ± 0.5		I
Chloramphenicol	17.0 ± 1.7	S		17.3 ± 1.8	S		24.0 ± 1.0	S	
Ciprofloxacin	45.0 ± 0.5		S	44.0 ± 0.4		S	36.0 ± 0.6		S
Erythromycin	13.0 ± 1.5	S		6.0 ± 0.2	R		7.0 ± 0.3	R	
Kanamycin	15.3 ± 0.7	S		17.3 ± 1.3	S		17.7 ± 0.3	S	
Meropenem	24.0 ± 0.5		S	25.0 ± 0.6		S	25.0 ± 0.6		S
Neomycin	12.3 ± 1.5	S		9.7 ± 0.9	S		11.3 ± 0.3	S	
Piperacillin/Tazobactam	22.0 ± 0.5		S	23.0 ± 0.6		S	21.0 ± 0.6		S
Rifampicin	12.7 ± 1.3	S		14.0 ± 1.2	S		22.0 ± 1.2	S	
Streptomycin	15.0 ± 1.7	S		12.7 ± 1.5	S		15.3 ± 0.3	S	
Trimetoprim/Sulfametoxazol	27.0 ± 0.8			32.0 ± 0.6			34.0 ± 0.7		

^1^ indicates the results interpreted according to Massa et al., 2000 [24]. An inhibition zone lower than 7 mm was considered as an index of no sensitivity to the tested drug. ^2^ Indicates the results interpreted according to EUCAST Clinical Breakpoint Tables v. 12.0. Green background color highlights the selected strains and their characteristics.

**Table 5:** Antibiotic response of the different strains, isolated from fava bean and pea nodules, determined using Vitek2. The results were interpreted according to EUCAST Clinical Breakpoint Tables v. 12.0.

Antibiotic	Ra1	I ^1^	Ra2	I ^1^	Ra3	I ^1^	Ra4	I ^1^	Sp1	I ^2^	Sp2	I ^2^	Sp3	I ^2^
Amikacin	<=8	S	<=8	S	<=8	S	<=8	S	<=8	S	<=8	S	<=8	S
Amoxicillin/Clavulanic Acid	<=4	S	<=4	S	<=4	S	<=4	S	<=4	S	<=4	S	4	S
Cefepime	<=1	S	<=1	S	<=0.5	S	<=1	S	<=1	S	2	I	<=1	S
Cefotaxime	<=0.5	S	<=0.5	S	<=0.5	S	<=0.5	S	-------		-------		------	
Ceftazidime	<=0.5	S	<=0.5	S	<=0.2	S	<=0.5	S	<=0.5	S	<=0.5	S	<=0.5	S
Ciprofloxacin	<=0.2	S	<=0.2	S	<=0.1	S	<=0.2	S	<=0.2	S	<=0.2	S	<=0.2	S
Ertapenem	<=0.1	S	<=0.1	S	<=0.1	S	<=0.1	S	<=0.1	S	<=0.1	S	<=0.1	S
Gentamicin	<=2	S	<=2	S	<=2	S	<=2	S	<=2	S	<=2	S	<=2	S
Meropenem	<=0.1	S	<=0.1	S			<=0.1	S	<=0.1	S	<=0.1	S	<=0.1	S
Piperacillin/Tazobactam	<=4	S	<=4	S	<=4	S	<=4	S	<=4	S	<=4	S	<=4	S
Trimetoprim/Sulfametoxazol	<=2	S	<=2	S	<=2	S	<=2	S	<=2	S	<=2	S	<=2	S
Ampicillin	<=2	S	4	S	>8	R	8	S	4	S	4	S	4	S
Aztreonam	<=1	S	<=1	S	<=1	S	<=1	S	2	I	<=1	S	-------	
Colistin	<=2	S	<=2	S	<=2	S	<=2	S	>4	R	>4	R	<=2	S
Levofloxacin	<=0.5	S	<=0.5	S	<=0.5	S	<=0.5	S	<=0.5	S	<=0.5	S	<=0.5	S
Piperacillin	<=4	S	<=4	S	<=4	S	<=4	S	<=4	S	<=4	S	<=4	S
Ticarcillin	<=8	S	<=8	S	<=8	S	<=8	S	<=8	S	<=8	S	<=8	S
Tigecycline	<=0.5	S	<=0.5	S	<=0.5	S	<=0.5	S	<=0.5	S	<=0.5	S	<=0.5	S
Tobramycin	<=2	S	<=2	S	<=2	S	<=2	S	<=2	S	<=2	S	<=2	S

^1^ Indicates the results interpreted according to Stock et al., 2000 [29], which state that all *Rahnella* strains are naturally sensitive or intermediate to Chloramphenicol and to aminoglycosides of which Streptomycin, Neomycin, and Kanamycin are part, intrinsically resistant to macrolides of which Erythromycin is part. ^2^ indicates the results interpreted according to Massa et al., 2000 [24]. An inhibition zone lower than 7 mm was considered as an index of no sensitivity to the tested drug. Green background color highlights the selected strains and their characteristics.

## Data Availability

The mass spectrometry proteomics data have been deposited with the ProteomeXchange Consortium via the PRIDE partner repository with the dataset identifier PXD031626. Submission details: Project Name: *Proteome and physiological characterization of halotolerant nodule endophytes: the case of Rahnella aquatilis and Serratia plymuthica*. Project accession: PXD031626. Reviewer account details: Username: reviewer_pxd031626@ebi.ac.uk Password: YH1Cxirc.

## Supplementary Materials

The following material is available online at https://www.mdpi.com/article/10.3390/microorganisms10050890/s1, Table S1: Identified proteins in Ra4 (*Rahnella aquatilis*); Table S2: Identified proteins in Sp2 (*Serratia plymuthica*); Table S3. Differential expression of proteins in Ra4 treated with 0.4% NaCl vs. Control; Table S4: Differential expression of proteins in Sp2 treated with 0.4% NaCl vs. Control; Table S5: GO terms of proteins overexpressed both in Ra4 and in Sp2 treated with 0.4% NaCl vs. Control. Table S6: GO terms of proteins overexpressed only in Ra4 treated with 0.4% NaCl vs. Control; Table S7: GO terms of proteins overexpressed only in Sp2 treated with 0.4% NaCl vs. Control. Table S8: GO terms of proteins under-expressed both in Ra4 and in Sp2 treated with 0.4% NaCl vs. Control; Table S9: GO terms of proteins under-expressed only in Ra4 treated with 0.4% NaCl vs. Control; Table S10: GO terms of proteins under-expressed only in Sp2 treated with 0.4% NaCl vs. Control.
