# Supplementary material for: Proteome and Physiological Characterization of Halotolerant Nodule Endophytes: The Case of Rahnella aquatilis and Serratia plymuthica
Source: Microorganisms. 2022 Apr 24;10(5):890. doi: 10.3390/microorganisms10050890 (PMC9143289; doi:10.3390/microorganisms10050890)
Supplement: Supplementary file 1 [file microorganisms-10-00890-s001.zip › microorganisms-1682220-supplementary.pdf]

Article

Proteome and physiological characterization of halotolerant nodule endophytes: the case of *Rahnella aquatilis* and *Serratia plymuthica*

Giorgia Novello[1*], Elisa Gamalero[1], Nadia Massa[1], Patrizia Cesaro[1], Guido Lingua[1], Valeria Todeschini[1], Alice Caramaschi[2], Francesco Favero[3,4], Davide Corà[3,4], Marcello Manfredi[3,4], Emilio Marengo[1], Elisa Bona[2,3§]

[1] Dipartimento di Scienze e Innovazione Tecnologica, Università del Piemonte Orientale, Alessandria, Italy; giorgia.novello@uniupo.it, nadia.massa@uniupo.it, patrizia.cesaro@uniupo.it, guido.lingua@uniupo.it, valeria.todeschini@uniupo.it, emilio.marengo@uniupo.it, elisa.gamalero@uniupo.it

[2] Dipartimento per lo Sviluppo Sostenibile e la Transizione Ecologica, Vercelli, Italy; elisa.bona@uniupo.it, alice.caramaschi@uniupo.it

[3] Center on Autoimmune and Allergic Diseases (CAAD), Università del Piemonte Orientale, Novara, Italy francesco.favero@uniupo.it; davide.cora@uniupo.it

[4] Dipartimento di Medicina Traslazionale, Università del Piemonte Orientale, Novara, Italy marcello.manfredi@uniupo.it; francesco.favero@uniupo.it; davide.cora@uniupo.it

* Correspondence: *giorgia.novello@uniupo.it; § elisa.bona@uniupo.it.

Supplementary materials:

Table S1. Identified proteins in Ra4 (*Rahnella aquatilis*).

Table S2. Identified proteins in Sp2 (*Serratia plymuthica*).

Table S3. Differential expression of proteins in Ra4 (*Rahnella aquatilis*) treated with 0.4% NaCl vs Control.

Table S4. Differential expression of proteins in Sp2 (*Serratia plymuthica*) treated with 0.4% NaCl vs Control.

Table S5. GO terms of proteins overexpressed both in Ra4 (*Rahnella aquatilis*) and in Sp2 (*Serratia plymuthica*) treated with 0.4% NaCl vs Control.

Table S6. GO terms of proteins overexpressed only in Ra4 (*Rahnella aquatilis*) treated with 0.4% NaCl vs Control.

Table S7. GO terms of proteins overexpressed only in Sp2 (*Serratia plymuthica*) treated with 0.4% NaCl vs Control.

Table S8. GO terms of proteins underexpressed both in Ra4 (*Rahnella aquatilis*) and in Sp2 (*Serratia plymuthica*) treated with 0.4% NaCl vs Control.

Table S9. GO terms of proteins underexpressed only in Ra4 (*Rahnella aquatilis*) treated with 0.4% NaCl vs Control.

Table S10. GO terms of proteins underexpressed only in Sp2 (*Serratia plymuthica*) treated with 0.4% NaCl vs Control.

Table S1. Identified proteins in Ra4 (*Rahnella aquatilis*).

Protein code	Protein identification	Reference organism
KFD02968.1	ATP-binding component of an ABC superfamily transporter	Rahnella aquatilis CIP 78.65 = ATCC 33071
KFD16213.1	hypothetical protein GRAQ_00881	Rahnella aquatilis CIP 78.65 = ATCC 33071
RKT66530.1	shikimate kinase	Rahnella aquatilis
WP_001144069.1	MULTISPECIES: 30S ribosomal protein S21	Bacteria
WP_002210155.1	MULTISPECIES: 30S ribosomal protein S18	Enterobacterales
WP_002221949.1	MULTISPECIES: transcription antiterminator/RNA stability regulator CspE	Enterobacterales
WP_002227352.1	MULTISPECIES: 50S ribosomal protein L36	Enterobacterales
WP_002919219.1	MULTISPECIES: DNA-directed RNA polymerase subunit alpha	Enterobacterales
WP_004089944.1	MULTISPECIES: integration host factor subunit alpha	Enterobacterales
WP_004093904.1	MULTISPECIES: F0F1 ATP synthase subunit C	Bacteria
WP_004391423.1	MULTISPECIES: 50S ribosomal protein L22	Enterobacterales
WP_004392084.1	MULTISPECIES: 50S ribosomal protein L33	Enterobacterales
WP_004929731.1	MULTISPECIES: 30S ribosomal protein S11	Enterobacterales
WP_004929772.1	MULTISPECIES: 30S ribosomal protein S19	Enterobacterales
WP_004931195.1	MULTISPECIES: 50S ribosomal protein L28	Enterobacterales
WP_005969574.1	MULTISPECIES: 30S ribosomal protein S7	Enterobacterales
WP_008457164.1	MULTISPECIES: 50S ribosomal protein L14	Enterobacterales
WP_009634688.1	MULTISPECIES: cell division protein FtsA	Yersiniaceae
WP_009637487.1	MULTISPECIES: 50S ribosomal protein L34	Yersiniaceae
WP_009639175.1	MULTISPECIES: 30S ribosomal protein S10	Gammaproteobacteria
WP_009639183.1	MULTISPECIES: 50S ribosomal protein L16	Yersiniaceae
WP_013573626.1	MULTISPECIES: peptidylprolyl isomerase A	Rahnella sp.
WP_013573634.1	MULTISPECIES: cAMP-activated global transcriptional regulator CRP	Yersiniaceae
WP_013573668.1	MULTISPECIES: 30S ribosomal protein S12	Yersiniaceae
WP_013573670.1	elongation factor Tu	Rahnella sp.
WP_013573688.1	MULTISPECIES: 50S ribosomal protein L4	Rahnella sp.
WP_013573689.1	MULTISPECIES: 50S ribosomal protein L23	Rahnella sp.
WP_013573691.1	MULTISPECIES: 30S ribosomal protein S3	Rahnella sp.
WP_013573692.1	MULTISPECIES: 50S ribosomal protein L29	Yersiniaceae
WP_013573695.1	MULTISPECIES: 50S ribosomal protein L5	Yersiniaceae
WP_013573696.1	MULTISPECIES: 30S ribosomal protein S14	Yersiniaceae
WP_013573697.1	MULTISPECIES: 30S ribosomal protein S8	Bacteria
WP_013573699.1	MULTISPECIES: 50S ribosomal protein L18	Yersiniaceae
WP_013573700.1	MULTISPECIES: 30S ribosomal protein S5	Yersiniaceae
WP_013573701.1	MULTISPECIES: 50S ribosomal protein L30	Enterobacterales
WP_013573702.1	MULTISPECIES: 50S ribosomal protein L15	Rahnella sp.
WP_013573704.1	MULTISPECIES: 30S ribosomal protein S13	Yersiniaceae
WP_013573706.1	MULTISPECIES: 50S ribosomal protein L17	Yersiniaceae
WP_013573778.1	MULTISPECIES: 50S ribosomal protein L9	Yersiniaceae
WP_013573791.1	MULTISPECIES: inorganic diphosphatase	Rahnella sp.
WP_013573794.1	MULTISPECIES: class 1 fructose-bisphosphatase	Rahnella sp.
WP_013573804.1	MULTISPECIES: 50S ribosomal protein L21	Rahnella sp.
WP_013573995.1	MULTISPECIES: phosphopyruvate hydratase	Rahnella sp.
WP_013574115.1	MULTISPECIES: lysine--tRNA ligase	Rahnella sp.
WP_013574196.1	MULTISPECIES: ribosome recycling factor	Rahnella sp.
WP_013574351.1	MULTISPECIES: nitrogen regulatory protein P-II	Yersiniaceae
WP_013574367.1	MULTISPECIES: nucleoside-diphosphate kinase	Yersiniaceae
WP_013574412.1	MULTISPECIES: transketolase	Rahnella sp.
WP_013574433.1	MULTISPECIES: phosphocarrier protein Hpr	Rahnella sp.
WP_013574579.1	MULTISPECIES: phosphate acetyltransferase	Rahnella sp.
WP_013574593.1	MULTISPECIES: NADH-quinone oxidoreductase subunit NuoF	Rahnella sp.
WP_013574663.1	MULTISPECIES: 1-phosphofructokinase	Rahnella sp.
WP_013574775.1	MULTISPECIES: leucine-responsive transcriptional regulator Lrp	Yersiniaceae
WP_013574828.1	formate C-acetyltransferase	Rahnella sp.
WP_013575246.1	MULTISPECIES: transcription antiterminator/RNA stability regulator CspE	Yersiniaceae
WP_013575313.1	MULTISPECIES: pyruvate kinase	Rahnella sp.
WP_013575323.1	MULTISPECIES: cell division topological specificity factor MinE	Rahnella sp.

Protein code	Protein identification	Reference organism
WP_013575324.1	MULTISPECIES: septum site-determining protein MinD	Rahnella sp.
WP_013575568.1	MULTISPECIES: DUF1471 domain-containing protein	Rahnella sp.
WP_013575778.1	MULTISPECIES: FliC/FljB family flagellin	Rahnella sp.
WP_013575910.1	MULTISPECIES: aconitate hydratase AcnA	Rahnella sp.
WP_013575930.1	MULTISPECIES: BON domain-containing protein	Rahnella sp.
WP_013575931.1	MULTISPECIES: outer membrane protein OmpW	Rahnella sp.
WP_013575948.1	MULTISPECIES: bifunctional acetaldehyde-CoA/alcohol dehydrogenase	Rahnella sp.
WP_013575950.1	MULTISPECIES: DNA-binding transcriptional regulator H-NS	Rahnella sp.
WP_013576093.1	MULTISPECIES: major outer membrane lipoprotein	Yersiniaceae
WP_013576134.1	MULTISPECIES: 50S ribosomal protein L20	Yersiniaceae
WP_013576136.1	MULTISPECIES: translation initiation factor IF-3	Yersiniaceae
WP_013576150.1	MULTISPECIES: carboxy terminal-processing peptidase	Rahnella sp.
WP_013576192.1	MULTISPECIES: PTS mannose transporter subunit IID	Rahnella sp.
WP_013576210.1	MULTISPECIES: NADP-dependent isocitrate dehydrogenase	Rahnella sp.
WP_013576245.1	MULTISPECIES: 3-oxoacyl-ACP reductase FabG	Rahnella sp.
WP_013576250.1	MULTISPECIES: 23S rRNA accumulation protein YceD	Rahnella sp.
WP_013576426.1	MULTISPECIES: peptidoglycan-associated lipoprotein Pal	Rahnella sp.
WP_013576500.1	MULTISPECIES: DUF493 family protein	Rahnella sp.
WP_013576637.1	MULTISPECIES: DNA-binding protein HU-beta	Rahnella sp.
WP_013576663.1	MULTISPECIES: transcription antitermination factor NusB	Yersiniaceae
WP_013576670.1	MULTISPECIES: preprotein translocase subunit YajC	Rahnella sp.
WP_013577239.1	MULTISPECIES: 50S ribosomal protein L13	Yersiniaceae
WP_013577291.1	MULTISPECIES: rod shape-determining protein	Yersiniaceae
WP_013577396.1	MULTISPECIES: DNA-binding protein HU-alpha	Yersiniaceae
WP_013577410.1	MULTISPECIES: 50S ribosomal protein L7/L12	Yersiniaceae
WP_013577414.1	MULTISPECIES: transcription termination/antitermination protein NusG	Rahnella sp.
WP_013577536.1	MULTISPECIES: transcription termination factor Rho	Rahnella sp.
WP_013577537.1	MULTISPECIES: thioredoxin TrxA	Yersiniaceae
WP_013577542.1	MULTISPECIES: ketol-acid reductoisomerase	Rahnella sp.
WP_013577556.1	MULTISPECIES: ABC transporter substrate-binding protein	Rahnella sp.
WP_013577593.1	MULTISPECIES: triose-phosphate isomerase	Rahnella sp.
WP_013577602.1	MULTISPECIES: protein-export chaperone SecB	Rahnella sp.
WP_013577652.1	MULTISPECIES: glutamate--ammonia ligase	Rahnella sp.
WP_013577682.1	MULTISPECIES: F0F1 ATP synthase subunit alpha	Rahnella sp.
WP_014333460.1	MULTISPECIES: universal stress protein UspA	Yersiniaceae
WP_014333586.1	elongation factor G	Rahnella aquatilis
WP_014333589.1	MULTISPECIES: bacterioferritin	Rahnella sp.
WP_014333606.1	MULTISPECIES: 30S ribosomal protein S4	Rahnella sp.
WP_014333640.1	chaperonin GroEL	Rahnella aquatilis
WP_014333667.1	50S ribosomal protein L9	Rahnella aquatilis
WP_014333671.1	bifunctional 2',3'-cyclic-nucleotide 2'-phosphodiesterase/3'-nucleotidase	Rahnella aquatilis
WP_014333680.1	HAMP domain-containing protein	Rahnella aquatilis
WP_014333706.1	MULTISPECIES: 30S ribosomal protein S15	Rahnella sp.
WP_014333707.1	polyribonucleotide nucleotidyltransferase	Rahnella aquatilis
WP_014411489.1	MULTISPECIES: glycerol-3-phosphate dehydrogenase	Rahnella sp.
WP_014411563.1	30S ribosomal protein S6	Rahnella aquatilis
WP_014416698.1	MULTISPECIES: NAD-dependent malic enzyme	Rahnella sp.
WP_015689898.1	MULTISPECIES: thiol peroxidase	Rahnella sp.
WP_015690067.1	aconitate hydratase AcnA	Rahnella aquatilis
WP_015690190.1	MULTISPECIES: acyl carrier protein	Yersiniaceae
WP_015690312.1	MULTISPECIES: protein translocase subunit SecD	Rahnella sp.
WP_015690520.1	50S ribosomal protein L13	Rahnella aquatilis
WP_015695859.1	outer membrane channel protein TolC	Rahnella aquatilis
WP_015695890.1	phosphopyruvate hydratase	Rahnella aquatilis
WP_015695926.1	S-ribosylhomocysteine lyase	Rahnella aquatilis
WP_015695958.1	ATP-dependent chaperone ClpB	Rahnella aquatilis
WP_015695975.1	glycine betaine/L-proline ABC transporter substrate-binding protein ProX	Rahnella aquatilis
WP_015696061.1	NADP(H)-dependent aldo-keto reductase	Rahnella aquatilis

Protein code	Protein identification	Reference organism
WP_015696082.1	LOG family protein	Rahnella aquatilis
WP_015696269.1	IMP dehydrogenase	Rahnella aquatilis
WP_015696270.1	glutamine-hydrolyzing GMP synthase	Rahnella aquatilis
WP_015696299.1	transketolase	Rahnella aquatilis
WP_015696300.1	transaldolase	Rahnella aquatilis
WP_015696316.1	phosphoenolpyruvate-protein phosphotransferase PtsI	Rahnella aquatilis
WP_015696434.1	amidophosphoribosyltransferase	Rahnella aquatilis
WP_015696452.1	acetate kinase	Rahnella aquatilis
WP_015696607.1	thioredoxin-disulfide reductase	Rahnella aquatilis
WP_015696611.1	MULTISPECIES: serine--tRNA ligase	Rahnella sp.
WP_015696652.1	formate transporter FocA	Rahnella aquatilis
WP_015696657.1	(d)CMP kinase	Rahnella aquatilis
WP_015696675.1	asparagine--tRNA ligase	Rahnella aquatilis
WP_015696682.1	quinone-dependent dihydroorotate dehydrogenase	Rahnella aquatilis
WP_015696692.1	porin OmpA	Rahnella aquatilis
WP_015696862.1	aspartate--tRNA ligase	Rahnella aquatilis
WP_015697074.1	UDP-glucose/GDP-mannose dehydrogenase family protein	Rahnella aquatilis
WP_015697077.1	bifunctional acetaldehyde-CoA/alcohol dehydrogenase	Rahnella aquatilis
WP_015697179.1	glucose-6-phosphate dehydrogenase	Rahnella aquatilis
WP_015697208.1	MULTISPECIES: PrkA family serine protein kinase	Rahnella sp.
WP_015697217.1	NAD(P)H nitroreductase	Rahnella aquatilis
WP_015697381.1	Re/Si-specific NAD(P)(+) transhydrogenase subunit beta	Rahnella aquatilis
WP_015697784.1	transcriptional regulator SlyA	Rahnella aquatilis
WP_015697791.1	MULTISPECIES: lactoylglutathione lyase	Rahnella sp.
WP_015697793.1	MULTISPECIES: Grx4 family monothiol glutaredoxin	Rahnella sp.
WP_015697836.1	phenylalanine--tRNA ligase subunit alpha	Rahnella aquatilis
WP_015697837.1	threonine--tRNA ligase	Rahnella aquatilis
WP_015697849.1	carboxy terminal-processing peptidase	Rahnella aquatilis
WP_015697850.1	MULTISPECIES: RNA chaperone ProQ	Rahnella sp.
WP_015697878.1	ATP-dependent RNA helicase DbpA	Rahnella aquatilis
WP_015697886.1	PTS mannose transporter subunit IID	Rahnella aquatilis
WP_015697909.1	adenylosuccinate lyase	Rahnella aquatilis
WP_015697921.1	alpha/beta hydrolase	Rahnella aquatilis
WP_015697927.1	PTS glucose transporter subunit IIBC	Rahnella aquatilis
WP_015697933.1	beta-ketoacyl-ACP synthase II	Rahnella aquatilis
WP_015697935.1	ketoacyl-ACP synthase III	Rahnella aquatilis
WP_015697963.1	glucan biosynthesis protein G	Rahnella aquatilis
WP_015698105.1	Tol-Pal system protein TolB	Rahnella aquatilis
WP_015698114.1	succinate dehydrogenase iron-sulfur subunit	Rahnella aquatilis
WP_015698140.1	MULTISPECIES: ferric iron uptake transcriptional regulator	Rahnella sp.
WP_015698255.1	phosphoribosylformylglycinamidine cyclo-ligase	Rahnella aquatilis
WP_015698322.1	MULTISPECIES: endopeptidase La	Rahnella sp.
WP_015698812.1	leucyl aminopeptidase	Rahnella aquatilis
WP_015698880.1	stringent starvation protein A	Rahnella aquatilis
WP_015698881.1	30S ribosomal protein S9	Rahnella aquatilis
WP_015698929.1	acetyl-CoA carboxylase biotin carboxyl carrier protein	Rahnella aquatilis
WP_015698930.1	acetyl-CoA carboxylase biotin carboxylase subunit	Rahnella aquatilis
WP_015699039.1	MULTISPECIES: DNA-directed RNA polymerase subunit beta	Rahnella sp.
WP_015699040.1	50S ribosomal protein L1	Rahnella aquatilis
WP_015699041.1	50S ribosomal protein L11	Rahnella aquatilis
WP_015699185.1	HslU--HslV peptidase ATPase subunit	Rahnella aquatilis
WP_015699238.1	MULTISPECIES: DNA-directed RNA polymerase subunit omega	Rahnella sp.
WP_015699278.1	MULTISPECIES: F0F1 ATP synthase subunit beta	Rahnella sp.
WP_015699282.1	glutamine--fructose-6-phosphate transaminase (isomerizing)	Rahnella aquatilis
WP_037033577.1	MULTISPECIES: transaldolase	Rahnella sp.
WP_037035133.1	MULTISPECIES: proline--tRNA ligase	Rahnella sp.
WP_037035301.1	MULTISPECIES: pyruvate kinase PykF	Rahnella sp.
WP_037035864.1	MULTISPECIES: ATP-independent periplasmic protein-refolding chaperone	Rahnella sp.
WP_037037332.1	MULTISPECIES: ACP S-malonyltransferase	Rahnella sp.
WP_037038559.1	NADH-quinone oxidoreductase subunit C/D	Rahnella aquatilis
WP_041673196.1	MULTISPECIES: NADH-quinone oxidoreductase subunit NuoE	Rahnella sp.

Protein code	Protein identification	Reference organism
WP_047605728.1	purine-nucleoside phosphorylase	Rahnella aquatilis
WP_047605737.1	zinc-binding alcohol dehydrogenase family protein	Rahnella aquatilis
WP_047606028.1	L,D-transpeptidase family protein	Rahnella aquatilis
WP_047606070.1	alcohol dehydrogenase AdhP	Rahnella aquatilis
WP_047606344.1	aminopeptidase N	Rahnella aquatilis
WP_047606403.1	aspartate aminotransferase family protein	Rahnella aquatilis
WP_047606593.1	DNA topoisomerase (ATP-hydrolyzing) subunit B	Rahnella aquatilis
WP_047606852.1	3-deoxy-8-phosphooctulonate synthase	Rahnella aquatilis
WP_047607346.1	aminopeptidase PepB	Rahnella aquatilis
WP_047607355.1	serine hydroxymethyltransferase	Rahnella aquatilis
WP_047607742.1	trifunctional transcriptional regulator/proline dehydrogenase/L-glutamate gamma-semialdehyde dehydrogenase	Rahnella aquatilis
WP_047607744.1	cystine ABC transporter substrate-binding protein	Rahnella aquatilis
WP_047608290.1	glutamate--ammonia ligase	Rahnella aquatilis
WP_047608317.1	transketolase	Rahnella aquatilis
WP_047608551.1	ABC transporter substrate-binding protein	Rahnella aquatilis
WP_047608595.1	NADH-quinone oxidoreductase subunit NuoG	Rahnella aquatilis
WP_047608630.1	50S ribosomal protein L3	Rahnella aquatilis
WP_047608693.1	DNA starvation/stationary phase protection protein Dps	Rahnella aquatilis
WP_047608834.1	N-acetylglucosamine-6-phosphate deacetylase	Rahnella aquatilis
WP_047608839.1	LPS assembly lipoprotein LptE	Rahnella aquatilis
WP_047608889.1	phosphoribosylaminoimidazolesuccinocarboxamide synthase	Rahnella aquatilis
WP_047609265.1	iron-sulfur cluster carrier protein ApbC	Rahnella aquatilis
WP_047609285.1	NADP-dependent phosphogluconate dehydrogenase	Rahnella aquatilis
WP_047609302.1	chemotaxis protein CheW	Rahnella aquatilis
WP_047609330.1	protein phosphatase CheZ	Rahnella aquatilis
WP_047609634.1	glutathione transferase GstA	Rahnella aquatilis
WP_047609790.1	murein transglycosylase	Rahnella aquatilis
WP_047609795.1	phosphopentomutase	Rahnella aquatilis
WP_047609862.1	aspartate-semialdehyde dehydrogenase	Rahnella aquatilis
WP_047609936.1	glycine--tRNA ligase subunit beta	Rahnella aquatilis
WP_047611609.1	lysine--tRNA ligase	Rahnella aquatilis
WP_047612217.1	excinuclease ABC subunit UvrA	Rahnella aquatilis
WP_047612647.1	ribonuclease R	Rahnella aquatilis
WP_047612656.1	translation initiation factor IF-2	Rahnella aquatilis
WP_047612675.1	anaerobic ribonucleoside-triphosphate reductase	Rahnella aquatilis
WP_047612676.1	alpha,alpha-phosphotrehalase	Rahnella aquatilis
WP_047612794.1	assimilatory sulfite reductase (NADPH) hemoprotein subunit	Rahnella aquatilis
WP_047612859.1	pitrilysin	Rahnella aquatilis
WP_052184388.1	MetQ/NlpA family lipoprotein	Rahnella aquatilis
WP_056772785.1	MULTISPECIES: 30S ribosomal protein S16	Yersiniaceae
WP_056778537.1	MULTISPECIES: 1-phosphofructokinase	Yersiniaceae
WP_056781756.1	MULTISPECIES: DUF3461 family protein	Yersiniaceae
WP_071823626.1	MULTISPECIES: ribose-phosphate pyrophosphokinase	Rahnella sp.
WP_081481095.1	MULTISPECIES: co-chaperone GroES	Yersiniaceae
WP_081481112.1	MULTISPECIES: transcription antiterminator/RNA stability regulator CspE	Rahnella sp.
WP_086935265.1	MULTISPECIES: 3-hydroxyacyl-ACP dehydratase FabZ	Rahnella sp.
WP_086935273.1	MULTISPECIES: glyceraldehyde-3-phosphate dehydrogenase	Yersiniaceae
WP_086935295.1	30S ribosomal protein S1	Rahnella aquatilis
WP_095923131.1	MULTISPECIES: D-sedoheptulose 7-phosphate isomerase	Rahnella sp.
WP_095923207.1	MULTISPECIES: 3-hydroxyacyl-ACP dehydratase FabZ	Rahnella sp.
WP_095923217.1	MULTISPECIES: 30S ribosomal protein S2	Rahnella sp.
WP_095923499.1	MULTISPECIES: 50S ribosomal protein L19	Rahnella sp.
WP_095923752.1	MULTISPECIES: 50S ribosomal protein L24	Rahnella sp.
WP_095924049.1	MULTISPECIES: F0F1 ATP synthase subunit beta	Rahnella sp.
WP_095924051.1	MULTISPECIES: F0F1 ATP synthase subunit B	Rahnella sp.
WP_095924286.1	DNA-directed RNA polymerase subunit beta'	Rahnella sp.
WP_095924349.1	MULTISPECIES: single-stranded DNA-binding protein	Rahnella sp.
WP_095924927.1	MULTISPECIES: peroxiredoxin C	Rahnella sp.
WP_095924936.1	MULTISPECIES: 6,7-dimethyl-8-ribityllumazine synthase	Rahnella sp.
WP_095925026.1	MULTISPECIES: uracil phosphoribosyltransferase	Rahnella sp.

Protein code	Protein identification	Reference organism
WP_095925051.1	MULTISPECIES: bifunctional methylenetetrahydrofolate dehydrogenase/methenyltetrahydrofolate cyclohydrolase FolD	Rahnella sp.
WP_101077378.1	MULTISPECIES: succinate--CoA ligase subunit alpha	Rahnella sp.
WP_101079205.1	MULTISPECIES: acetyl-CoA carboxylase carboxyl transferase subunit alpha	Rahnella sp.
WP_101079222.1	MULTISPECIES: 2,3,4,5-tetrahydropyridine-2,6-dicarboxylate N-succinyltransferase	Rahnella sp.
WP_112151011.1	MULTISPECIES: thymidine phosphorylase	Rahnella sp.
WP_112151804.1	MULTISPECIES: aspartate--tRNA ligase	Rahnella sp.
WP_112151951.1	MULTISPECIES: glutathione-disulfide reductase	Rahnella sp.
WP_112152041.1	MULTISPECIES: UDP-N-acetylmuramate:L-alanyl-gamma-D-glutamyl-meso-diaminopimelate ligase	Rahnella sp.
WP_112152056.1	MULTISPECIES: alpha,alpha-phosphotrehalase	Rahnella sp.
WP_112152149.1	MULTISPECIES: PTS mannose transporter subunit IIAB	Rahnella sp.
WP_112168192.1	MULTISPECIES: two-component system response regulator ArcA	Rahnella sp.
WP_112197122.1	MULTISPECIES: undecaprenyl-phosphate 4-deoxy-4-formamido-L-arabinose transferase	Rahnella sp.
WP_112197123.1	MULTISPECIES: bifunctional UDP-4-amino-4-deoxy-L-arabinose formyltransferase/UDP-glucuronic acid oxidase ArnA	Rahnella sp.
WP_112198666.1	MULTISPECIES: ribonucleotide-diphosphate reductase subunit beta	Rahnella sp.
WP_112198685.1	MULTISPECIES: redox-regulated ATPase YchF	Rahnella sp.
WP_112286789.1	MULTISPECIES: translation initiation factor IF-2	Rahnella sp.
WP_112286790.1	MULTISPECIES: transcription termination/antitermination protein NusA	Rahnella sp.
WP_112286795.1	MULTISPECIES: ATP-dependent zinc metalloprotease FtsH	Rahnella sp.
WP_112286828.1	MULTISPECIES: protease modulator HflC	Rahnella sp.
WP_112286849.1	MULTISPECIES: signal recognition particle protein	Rahnella sp.
WP_112286851.1	MULTISPECIES: S-ribosylhomocysteine lyase	Rahnella sp.
WP_112286910.1	MULTISPECIES: bifunctional aconitate hydratase 2/2-methylisocitrate dehydratase	Rahnella sp.
WP_112286922.1	MULTISPECIES: dihydrolipoyl dehydrogenase	Rahnella sp.
WP_112286924.1	MULTISPECIES: pyruvate dehydrogenase (acetyl-transferring), homodimeric type	Rahnella sp.
WP_112286939.1	MULTISPECIES: preprotein translocase subunit SecA	Rahnella sp.
WP_112286943.1	MULTISPECIES: cell division protein FtsZ	Rahnella sp.
WP_112286987.1	MULTISPECIES: carbamoyl-phosphate synthase large subunit	Rahnella sp.
WP_112287000.1	MULTISPECIES: molecular chaperone DnaJ	Rahnella sp.
WP_112287226.1	MULTISPECIES: heat shock chaperone IbpA	Rahnella sp.
WP_112287456.1	MULTISPECIES: ABC transporter substrate-binding protein	Rahnella sp.
WP_112287489.1	MULTISPECIES: protein translocase subunit SecD	Rahnella sp.
WP_112287507.1	MULTISPECIES: trigger factor	Rahnella sp.
WP_112287508.1	MULTISPECIES: ATP-dependent protease ATP-binding subunit ClpX	Rahnella sp.
WP_112287544.1	MULTISPECIES: adenylate kinase	Rahnella sp.
WP_112287579.1	MULTISPECIES: thioredoxin-dependent thiol peroxidase	Rahnella sp.
WP_112287581.1	MULTISPECIES: outer membrane protein assembly factor BamC	Rahnella sp.
WP_112287628.1	MULTISPECIES: D-alanyl-D-alanine carboxypeptidase DacA	Rahnella sp.
WP_112287808.1	MULTISPECIES: RNA polymerase-binding protein DksA	Rahnella sp.
WP_112287850.1	MULTISPECIES: elongation factor P-like protein YeiP	Rahnella sp.
WP_112288137.1	MULTISPECIES: 50S ribosomal protein L2	Rahnella sp.
WP_112288138.1	MULTISPECIES: 50S ribosomal protein L6	Rahnella sp.
WP_112288147.1	MULTISPECIES: peptide deformylase	Rahnella sp.
WP_112288177.1	MULTISPECIES: autonomous glycyl radical cofactor GrcA	Rahnella sp.
WP_112288325.1	MULTISPECIES: elongation factor Ts	Rahnella sp.
WP_112288331.1	MULTISPECIES: outer membrane protein assembly factor BamA	Rahnella sp.
WP_112288332.1	MULTISPECIES: molecular chaperone Skp	Rahnella sp.
WP_112288368.1	MULTISPECIES: LPS export ABC transporter ATP-binding protein	Rahnella sp.
WP_112288379.1	MULTISPECIES: UDP-N-acetylglucosamine 1-carboxyvinyltransferase	Rahnella sp.
WP_112288384.1	MULTISPECIES: stringent starvation protein A	Rahnella sp.
WP_112288545.1	MULTISPECIES: 50S ribosomal protein L10	Rahnella sp.
WP_112288599.1	MULTISPECIES: peptidylprolyl isomerase	Rahnella sp.
WP_112288878.1	MULTISPECIES: energy-dependent translational throttle protein EttA	Rahnella sp.
WP_112288890.1	MULTISPECIES: transketolase	Rahnella sp.

Protein code	Protein identification	Reference organism
WP_112288984.1	MULTISPECIES: class II fructose-bisphosphate aldolase	Rahnella sp.
WP_112288996.1	MULTISPECIES: methionine adenosyltransferase	Rahnella sp.
WP_112289028.1	MULTISPECIES: thymidine phosphorylase	Rahnella sp.
WP_112289029.1	MULTISPECIES: deoxyribose-phosphate aldolase	Rahnella sp.
WP_112289032.1	MULTISPECIES: molecular chaperone OsmY	Rahnella sp.
WP_112289179.1	MULTISPECIES: citrate synthase	Rahnella sp.
WP_112289181.1	MULTISPECIES: succinate dehydrogenase flavoprotein subunit	Rahnella sp.
WP_112289184.1	MULTISPECIES: cytochrome ubiquinol oxidase subunit I	Rahnella sp.
WP_112289187.1	MULTISPECIES: Tol-Pal system protein TolB	Rahnella sp.
WP_112289283.1	MULTISPECIES: thiol:disulfide interchange protein DsbA	Rahnella sp.
WP_112289290.1	MULTISPECIES: ribosome-dependent GTPase TypA	Rahnella sp.
WP_112289323.1	MULTISPECIES: ADP-glyceromanno-heptose 6-epimerase	Rahnella sp.
WP_112289334.1	MULTISPECIES: 6-phosphofructokinase	Rahnella sp.
WP_112289342.1	MULTISPECIES: glycerol kinase GlpK	Rahnella sp.
WP_112289354.1	MULTISPECIES: phosphoenolpyruvate carboxylase	Rahnella sp.
WP_112289432.1	MULTISPECIES: outer membrane protein OmpX	Rahnella sp.
WP_112289497.1	MULTISPECIES: DNA topoisomerase (ATP-hydrolyzing) subunit A	Rahnella sp.
WP_112289500.1	MULTISPECIES: ribonucleoside-diphosphate reductase subunit alpha	Rahnella sp.
WP_112289754.1	MULTISPECIES: chaperonin GroEL	Rahnella sp.
WP_112289929.1	MULTISPECIES: NADH-quinone oxidoreductase subunit NuoF	Rahnella sp.
WP_112290300.1	MULTISPECIES: ATP-dependent RNA helicase RhlB	Rahnella sp.
WP_112290409.1	MULTISPECIES: acetyl-CoA carboxylase biotin carboxylase subunit	Rahnella sp.
WP_112290456.1	MULTISPECIES: glycine betaine/L-proline ABC transporter substrate-binding protein ProX	Rahnella sp.
WP_112290596.1	MULTISPECIES: outer membrane protein assembly factor BamD	Rahnella sp.
WP_112290698.1	MULTISPECIES: sulfate ABC transporter substrate-binding protein	Rahnella sp.
WP_112290707.1	MULTISPECIES: PTS glucose transporter subunit IIA	Rahnella sp.
WP_112290709.1	MULTISPECIES: phosphoenolpyruvate-protein phosphotransferase PtsI	Rahnella sp.
WP_112290710.1	MULTISPECIES: cysteine synthase A	Rahnella sp.
WP_112290844.1	MULTISPECIES: ribose ABC transporter substrate-binding protein RbsB	Rahnella sp.
WP_112290861.1	MULTISPECIES: F0F1 ATP synthase subunit gamma	Rahnella sp.
WP_112290864.1	MULTISPECIES: glutamine--fructose-6-phosphate transaminase (isomerizing)	Rahnella sp.
WP_112290918.1	MULTISPECIES: xanthine phosphoribosyltransferase	Rahnella sp.
WP_112290958.1	MULTISPECIES: 4-hydroxy-3-polyprenylbenzoate decarboxylase	Rahnella sp.
WP_112291457.1	MULTISPECIES: recombinase RecA	Rahnella sp.
WP_112291485.1	MULTISPECIES: nucleoside-specific channel-forming protein Tsx	Rahnella sp.
WP_112291869.1	MULTISPECIES: 2,3-diphosphoglycerate-dependent phosphoglycerate mutase	Rahnella sp.
WP_112291902.1	MULTISPECIES: transaldolase	Rahnella sp.
WP_113875968.1	MULTISPECIES: serine hydroxymethyltransferase	Rahnella sp.
WP_113875973.1	inositol-1-monophosphatase	Rahnella aquatilis
WP_113875978.1	MULTISPECIES: histidine--tRNA ligase	Rahnella sp.
WP_113875981.1	MULTISPECIES: ribosome biogenesis GTPase Der	Rahnella sp.
WP_113876027.1	MULTISPECIES: ADP-forming succinate--CoA ligase subunit beta	Rahnella sp.
WP_113876048.1	glutamine--tRNA ligase	Rahnella aquatilis
WP_113876050.1	MULTISPECIES: N-acetylglucosamine-6-phosphate deacetylase	Rahnella sp.
WP_113876055.1	MULTISPECIES: PhoH family protein	Rahnella sp.
WP_113876058.1	MULTISPECIES: leucine--tRNA ligase	Rahnella sp.
WP_113876146.1	MULTISPECIES: cytochrome o ubiquinol oxidase subunit I	Rahnella sp.
WP_113876187.1	MULTISPECIES: elongation factor 4	Rahnella sp.
WP_113876194.1	MULTISPECIES: nucleotide exchange factor GrpE	Rahnella sp.
WP_113876237.1	HD domain-containing protein	Rahnella aquatilis
WP_113876297.1	phosphopentomutase	Rahnella aquatilis
WP_113876315.1	isoleucine--tRNA ligase	Rahnella aquatilis
WP_113876357.1	MULTISPECIES: pyruvate dehydrogenase complex dihydrolipoyllysine-residue acetyltransferase	Rahnella sp.
WP_113876396.1	MULTISPECIES: serine endoprotease DegP	Rahnella sp.
WP_113876539.1	MetQ/NlpA family lipoprotein	Rahnella aquatilis
WP_113876559.1	phage tail tape measure protein	Rahnella aquatilis

Protein code	Protein identification	Reference organism
WP_113876669.1	MULTISPECIES: glucose-6-phosphate isomerase	Rahnella sp.
WP_113876759.1	MULTISPECIES: valine--tRNA ligase	Rahnella sp.
WP_113876789.1	lysine-sensitive aspartokinase 3	Rahnella aquatilis
WP_113876814.1	MULTISPECIES: NAD(P)H-dependent oxidoreductase	Rahnella sp.
WP_113876859.1	MULTISPECIES: superoxide dismutase Mn	Rahnella sp.
WP_113876926.1	peptidyl-dipeptidase Dcp	Rahnella aquatilis
WP_113876936.1	MULTISPECIES: glutathione-disulfide reductase	Rahnella sp.
WP_113876955.1	glutamate--tRNA ligase	Rahnella aquatilis
WP_113877028.1	MULTISPECIES: acetyl-CoA carboxylase, carboxyltransferase subunit beta	Rahnella sp.
WP_113877044.1	acetate kinase	Rahnella aquatilis
WP_113877045.1	MULTISPECIES: sugar phosphatase	Rahnella sp.
WP_113877047.1	MULTISPECIES: pyridoxal phosphate-dependent aminotransferase	Rahnella sp.
WP_113877056.1	MULTISPECIES: catalase	Rahnella sp.
WP_113877073.1	aspartate ammonia-lyase	Rahnella aquatilis
WP_113877087.1	MULTISPECIES: FtsH protease activity modulator HflK	Rahnella sp.
WP_113877088.1	MULTISPECIES: adenylosuccinate synthase	Rahnella sp.
WP_113877103.1	MULTISPECIES: malate dehydrogenase	Rahnella sp.
WP_113877112.1	MULTISPECIES: polyribonucleotide nucleotidyltransferase	Rahnella sp.
WP_113877113.1	MULTISPECIES: DEAD/DEAH family ATP-dependent RNA helicase	Rahnella sp.
WP_113877151.1	maltose/maltodextrin ABC transporter substrate-binding protein MalE	Rahnella aquatilis
WP_113877173.1	DUF853 domain-containing protein	Rahnella aquatilis
WP_113877246.1	FKBP-type peptidyl-prolyl cis-trans isomerase	Rahnella aquatilis
WP_113877256.1	diaminobutyrate--2-oxoglutarate transaminase	Rahnella aquatilis
WP_113877369.1	RNA polymerase sigma factor RpoD	Rahnella aquatilis
WP_113877382.1	DNA topoisomerase IV subunit A	Rahnella aquatilis
WP_113877398.1	CTP synthase (glutamine hydrolyzing)	Rahnella aquatilis
WP_113877411.1	MULTISPECIES: murein hydrolase activator NlpD	Rahnella sp.
WP_113877444.1	FliC/FljB family flagellin	Rahnella aquatilis
WP_113877445.1	FliC/FljB family flagellin	Rahnella aquatilis
WP_113877475.1	MULTISPECIES: fused PTS fructose transporter subunit IIA/HPr protein	Rahnella sp.
WP_113877481.1	MULTISPECIES: porin	Rahnella sp.
WP_113877533.1	glycine betaine/L-proline ABC transporter substrate-binding protein ProX	Rahnella aquatilis
WP_113877598.1	N-acetylmuramoyl-L-alanine amidase	Rahnella aquatilis
WP_113877602.1	pitrilysin	Rahnella aquatilis
WP_113877622.1	MULTISPECIES: sulfate ABC transporter substrate-binding protein	Rahnella sp.
WP_113877630.1	glycine C-acetyltransferase	Rahnella aquatilis
WP_113877644.1	MULTISPECIES: orotate phosphoribosyltransferase	Rahnella sp.
WP_113877923.1	3-deoxy-7-phosphoheptulonate synthase AroG	Rahnella aquatilis
WP_113878003.1	MULTISPECIES: NAD(P)H-flavin reductase	Rahnella sp.
WP_113878010.1	5-methyltetrahydropteroyltriglutamate--homocysteine S-methyltransferase	Rahnella aquatilis
WP_113878090.1	MULTISPECIES: DNA helicase II	Rahnella sp.
WP_113878108.1	ketol-acid reductoisomerase	Rahnella aquatilis
WP_113878162.1	phosphomannomutase CpsG	Rahnella aquatilis
WP_113878167.1	class I fructose-bisphosphate aldolase	Rahnella aquatilis
WP_113878210.1	proline--tRNA ligase	Rahnella aquatilis
WP_113878222.1	UMP kinase	Rahnella aquatilis
WP_113878238.1	transketolase	Rahnella aquatilis
WP_113878239.1	MULTISPECIES: phosphoglycerate kinase	Rahnella sp.
WP_113878243.1	MULTISPECIES: oxidative stress defense protein	Rahnella sp.
WP_113878247.1	phosphoglycerate dehydrogenase	Rahnella aquatilis
WP_113878254.1	aminomethyl-transferring glycine dehydrogenase	Rahnella aquatilis
WP_113878291.1	MULTISPECIES: Trk system potassium transporter TrkA	Rahnella sp.
WP_119261052.1	DNA topoisomerase (ATP-hydrolyzing) subunit B	Rahnella aquatilis
WP_119261115.1	two-component system response regulator OmpR	Rahnella aquatilis
WP_119261123.1	nitrite reductase large subunit	Rahnella aquatilis
WP_119261152.1	succinate dehydrogenase/fumarate reductase iron-sulfur subunit	Rahnella aquatilis
WP_119261153.1	fumarate reductase (quinol) flavoprotein subunit	Rahnella aquatilis

Protein code	Protein identification	Reference organism
WP_119261168.1	DEAD/DEAH family ATP-dependent RNA helicase	Rahnella aquatilis
WP_119261248.1	alanine--tRNA ligase	Rahnella aquatilis
WP_119261297.1	MetQ/NlpA family lipoprotein	Rahnella aquatilis
WP_119261318.1	glutamate 5-kinase	Rahnella aquatilis
WP_119261434.1	aspartate/tyrosine/aromatic aminotransferase	Rahnella aquatilis
WP_119261438.1	bifunctional 3-hydroxydecanoyl-ACP dehydratase/trans-2-decenoyl-ACP isomerase	Rahnella aquatilis
WP_119261653.1	Re/Si-specific NAD(P)(+) transhydrogenase subunit alpha	Rahnella aquatilis
WP_119261728.1	porin OmpC	Rahnella aquatilis
WP_119261854.1	alpha-D-glucose phosphate-specific phosphoglucomutase	Rahnella aquatilis
WP_119261859.1	lipoyl synthase	Rahnella aquatilis
WP_119261922.1	glycine cleavage system aminomethyltransferase GcvT	Rahnella aquatilis
WP_119261924.1	ribose-5-phosphate isomerase RpiA	Rahnella aquatilis
WP_119262138.1	Do family serine endopeptidase	Rahnella aquatilis
WP_119262197.1	triose-phosphate isomerase	Rahnella aquatilis
WP_121019126.1	bifunctional phosphoribosylaminoimidazolecarboxamide formyltransferase/IMP cyclohydrolase	Rahnella aquatilis
WP_121019215.1	oligopeptide ABC transporter substrate-binding protein OppA	Rahnella aquatilis
WP_121019217.1	tryptophan synthase subunit beta	Rahnella aquatilis
WP_121019425.1	chemotaxis protein CheA	Rahnella aquatilis
WP_121019630.1	MULTISPECIES: porin OmpC	Rahnella sp.
WP_121019727.1	polysaccharide export protein	Rahnella aquatilis
WP_121019733.1	phosphomannomutase CpsG	Rahnella aquatilis
WP_121019993.1	phosphoenolpyruvate carboxykinase (ATP)	Rahnella aquatilis
WP_121020019.1	MULTISPECIES: insulinase family protein	Rahnella sp.
WP_121020032.1	glycine--tRNA ligase subunit beta	Rahnella aquatilis
WP_121020042.1	2-oxoglutarate dehydrogenase E1 component	Rahnella aquatilis
WP_121020187.1	PLP-dependent aminotransferase family protein	Rahnella aquatilis
WP_126124167.1	phospholipid-binding lipoprotein MlaA	Rahnella aquatilis
WP_126124176.1	ATP phosphoribosyltransferase	Rahnella aquatilis
WP_126124308.1	preprotein translocase subunit SecA	Rahnella aquatilis
WP_126124580.1	oligopeptidase A	Rahnella aquatilis
WP_126124653.1	beta-Ala-His dipeptidase	Rahnella aquatilis
WP_126124678.1	transaldolase	Rahnella aquatilis
WP_126124886.1	DUF945 domain-containing protein	Rahnella aquatilis
WP_126124940.1	iron uptake system protein EfeO	Rahnella aquatilis
WP_126124945.1	GrxA family glutaredoxin	Rahnella aquatilis
WP_126124983.1	phenylalanine--tRNA ligase subunit beta	Rahnella aquatilis
WP_126124992.1	MBL fold metallo-hydrolase	Rahnella aquatilis
WP_126125055.1	2-oxoglutarate dehydrogenase complex dihydrolipoyllysine-residue succinyltransferase	Rahnella aquatilis
WP_126125223.1	2-iminobutanoate/2-iminopropanoate deaminase	Rahnella aquatilis
WP_126125249.1	glycerol-3-phosphate 1-O-acyltransferase PlsB	Rahnella aquatilis
WP_126125280.1	phosphate ABC transporter substrate-binding protein PstS	Rahnella aquatilis
WP_131637279.1	porin OmpC	Rahnella aquatilis
WP_131637507.1	type VI secretion system ATPase TssH	Rahnella aquatilis
WP_131637863.1	NAD-dependent epimerase	Rahnella aquatilis
WP_131637894.1	galactonate dehydratase	Rahnella aquatilis
WP_131638012.1	phage tail sheath protein	Rahnella aquatilis
WP_131638044.1	ribonuclease E	Rahnella aquatilis
WP_131638158.1	4-hydroxy-tetrahydrodipicolinate synthase	Rahnella aquatilis
WP_131638170.1	molecular chaperone HtpG	Rahnella aquatilis
WP_131638206.1	phosphoribosylformylglycinamidine synthase	Rahnella aquatilis
WP_131638346.1	glutamate-1-semialdehyde 2,1-aminomutase	Rahnella aquatilis
WP_131638362.1	GMP reductase	Rahnella aquatilis
WP_131638366.1	glutamine-hydrolyzing carbamoyl-phosphate synthase small subunit	Rahnella aquatilis
WP_131638370.1	molecular chaperone DnaK	Rahnella aquatilis
WP_131638459.1	RNA-binding transcriptional accessory protein	Rahnella aquatilis
WP_131638465.1	glycerol-3-phosphate dehydrogenase	Rahnella aquatilis
WP_131638510.1	heat shock chaperone IbpB	Rahnella aquatilis
WP_131638522.1	guanylate kinase	Rahnella aquatilis
WP_131638540.1	glycine C-acetyltransferase	Rahnella aquatilis

Protein code	Protein identification	Reference organism
WP_131638567.1	dipeptidase PepE	Rahnella aquatilis
WP_131638597.1	phospholipid-binding protein MlaC	Rahnella aquatilis
WP_131638630.1	NADPH-dependent assimilatory sulfite reductase flavoprotein subunit	Rahnella aquatilis
WP_131638703.1	alanine transaminase	Rahnella aquatilis
WP_131638762.1	S-(hydroxymethyl)glutathione dehydrogenase/class III alcohol dehydrogenase	Rahnella aquatilis
WP_131638766.1	ATP-dependent Clp protease ATP-binding subunit ClpA	Rahnella aquatilis
WP_147243271.1	MULTISPECIES: ribosome maturation factor RimP	Rahnella sp.
WP_147423484.1	methionine--tRNA ligase	Rahnella aquatilis
WP_148271887.1	leucyl aminopeptidase	Rahnella aquatilis

Table S2. Identified proteins in Sp2 (*Serratia plymuthica*).

Protein code	Protein identification	Reference organism
AGO53372.1	Phosphoglucosamine mutase GlmM	Serratia plymuthica 4Rx13
AGO53866.1	Transporter	Serratia plymuthica 4Rx13
AGO53983.1	YbbN	Serratia plymuthica 4Rx13
AGO54427.1	Methionine--tRNA ligase MetG	Serratia plymuthica 4Rx13
AGO54965.1	Phosphoenolpyruvate synthase PpsA	Serratia plymuthica 4Rx13
AGO54981.1	Riboflavin synthase rRbE	Serratia plymuthica 4Rx13
AGO55780.1	Methyl-accepting chemotaxis serine transducer	Serratia plymuthica 4Rx13
AGO55878.1	Alpha-galactosidase MelA	Serratia plymuthica 4Rx13
AGO57515.1	Vitamin B12 transporter BtuB	Serratia plymuthica 4Rx13
AGP46727.1	Hypothetical protein M621_02115	Serratia plymuthica S13
AHY05214.1	Hypothetical protein sch_01130	Serratia plymuthica
AHY05458.1	Hypothetical protein sch_02450	Serratia plymuthica
AHY05506.1	Hypothetical protein sch_02715	Serratia plymuthica
AHY05780.1	Tail sheath protein	Serratia plymuthica
AHY05781.1	Tail assembly protein	Serratia plymuthica
AHY05782.1	Phage tail protein	Serratia plymuthica
AHY05833.1	Ligand-gated channel protein	Serratia plymuthica
AHY05874.1	Glycerophosphodiester phosphodiesterase	Serratia plymuthica
AHY06324.1	Succinate-semialdehyde dehydrogenase	Serratia plymuthica
AHY06546.1	Phosphomannomutase	Serratia plymuthica
AHY06610.1	Lipoprotein	Serratia plymuthica
AHY06682.1	Aminopeptidase N	Serratia plymuthica
AHY07005.1	Hypothetical protein sch_10780	Serratia plymuthica
AHY07155.1	ABC transporter substrate-binding protein	Serratia plymuthica
AHY07389.1	Entry exclusion protein 2	Serratia plymuthica
AHY08628.1	Nitrate reductase	Serratia plymuthica
AHY08795.1	NAD synthetase	Serratia plymuthica
AHY09250.1	Filamentous hemagglutinin	Serratia plymuthica
AHY09484.1	Membrane protein	Serratia plymuthica
AHY09490.1	Hemolysin	Serratia plymuthica
AHY09911.1	Hypothetical protein sch_10595	Serratia plymuthica
ANJ91604.1	Chitin-binding protein	Serratia plymuthica
ANJ92775.1	Signal recognition particle	Serratia plymuthica
ANJ93567.1	Hypothetical protein ADP72_11505	Serratia plymuthica
ANJ96163.1	Hypothetical protein ADP72_25480	Serratia plymuthica
AYG77889.1	RNA polymerase II subunit, partial	Alternaria tenuissima
KYG15170.1	Filamentous hemagglutinin	Serratia plymuthica
OJT42892.1	Hypothetical protein BSR04_08075	Serratia plymuthica
pdb	3GBE	
RMN16896.1	Hypothetical protein ALQ63_00259	Serratia plymuthica
RMN17212.1	Hypothetical protein ALQ63_01830	Serratia plymuthica
RMN17366.1	Hypothetical protein ALQ63_02794	Serratia plymuthica
RMN18076.1	Hypothetical protein ALQ63_02431	Serratia plymuthica
RMN18164.1	Hypothetical protein ALQ63_00091	Serratia plymuthica
RMN18602.1	Hypothetical protein ALQ63_02924	Serratia plymuthica
RMN18940.1	Hypothetical protein ALQ63_04066	Serratia plymuthica
RMN19543.1	Hypothetical protein ALQ63_00888	Serratia plymuthica
RMN19950.1	Hypothetical protein ALQ63_04057	Serratia plymuthica
RMN20703.1	Hypothetical protein ALQ63_01148	Serratia plymuthica
RMN20967.1	Hypothetical protein ALQ63_00124	Serratia plymuthica
SQI30255.1	ATP-dependent zinc metalloprotease FtsH	Serratia plymuthica
SQI33180.1	Acriflavine resistance protein A precursor	Serratia plymuthica
SQI34124.1	Phosphate starvation-inducible protein psiF precursor	Serratia plymuthica
SQI39343.1	Chitinase D precursor	Serratia plymuthica
SQI45366.1	PTS-dependent dihydroxyacetone kinase, dihydroxyacetone-binding subunit DhaK	Serratia plymuthica
SQI46070.1	30s ribosomal protein S3	Serratia plymuthica
VEA62507.1	Aspartokinase I/homoserine dehydrogenase I	Serratia plymuthica
VEA67671.1	Pyruvate dehydrogenase E1 component	Serratia plymuthica
VEA68775.1	Ornithine/acetylornithine aminotransferase	Serratia plymuthica
VEI18669.1	Uncharacterised protein	Serratia plymuthica

Protein code	Protein identification	Reference organism
WP_001181005.1	30S ribosomal protein S10	Gammaproteobacteria
WP_002210315.1	Transcription antiterminator/RNA stability regulator CspE	Yersiniaceae
WP_004090925.1	cAMP-activated global transcriptional regulator CRP	Enterobacterales
WP_004093983.1	50S ribosomal protein L34	Enterobacterales
WP_004391482.1	Shikimate kinase AroK	Enterobacterales
WP_004709363.1	Two-component system response regulator OmpR	Enterobacterales
WP_004719003.1	Acyl carrier protein	Enterobacterales
WP_004847623.1	Nitrogen regulatory protein P-II	Enterobacterales
WP_004928318.1	Leucine-responsive transcriptional regulator Lrp	Enterobacterales
WP_004929726.1	50S ribosomal protein L17	Serratia sp.
WP_004929779.1	50S ribosomal protein L4	Enterobacterales
WP_004930426.1	30S ribosomal protein S12	Enterobacterales
WP_004932501.1	30S ribosomal protein S16	Enterobacterales
WP_004933502.1	30s ribosomal protein s15	Proteobacteria
WP_004933536.1	Ribosome assembly RNA-binding protein YhbY	Enterobacterales
WP_004933559.1	50S ribosomal protein L27	Enterobacterales
WP_004933561.1	50S ribosomal protein L21	Enterobacterales
WP_004941818.1	2,3-diphosphoglycerate-dependent phosphoglycerate mutase	Serratia sp.
WP_004942247.1	Outer membrane protein OmpX	Serratia sp.
WP_004942332.1	Glutathione ABC transporter substrate-binding protein GsiB	Serratia plymuthica
WP_004942358.1	NAD-dependent malic enzyme	Serratia sp.
WP_004942530.1	Arginine ABC transporter substrate-binding protein	Serratia sp.
WP_004942542.1	Heavy metal-binding domain-containing protein	Serratia sp.
WP_004942608.1	Thioredoxin-disulfide reductase	Serratia sp.
WP_004942614.1	Outer membrane lipoprotein chaperone LolA	Serratia sp.
WP_004942617.1	Serine--tRNA ligase	Serratia sp.
WP_004942650.1	30S ribosomal protein S1	Serratia sp.
WP_004942697.1	L,D-transpeptidase	Serratia sp.
WP_004942906.1	Universal stress protein	Serratia sp.
WP_004943006.1	Hypothetical protein	Serratia sp.
WP_004943016.1	Ycei family protein	Serratia sp.
WP_004943122.1	CopC domain-containing protein YobA	Serratia sp.
WP_004943136.1	Trans-2-enoyl-CoA reductase family protein	Serratia sp.
WP_004943172.1	Hypothetical protein	Serratia plymuthica
WP_004943364.1	SDR family oxidoreductase	Serratia sp.
WP_004943591.1	50s ribosomal protein l35	Serratia sp.
WP_004943595.1	50S ribosomal protein L20	Serratia sp.
WP_004943598.1	Phenylalanine--tRNA ligase subunit alpha	Serratia sp.
WP_004943604.1	Integration host factor subunit alpha	Yersiniaceae
WP_004943717.1	Major outer membrane lipoprotein	Serratia sp.
WP_004943789.1	Transcriptional regulator SlyA	Serratia sp.
WP_004943793.1	Glycine zipper 2TM domain-containing protein	Serratia sp.
WP_004944077.1	Alpha-glucosidase	Serratia plymuthica
WP_004944994.1	DUF1471 domain-containing protein	Serratia plymuthica
WP_004945038.1	2-hydroxyacid dehydrogenase	Serratia sp.
WP_004945089.1	Thiol peroxidase	Serratia sp.
WP_004945257.1	BON domain-containing protein	Serratia sp.
WP_004945391.1	Glyceraldehyde-3-phosphate dehydrogenase	Serratia sp.
WP_004945472.1	Cell division topological specificity factor MinE	Serratia sp.
WP_004945505.1	Pyruvate kinase	Serratia plymuthica
WP_004945950.1	Flic/FljB family flagellin	Serratia plymuthica
WP_004945953.1	Flagellar filament capping protein fliD	Serratia sp.
WP_004946072.1	Flagellar hook-filament junction protein FlgL	Serratia sp.
WP_004946081.1	Flagellar basal-body rod protein FlgG	Serratia sp.
WP_004946278.1	Transcription antiterminator/RNA stability regulator CspE	Yersiniaceae
WP_004946879.1	DUF1440 domain-containing protein	Serratia sp.
WP_004947039.1	Fused PTS fructose transporter subunit IIa/HPr protein	Serratia plymuthica
WP_004947644.1	ABC transporter substrate-binding protein	Serratia plymuthica
WP_004947678.1	Cysteine synthase A	Serratia sp.
WP_004947686.1	PTS glucose transporter subunit IIa	Serratia sp.
WP_004947713.1	Sulfate ABC transporter substrate-binding protein	Serratia sp.
WP_004947785.1	M48 family metallopeptidase	Serratia sp.

Protein code	Protein identification	Reference organism
WP_004947911.1	Hypoxanthine phosphoribosyltransferase	Serratia sp.
WP_004947931.1	Bifunctional aconitate hydratase 2/2-methylisocitrate dehydratase	Serratia sp.
WP_004947949.1	Pyruvate dehydrogenase complex transcriptional repressor PdhR	Serratia sp.
WP_004948431.1	DUF883 domain-containing protein	Serratia sp.
WP_004948486.1	Stringent starvation protein A	Serratia sp.
WP_004948488.1	30S ribosomal protein S9	Serratia sp.
WP_004948491.1	50S ribosomal protein L13	Serratia sp.
WP_004948506.1	UDP-N-acetylglucosamine 1-carboxyvinyltransferase	Serratia sp.
WP_004948518.1	Phospholipid-binding protein MlaC	Serratia sp.
WP_004949058.1	D-sedoheptulose 7-phosphate isomerase	Serratia sp.
WP_004949189.1	Pyrimidine/purine nucleoside phosphorylase	Serratia sp.
WP_004949238.1	Peroxiredoxin C	Serratia sp.
WP_004949246.1	Preprotein translocase subunit YajC	Serratia sp.
WP_004949262.1	6,7-dimethyl-8-ribityllumazine synthase	Serratia sp.
WP_004949342.1	Hypothetical protein	Serratia sp.
WP_004949394.1	Bifunctional UDP-sugar hydrolase/5'-nucleotidase	Serratia sp.
WP_004949579.1	DUF493 family protein	Serratia sp.
WP_004949631.1	CNNM family magnesium/cobalt transport protein CorC	Serratia sp.
WP_004949671.1	Glutamine--tRNA ligase	Serratia sp.
WP_004949690.1	Flavodoxin FldA	Serratia sp.
WP_004949756.1	ADP-forming succinate--CoA ligase subunit beta	Serratia sp.
WP_004949773.1	Tol-Pal system protein TolB	Serratia sp.
WP_004949927.1	Purine-nucleoside phosphorylase	Serratia sp.
WP_004949970.1	OmpA family protein	Serratia sp.
WP_004949986.1	Protein CreA	Serratia plymuthica
WP_004949989.1	Two-component system response regulator ArcA	Serratia sp.
WP_004950026.1	Molecular chaperone DnaK	Serratia sp.
WP_004950148.1	Cell division protein FtsA	Serratia sp.
WP_004950216.1	CTP synthase (glutamine hydrolyzing)	Serratia sp.
WP_004950328.1	Carbon storage regulator CsrA	Serratia sp.
WP_004950372.1	50S ribosomal protein L19	Serratia sp.
WP_004950476.1	F0F1 ATP synthase subunit B	Serratia sp.
WP_004950485.1	F0F1 ATP synthase subunit gamma	Serratia sp.
WP_004950512.1	ABC transporter substrate-binding protein	Serratia sp.
WP_004950559.1	DNA topoisomerase (ATP-hydrolyzing) subunit B	Serratia sp.
WP_004950614.1	OmpA family lipoprotein	Serratia sp.
WP_004950637.1	DUF3053 domain-containing protein	Serratia sp.
WP_004950859.1	Insulinase family protein	Serratia sp.
WP_004950949.1	DUF1795 domain-containing protein	Serratia sp.
WP_004951125.1	30S ribosomal protein S4	Serratia sp.
WP_004951128.1	30S ribosomal protein S11	Serratia sp.
WP_004951130.1	30S ribosomal protein S13	Serratia sp.
WP_004951135.1	50S ribosomal protein L15	Serratia sp.
WP_004951140.1	30S ribosomal protein S5	Serratia sp.
WP_004951145.1	50S ribosomal protein L6	Serratia sp.
WP_004951149.1	30S ribosomal protein S8	Serratia sp.
WP_004951154.1	30S ribosomal protein S14	Serratia sp.
WP_004951155.1	50S ribosomal protein L5	Serratia sp.
WP_004951158.1	50S ribosomal protein L24	Serratia sp.
WP_004951161.1	50S ribosomal protein L14	Serratia sp.
WP_004951166.1	30S ribosomal protein S17	Serratia sp.
WP_004951169.1	50S ribosomal protein L29	Serratia sp.
WP_004951171.1	50S ribosomal protein L16	Serratia sp.
WP_004951174.1	50S ribosomal protein L22	Serratia sp.
WP_004951177.1	50S ribosomal protein L2	Serratia sp.
WP_004951179.1	50S ribosomal protein L23	Serratia sp.
WP_004951181.1	50S ribosomal protein L3	Serratia sp.
WP_004951196.1	Elongation factor G	Serratia sp.
WP_004951199.1	30S ribosomal protein S7	Serratia sp.
WP_004951488.1	Fimbrial protein	Serratia plymuthica
WP_004952064.1	Outer membrane protein assembly factor BamB	Serratia sp.
WP_004952077.1	Nucleoside-diphosphate kinase [yersiniaceae]	Yersiniaceae

Protein code	Protein identification	Reference organism
WP_004952090.1	Fe-S cluster assembly scaffold IscU	Yersiniaceae
WP_004952096.1	Fe-S cluster assembly transcriptional regulator IscR	Serratia sp.
WP_004952206.1	Acetyl-CoA carboxylase carboxyl transferase subunit alpha	Serratia plymuthica
WP_004952213.1	Molecular chaperone Skp	Serratia sp.
WP_004952223.1	UMP kinase	Serratia sp.
WP_004952224.1	Elongation factor Ts	Serratia sp.
WP_004952226.1	30S ribosomal protein S2	Serratia sp.
WP_004952287.1	YgdI/YgdR family lipoprotein	Serratia sp.
WP_004952361.1	Peptidoglycan DD-metalloendopeptidase family protein	Serratia sp.
WP_004952400.1	Lysine--tRNA ligase	Serratia sp.
WP_004952410.1	Bifunctional protein-disulfide isomerase/oxidoreductase DsbC	Serratia sp.
WP_004952521.1	Oxidative stress defense protein	Serratia sp.
WP_004952575.1	Class II fructose-bisphosphate aldolase	Serratia sp.
WP_004952634.1	Co-chaperone GroeS	Enterobacterales
WP_004952713.1	Protease modulator HflC	Serratia sp.
WP_004952751.1	50S ribosomal protein L9	Serratia sp.
WP_004952787.1	Inorganic diphosphatase	Serratia sp.
WP_004952791.1	Class 1 fructose-bisphosphatase	Serratia sp.
WP_004952830.1	Serine-type D-Ala-D-Ala carboxypeptidase	Serratia sp.
WP_004952832.1	Transcription elongation factor GreA	Serratia sp.
WP_004952855.1	Transcription termination/antitermination protein NusA	Serratia sp.
WP_004953176.1	50S ribosomal protein L31	Serratia sp.
WP_004953193.1	Septal ring assembly protein ZapB	Serratia sp.
WP_004953227.1	6-phosphofructokinase	Serratia sp.
WP_004953231.1	Periplasmic heavy metal sensor	Serratia sp.
WP_004953234.1	Envelope stress response regulator transcription factor CpxR	Serratia sp.
WP_004953342.1	50S ribosomal protein L28	Yersiniaceae
WP_004953385.1	DNA-directed RNA polymerase subunit omega	Serratia sp.
WP_004953623.1	Autonomous glycyl radical cofactor GrcA	Serratia sp.
WP_004953928.1	Transcription termination/antitermination protein NusG	Serratia sp.
WP_004953932.1	50S ribosomal protein L1	Serratia sp.
WP_006316755.1	3-deoxy-8-phosphooctulonate synthase	Serratia sp.
WP_006316879.1	Thioredoxin-dependent thiol peroxidase	Serratia sp.
WP_006316936.1	Aspartate--tRNA ligase	Serratia plymuthica
WP_006317091.1	30S ribosomal protein S9	Serratia sp.
WP_006317092.1	50S ribosomal protein L13	Serratia sp.
WP_006317094.1	DUF1043 family protein	Serratia sp.
WP_006317106.1	LPS export abc transporter ATP-binding protein	Serratia sp.
WP_006317219.1	Matrixin family metalloprotease	Serratia plymuthica
WP_006317459.1	30S ribosomal protein S6	Serratia sp.
WP_006317468.1	Peptidyl-prolyl cis-trans isomerase	Serratia sp.
WP_006317617.1	Cell division protein FtsZ	Serratia sp.
WP_006317741.1	Triose-phosphate isomerase	Serratia sp.
WP_006317766.1	Argininosuccinate synthase	Serratia plymuthica
WP_006318001.1	NADH-quinone oxidoreductase subunit C/D	Serratia plymuthica
WP_006318476.1	Autonomous glycyl radical cofactor GrcA	Serratia sp.
WP_006318638.1	MetQ/NlpA family lipoprotein	Serratia sp.
WP_006319070.1	Ribulose-phosphate 3-epimerase	Serratia sp.
WP_006319342.1	Hypothetical protein	Serratia plymuthica
WP_006319550.1	Thiol peroxidase	Serratia sp.
WP_006319787.1	ATP-dependent Clp endopeptidase proteolytic subunit ClpP	Serratia sp.
WP_006319788.1	ATP-dependent protease ATP-binding subunit ClpX	Serratia sp.
WP_006320726.1	Bifunctional 3-hydroxydecanoyl-ACP dehydratase/trans-2-decenoyl-ACP- isomerase	Serratia sp.
WP_006320904.1	Serine protease inhibitor ecotin	Serratia plymuthica
WP_006321188.1	DNA-binding transcriptional regulator H-NS	Serratia sp.
WP_006321294.1	Xanthine phosphoribosyltransferase	Serratia sp.
WP_006321446.1	Flagellar basal body rod protein flgc	Serratia sp.
WP_006321571.1	ATP-binding cassette domain-containing protein	Serratia plymuthica
WP_006321728.1	Glycine--tRNA ligase subunit alpha	Serratia sp.
WP_006321789.1	Phosphate ABC transporter substrate-binding protein PstS	Serratia sp.
WP_006321796.1	F0F1 ATP synthase subunit gamma	Serratia sp.
WP_006321797.1	F0F1 ATP synthase subunit alpha	Serratia sp.

Protein code	Protein identification	Reference organism
WP_006321798.1	F0F1 atp synthase subunit delta	Serratia sp.
WP_006322046.1	Elongation factor P	Serratia sp.
WP_006322050.1	DUF4156 domain-containing protein	Serratia sp.
WP_006322224.1	Hypothetical protein	Serratia plymuthica
WP_006322514.1	23S rRNA accumulation protein YceD	Serratia sp.
WP_006322834.1	Hypothetical protein, partial	Serratia sp.
WP_006322974.1	ABC transporter substrate-binding protein	Serratia plymuthica
WP_006323135.1	50S ribosomal protein L11	Serratia sp.
WP_006323136.1	50S ribosomal protein L10	Serratia sp.
WP_006323137.1	50S ribosomal protein L7/L12	Serratia sp.
WP_006323187.1	Entericidin A/B family lipoprotein	Serratia sp.
WP_006323248.1	Bifunctional 2',3'-cyclic-nucleotide 2'-phosphodiesterase/3'-nucleotidase	Serratia plymuthica
WP_006323429.1	L,D-transpeptidase family protein	Serratia sp.
WP_006323548.1	Isoleucine--tRNA ligase	Serratia sp.
WP_006323695.1	Phage major tail tube protein	Serratia sp.
WP_006323696.1	Phage tail sheath protein	Serratia plymuthica
WP_006323718.1	Outer membrane protein assembly factor BamD	Serratia sp.
WP_006323817.1	ABC transporter substrate-binding protein	Serratia sp.
WP_006323877.1	DUF1471 domain-containing protein	Serratia sp.
WP_006323924.1	6,7-dimethyl-8-ribityllumazine synthase	Serratia sp.
WP_006323936.1	DNA-binding protein HU-beta	Serratia sp.
WP_006324117.1	Tol-Pal system protein TolQ	Serratia sp.
WP_006324221.1	D-alanine--D-alanine ligase	Serratia sp.
WP_006324338.1	DNA starvation/stationary phase protection protein Dps	Serratia sp.
WP_006324437.1	NADP-dependent phosphogluconate dehydrogenase	Serratia plymuthica
WP_006324520.1	Arginine ABC transporter substrate-binding protein	Serratia plymuthica
WP_006324547.1	Formate C-acetyltransferase	Serratia sp.
WP_006324584.1	Porin OmpA	Serratia plymuthica
WP_006324721.1	Penicillin-binding protein activator LpoB	Serratia sp.
WP_006325002.1	DUF945 domain-containing protein	Serratia sp.
WP_006325067.1	Peptidyl-dipeptidase Dcp	Serratia plymuthica
WP_006325615.1	Hypothetical protein	Serratia sp.
WP_006325706.1	GTP cyclohydrolase II	Serratia plymuthica
WP_006325792.1	Bifunctional acetaldehyde-CoA/alcohol dehydrogenase	Serratia plymuthica
WP_006325948.1	Peptidoglycan-binding protein LysM	Serratia sp.
WP_006326731.1	Deoxyribonuclease IV	Serratia plymuthica
WP_006326771.1	Porin OmpC	Serratia plymuthica
WP_006326780.1	Ribonucleoside-diphosphate reductase subunit alpha	Serratia sp.
WP_006326854.1	NADH-quinone oxidoreductase subunit NuoG	Serratia sp.
WP_006327417.1	N-acetyltransferase	Serratia plymuthica
WP_006327784.1	Phosphoglycerate kinase	Serratia sp.
WP_006327880.1	Hypothetical protein	Serratia sp.
WP_006327884.1	Pyruvate dehydrogenase complex dihydrolipoyllysine-residue acetyltransferase	Serratia plymuthica
WP_006327890.1	Pyruvate dehydrogenase (acetyl-transferring), homodimeric type	Serratia plymuthica
WP_006327903.1	Pyrroline-5-carboxylate reductase	Serratia plymuthica
WP_006328168.1	Outer membrane channel protein TolC	Serratia plymuthica
WP_006328270.1	Lipopolysaccharide ABC transporter substrate-binding protein LptA	Serratia sp.
WP_006328507.1	Peptidylprolyl isomerase a	Serratia sp.
WP_006328854.1	Sulfate ABC transporter substrate-binding protein	Serratia plymuthica
WP_006328929.1	YicC family protein	Serratia sp.
WP_006329006.1	Aspartate--ammonia ligase	Serratia sp.
WP_012004757.1	DNA-binding protein HU-alpha	Serratia sp.
WP_012006065.1	Deoxyribose-phosphate aldolase	Serratia sp.
WP_013810959.1	HAMP domain-containing protein	Serratia sp.
WP_013811104.1	Bifunctional demethylmenaquinone methyltransferase/2-methoxy-6-polyprenyl-1,4-benzoquinol methylase UbiE	Serratia sp.
WP_013811124.1	Elongation factor Tu	Serratia sp.
WP_013811127.1	DNA-directed RNA polymerase subunit beta'	Serratia sp.
WP_013811241.1	Protease modulator HflC	Serratia sp.
WP_013811258.1	YtfJ family protein	Serratia sp.
WP_013811266.1	Malate dehydrogenase	Serratia sp.
WP_013811278.1	Polyribonucleotide nucleotidyltransferase	Serratia sp.

Protein code	Protein identification	Reference organism
WP_013811321.1	2-iminobutanoate/2-iminopropanoate deaminase	Serratia sp.
WP_013811442.1	Transaldolase	Serratia sp.
WP_013811470.1	Peptidylprolyl isomerase SurA	Serratia sp.
WP_013811512.1	Glutamate-1-semialdehyde 2,1-aminomutase	Serratia sp.
WP_013811522.1	Phosphopyruvate hydratase	Serratia sp.
WP_013811550.1	S-ribosylhomocysteine lyase	Serratia sp.
WP_013811555.1	Phage tail tape measure protein	Serratia sp.
WP_013811573.1	Ribosome-associated translation inhibitor RaiA	Serratia sp.
WP_013811790.1	Adenylate kinase	Serratia sp.
WP_013811875.1	LPS assembly lipoprotein LptE	Serratia sp.
WP_013811881.1	Amino acid ABC transporter substrate-binding protein	Serratia sp.
WP_013811900.1	Ferric iron uptake transcriptional regulator	Serratia sp.
WP_013811925.1	Cytochrome ubiquinol oxidase subunit I	Serratia sp.
WP_013811938.1	2,3-diphosphoglycerate-dependent phosphoglycerate mutase	Serratia sp.
WP_013812016.1	ABC transporter substrate-binding protein	Serratia sp.
WP_013812121.1	DNA starvation/stationary phase protection protein Dps	Serratia sp.
WP_013812141.1	Fimbria/pilus periplasmic chaperone	Serratia sp.
WP_013812178.1	Galactose/glucose ABC transporter substrate-binding protein MglB	Serratia sp.
WP_013812257.1	Arginine ABC transporter substrate-binding protein	Serratia sp.
WP_013812293.1	3-phosphoserine/phosphohydroxythreonine transaminase	Serratia sp.
WP_013812310.1	Aspartate/tyrosine/aromatic aminotransferase	Serratia sp.
WP_013812357.1	DNA/RNA non-specific endonuclease	Serratia sp.
WP_013812467.1	DUF2511 domain-containing protein	Serratia sp.
WP_013812491.1	Hypothetical protein	Serratia sp.
WP_013812632.1	Threonine--tRNA ligase	Serratia sp.
WP_013812633.1	Translation initiation factor IF-3	Serratia sp.
WP_013812961.1	Glu/Leu/Phe/Val dehydrogenase	Serratia sp.
WP_013813138.1	YciK family oxidoreductase	Serratia sp.
WP_013813311.1	Septum site-determining protein MinD	Serratia sp.
WP_013813492.1	Flagellar hook protein FlgE	Serratia sp.
WP_013813653.1	Atp-grasp domain-containing protein	Serratia sp.
WP_013813723.1	DUF1440 domain-containing protein	Serratia sp.
WP_013813877.1	Stress response protein ElaB	Serratia sp.
WP_013813948.1	Phospholipid-binding lipoprotein MlaA	Serratia sp.
WP_013814033.1	Nitrate reductase catalytic subunit NapA	Serratia sp.
WP_013814057.1	Outer membrane protein assembly factor BamC	Serratia sp.
WP_013814058.1	4-hydroxy-tetrahydrodipicolinate synthase	Serratia sp.
WP_013814237.1	Glycine betaine/L-proline ABC transporter substrate-binding protein prox	Serratia sp.
WP_013814266.1	Outer membrane protein assembly factor BamA	Serratia sp.
WP_013814397.1	Glycine cleavage system aminomethyltransferase GcvT	Serratia sp.
WP_013814611.1	Cell division protein FtsP	Serratia sp.
WP_013814716.1	Acetyl-CoA carboxylase biotin carboxylase subunit	Serratia sp.
WP_013814788.1	Large-conductance mechanosensitive channel protein MscL	Serratia sp.
WP_013814793.1	50S ribosomal protein L30	Serratia sp.
WP_013814794.1	Bacterioferritin	Serratia sp.
WP_013814922.1	Oligopeptidase A	Serratia sp.
WP_013814996.1	HslU--HslV peptidase ATPase subunit	Serratia sp.
WP_013815010.1	2,3-bisphosphoglycerate-independent phosphoglycerate mutase	Serratia sp.
WP_013815013.1	Glycine C-acetyltransferase	Serratia sp.
WP_013815047.1	Glutamate--ammonia ligase	Serratia sp.
WP_020438512.1	Beta-Ala-His dipeptidase	Serratia plymuthica
WP_020438630.1	Asparagine synthase B	Serratia plymuthica
WP_020438738.1	Type 1 fimbrial protein	Serratia sp.
WP_020439242.1	FliC/FljB family flagellin	Serratia sp.
WP_020439536.1	Sulfate ABC transporter substrate-binding protein	Serratia plymuthica
WP_020439542.1	Transketolase	Serratia plymuthica
WP_020439668.1	Glycine betaine/L-proline ABC transporter ATP-binding protein ProV	Serratia plymuthica
WP_020439672.1	Rcs stress response system protein RcsF	Serratia sp.
WP_020439674.1	Envelope stress response activation lipoprotein NlpE	Serratia sp.
WP_020439835.1	Bifunctional D-glycero-beta-D-manno-heptose-7-phosphate kinase/D-glycero-beta-D-manno-heptose 1-phosphate adenylyltransferase HldE	Serratia sp.
WP_020439912.1	50S ribosomal protein L30	Serratia sp.

Protein code	Protein identification	Reference organism
WP_020439923.1	Tryptophan--tRNA ligase	Serratia plymuthica
WP_020453915.1	Uracil phosphoribosyltransferase	Serratia plymuthica
WP_020825678.1	Succinate--CoA ligase subunit alpha	Serratia sp.
WP_020837234.1	50S ribosomal protein L18	Serratia sp.
WP_020837240.1	50S ribosomal protein L14	Serratia sp.
WP_024484477.1	Phosphocarrier protein Hpr	Serratia sp.
WP_024912205.1	50s ribosomal protein l2	Yersiniaceae
WP_032693575.1	ABC transporter permease	Enterobacterales
WP_037425599.1	6-phosphofructokinase	Serratia sp.
WP_037429949.1	Hypothetical protein	Serratia plymuthica
WP_037430486.1	Type 1 fimbrial protein	Serratia plymuthica
WP_037431412.1	Phosphate acetyltransferase	Serratia plymuthica
WP_037431691.1	Biosynthetic arginine decarboxylase	Serratia plymuthica
WP_037432055.1	Uroporphyrinogen-III C-methyltransferase	Serratia plymuthica
WP_037432192.1	Phage major tail tube protein	Serratia plymuthica
WP_037432304.1	Helix-turn-helix domain-containing protein	Serratia plymuthica
WP_037432390.1	Molybdate ABC transporter substrate-binding protein	Serratia plymuthica
WP_037432618.1	Asparagine--tRNA ligase	Serratia plymuthica
WP_037433008.1	Outer membrane protein OmpW	Serratia plymuthica
WP_037433374.1	GSH-dependent disulfide bond oxidoreductase	Serratia plymuthica
WP_037433529.1	Inositol-1-monophosphatase	Serratia sp.
WP_037433563.1	NADP(H)-dependent aldo-keto reductase	Serratia plymuthica
WP_037433725.1	Glucose-6-phosphate isomerase	Serratia sp.
WP_041415851.1	ATP-dependent zinc metalloprotease FtsH	Serratia sp.
WP_041416326.1	Ribosome recycling factor	Serratia sp.
WP_041416570.1	Bifunctional UDP-4-amino-4-deoxy-l-arabinose formyltransferase/UDP-glucuronic acid oxidase AnA	Serratia sp.
WP_041416879.1	Ribonuclease E	Serratia plymuthica
WP_041417326.1	Methionine ABC transporter substrate-binding protein	Serratia plymuthica
WP_041417366.1	Hypothetical protein	Serratia plymuthica
WP_041417724.1	Hypothetical protein	Serratia sp.
WP_043912776.1	dUTP diphosphatase	Serratia sp.
WP_043912966.1	Ferrichrome porin FhuA	Serratia plymuthica
WP_043912969.1	Bifunctional indole-3-glycerol-phosphate synthase TrpC/phosphoribosylanthranilate isomerase TrpF	Serratia plymuthica
WP_043913104.1	Diaminobutyrate--2-oxoglutarate transaminase	Serratia sp.
WP_043913327.1	YceI family protein	Serratia plymuthica
WP_044554947.1	50S ribosomal protein L22	Serratia sp.
WP_052028166.1	Peptide ABC transporter substrate-binding protein	Serratia plymuthica
WP_062790903.1	ABC transporter substrate-binding protein	Serratia sp.
WP_062791335.1	Murein hydrolase activator NlpD	Serratia plymuthica
WP_062791847.1	UDP-glucose/gGDP-mannose dehydrogenase family protein	Serratia sp.
WP_062791875.1	Elongation factor Tu, partial	Serratia sp.
WP_062792110.1	Acetate kinase	Serratia sp.
WP_062792216.1	NAD(P)H-dependent oxidoreductase	Serratia sp.
WP_062792956.1	Spermidine/putrescine ABC transporter substrate-binding protein PotF	Serratia sp.
WP_062793098.1	Peptidylprolyl isomerase B	Serratia sp.
WP_062868083.1	Porin	Serratia plymuthica
WP_062868132.1	Beta-ketoacyl-ACP synthase I	Serratia sp.
WP_062868304.1	Phosphoribosylformylglycinamidine synthase	Serratia plymuthica
WP_062868384.1	Thymidylate synthase	Serratia plymuthica
WP_062868532.1	Phosphoglycerate dehydrogenase	Serratia plymuthica
WP_062868575.1	Agmatinase	Serratia plymuthica
WP_062868807.1	DUF883 domain-containing protein	Serratia plymuthica
WP_062868870.1	Transporter substrate-binding domain-containing protein	Serratia plymuthica
WP_062868872.1	Quinone oxidoreductase	Serratia plymuthica
WP_062868886.1	Lysine-sensitive aspartokinase 3	Serratia plymuthica
WP_062868899.1	L-threonylcarbamoyladenylate synthase type 1 TsaC	Serratia plymuthica
WP_062868906.1	DNA+B74-directed RNA polymerase subunit alpha	Enterobacterales
WP_062868943.1	Glycerol-3-phosphate dehydrogenase	Serratia plymuthica
WP_062869022.1	Atp-independent periplasmic protein-refolding chaperone	Serratia plymuthica
WP_062869058.1	Murein hydrolase activator EnvC	Serratia plymuthica

Protein code	Protein identification	Reference organism
WP_062869060.1	ADP-glyceromanno-heptose 6-epimerase	Serratia plymuthica
WP_062869220.1	Glycerophosphodiester phosphodiesterase	Serratia plymuthica
WP_062869242.1	5-methyltetrahydropteroyltriglutamate--homocysteine S-methyltransferase	Serratia plymuthica
WP_062869375.1	Alpha,alpha-phosphotrehalase	Serratia plymuthica
WP_062869593.1	Molecular chaperone DnaJ	Serratia plymuthica
WP_062869629.1	Cell division protein FtsZ	Serratia plymuthica
WP_062869659.1	Assimilatory sulfite reductase (NADPH) hemoprotein subunit	Serratia plymuthica
WP_062869793.1	DUF2501 domain-containing protein	Serratia plymuthica
WP_062869846.1	Trigger factor	Serratia plymuthica
WP_062869945.1	Citrate synthase	Serratia plymuthica
WP_062869947.1	Bifunctional metallophosphatase/5'-nucleotidase	Serratia plymuthica
WP_062869988.1	Hypothetical protein	Serratia plymuthica
WP_062870112.1	ABC transporter substrate-binding protein	Serratia plymuthica
WP_062870323.1	NADP-dependent phosphogluconate dehydrogenase	Serratia plymuthica
WP_062870397.1	Porin OmpC	Serratia plymuthica
WP_062870486.1	Metal-dependent hydrolase	Serratia plymuthica
WP_062870514.1	Hypothetical protein	Serratia plymuthica
WP_062870578.1	Hypothetical protein	Serratia plymuthica
WP_062870646.1	Pyruvate kinase PykF	Serratia plymuthica
WP_062870706.1	Class C beta-lactamase	Serratia plymuthica
WP_062870776.1	LLM class flavin-dependent oxidoreductase	Serratia plymuthica
WP_062871046.1	MipA/OmpV family protein	Serratia plymuthica
WP_063177426.1	Acetolactate synthase AlsS	Serratia plymuthica
WP_063177448.1	Aconitate hydratase AcnA	Serratia plymuthica
WP_063196854.1	Osmotically-inducible lipoprotein OsmE	Serratia plymuthica
WP_063196881.1	PTS fructose transporter subunit IIBC	Serratia plymuthica
WP_063197154.1	Hypothetical protein	Serratia plymuthica
WP_063197869.1	Phosphatase	Serratia plymuthica
WP_063197910.1	LPS assembly lipoprotein LptE	Serratia plymuthica
WP_063198069.1	Endopeptidase La	Serratia plymuthica
WP_063198203.1	Hypothetical protein	Serratia plymuthica
WP_063198541.1	Class II fumarate hydratase	Serratia plymuthica
WP_063198636.1	Energy-dependent translational throttle protein EttA	Serratia plymuthica
WP_063198645.1	Murein transglycosylase	Serratia plymuthica
WP_063198674.1	Co2+/Mg2+ efflux protein ApaG	Serratia plymuthica
WP_063198800.1	Baseplate assembly protein	Serratia plymuthica
WP_063198805.1	Phage baseplate assembly protein V	Serratia plymuthica
WP_063198834.1	F0F1 ATP synthase subunit beta	Serratia plymuthica
WP_063198840.1	Glutamine--fructose-6-phosphate transaminase (isomerizing)	Serratia plymuthica
WP_063199187.1	Dihydroorotase	Serratia plymuthica
WP_063199821.1	Serine protease inhibitor ecotin	Serratia plymuthica
WP_063199886.1	UDP-glucose 4-epimerase GalE	Serratia plymuthica
WP_063200017.1	Fimbrial biogenesis outer membrane usher protein	Serratia plymuthica
WP_063200032.1	Betaine-aldehyde dehydrogenase	Serratia plymuthica
WP_063200275.1	Pitrilysin	Serratia plymuthica
WP_063200407.1	PfaD family polyunsaturated fatty acid/polyketide biosynthesis protein	Serratia plymuthica
WP_063200412.1	Amidohydrolase	Serratia plymuthica
WP_063200622.1	Phosphoenolpyruvate carboxykinase (ATP)	Serratia plymuthica
WP_063200869.1	Glycerol dehydrogenase	Serratia plymuthica
WP_063201108.1	Oligopeptide ABC transporter substrate-binding protein OppA	Serratia plymuthica
WP_063201285.1	Endonuclease	Serratia plymuthica
WP_063201305.1	Long-chain fatty acid transporter FadL	Serratia plymuthica
WP_063201373.1	Glutamate--tRNA ligase	Serratia plymuthica
WP_063201803.1	Hypothetical protein	Serratia plymuthica
WP_063201917.1	Cystine ABC transporter substrate-binding protein	Serratia plymuthica
WP_063201935.1	Iron uptake system protein EfeO	Serratia plymuthica
WP_063202579.1	DUF1795 domain-containing protein	Serratia plymuthica
WP_063202720.1	Peptidoglycan-binding protein LysM	Serratia plymuthica
WP_063202862.1	L-asparaginase 2, partial	Serratia plymuthica
WP_063203027.1	DUF1481 domain-containing protein	Serratia plymuthica
WP_063203045.1	DNA-directed RNA polymerase subunit beta'	Serratia plymuthica
WP_063203173.1	FKBP-type peptidyl-prolyl cis-trans isomerase	Serratia plymuthica

Protein code	Protein identification	Reference organism
WP_064798650.1	Two-component system response regulator ArcA	Serratia sp.
WP_064798662.1	Alanine--tRNA ligase	Serratia plymuthica
WP_064798698.1	3',5'-cyclic-nucleotide phosphodiesterase	Serratia plymuthica
WP_064798746.1	UTP--glucose-1-phosphate uridylyltransferase GalU	Serratia plymuthica
WP_064798747.1	Polysaccharide export protein	Serratia plymuthica
WP_064798756.1	Hypothetical protein	Serratia plymuthica
WP_064798761.1	Phosphomannomutase CpsG	Serratia plymuthica
WP_064798779.1	Hypothetical protein	Serratia plymuthica
WP_064798816.1	Aldehyde dehydrogenase	Serratia plymuthica
WP_064798855.1	Hypothetical protein	Serratia plymuthica
WP_064798864.1	D-hexose-6-phosphate mutarotase	Serratia plymuthica
WP_064798956.1	Flagellar basal body rod protein FlgF	Serratia plymuthica
WP_064799070.1	Lytic murein transglycosylase B	Serratia plymuthica
WP_064799075.1	NADP-dependent oxaloacetate-decarboxylating malate dehydrogenase	Serratia plymuthica
WP_064799080.1	Nitrate reductase catalytic subunit NapA	Serratia plymuthica
WP_064799183.1	Non-ribosomal peptide synthetase	Serratia plymuthica
WP_064799229.1	Glutathione-disulfide reductase	Serratia plymuthica
WP_064799257.1	Tyrosine-protein kinase Wzc	Serratia plymuthica
WP_064799364.1	Lipoprotein localization protein LolB	Serratia plymuthica
WP_064799697.1	Bifunctional glycosyl transferase/transpeptidase	Serratia plymuthica
WP_065505698.1	Thioredoxin TrxA	Serratia sp.
WP_065505744.1	DNA-directed RNA polymerase subunit beta	Serratia sp.
WP_065505781.1	Polyribonucleotide nucleotidyltransferase	Serratia sp.
WP_065505783.1	DEAD/DEAH family ATP-dependent RNA helicase	Serratia sp.
WP_065505885.1	Glutamate--cysteine ligase	Serratia sp.
WP_065505898.1	Phage tail protein I	Serratia plymuthica
WP_065505914.1	23S rRNA pseudouridine(1911/1915/1917) synthase RluD	Serratia sp.
WP_065505963.1	Succinate dehydrogenase iron-sulfur subunit	Serratia sp.
WP_065505965.1	2-oxoglutarate dehydrogenase complex dihydrolipoyllysine-residue succinyltransferase	Serratia sp.
WP_065505971.1	Cell division protein CpoB	Serratia sp.
WP_065506192.1	Glycine zipper 2TM domain-containing protein	Serratia sp.
WP_065506275.1	Peptidyl-dipeptidase Dcp	Serratia plymuthica
WP_065506321.1	Pyruvate kinase	Serratia sp.
WP_065506399.1	Penicillin-insensitive murein endopeptidase	Serratia sp.
WP_065506404.1	Acetyl-CoA C-acyltransferase FadI	Serratia sp.
WP_065506483.1	Serine hydroxymethyltransferase	Serratia sp.
WP_065506613.1	Formate dehydrogenase subunit alpha	Serratia sp.
WP_065506637.1	Divisome-associated lipoprotein YraP	Serratia sp.
WP_065506702.1	Peptide deformylase	Serratia sp.
WP_065506761.1	Molybdopterin-dependent oxidoreductase	Serratia plymuthica
WP_071531447.1	Ribose-phosphate pyrophosphokinase	Serratia plymuthica
WP_071825589.1	S-type pyocin domain-containing protein	Serratia plymuthica
WP_073439042.1	NADH:flavin oxidoreductase/NADH oxidase	Serratia plymuthica
WP_073439169.1	Carboxylating nicotinate-nucleotide diphosphorylase	Serratia plymuthica
WP_073439268.1	PLP-dependent transferase	Serratia plymuthica
WP_073439293.1	Flagellar hook assembly protein FlgD	Serratia plymuthica
WP_073439433.1	Alcohol dehydrogenase AdhP	Serratia plymuthica
WP_073439450.1	Lipoprotein	Serratia plymuthica
WP_073439459.1	ABC transporter substrate-binding protein	Serratia plymuthica
WP_073439488.1	Adenine phosphoribosyltransferase	Serratia plymuthica
WP_073439559.1	M48 family metallopeptidase	Serratia plymuthica
WP_073439566.1	Peptidylprolyl isomerase	Serratia plymuthica
WP_073439704.1	Hypothetical protein	Serratia plymuthica
WP_073439740.1	Antibiotic biosynthesis monooxygenase	Serratia plymuthica
WP_073439779.1	DUF1496 domain-containing protein	Serratia plymuthica
WP_073439813.1	Aldose 1-epimerase family protein	Serratia plymuthica
WP_073439860.1	Carboxy terminal-processing peptidase	Serratia plymuthica
WP_073439872.1	Patatin-like phospholipase family protein	Serratia plymuthica
WP_073440018.1	Beta-glucosidase BglX	Serratia plymuthica
WP_073440037.1	Colicin-B	Serratia plymuthica
WP_073440047.1	Ribonucleotide-diphosphate reductase subunit beta	Serratia plymuthica

Protein code	Protein identification	Reference organism
WP_073440048.1	Catalase	Serratia plymuthica
WP_073440058.1	Metal ABC transporter substrate-binding protein	Serratia plymuthica
WP_073440102.1	Nucleotide exchange factor GrpE	Serratia plymuthica
WP_073440120.1	NAD-dependent epimerase/dehydratase family protein	Serratia plymuthica
WP_073440145.1	Adenylosuccinate lyase	Serratia plymuthica
WP_073440158.1	NAD-dependent succinate-semialdehyde dehydrogenase	Serratia plymuthica
WP_073440218.1	Thioredoxin domain-containing protein	Serratia plymuthica
WP_073440246.1	NADP-dependent oxaloacetate-decarboxylating malate dehydrogenase	Serratia plymuthica
WP_073440254.1	NAD-dependent DNA ligase LigA	Serratia plymuthica
WP_073440262.1	Aspartate-semialdehyde dehydrogenase	Serratia plymuthica
WP_073440264.1	P1 family peptidase	Serratia plymuthica
WP_073440272.1	GrxA family glutaredoxin	Serratia plymuthica
WP_073440294.1	Glu/Leu/Phe/val dehydrogenase	Serratia plymuthica
WP_073440327.1	Ribosome maturation factor RimP	Serratia sp.
WP_073440332.1	Glutathione ABC transporter substrate-binding protein GsiB	Serratia plymuthica
WP_073440375.1	Peptidase inhibitor I78 family protein	Serratia plymuthica
WP_073440417.1	ECA polysaccharide chain length modulation protein	Serratia plymuthica
WP_073440429.1	Alpha-D-glucose phosphate-specific phosphoglucomutase	Serratia plymuthica
WP_073440668.1	Glutamine-hydrolyzing carbamoyl-phosphate synthase small subunit	Serratia plymuthica
WP_073440736.1	Phosphoadenylyl-sulfate reductase	Serratia plymuthica
WP_073440737.1	NADPH-dependent assimilatory sulfite reductase flavoprotein subunit	Serratia plymuthica
WP_073440786.1	1-deoxy-D-xylulose-5-phosphate synthase	Serratia plymuthica
WP_073440800.1	Recombination-associated protein RdgC	Serratia plymuthica
WP_073440841.1	Glutamine-hydrolyzing GMP synthase	Serratia plymuthica
WP_073440858.1	DUF1090 domain-containing protein	Serratia plymuthica
WP_073440881.1	N-acetylmuramoyl-L-alanine amidase	Serratia plymuthica
WP_073440890.1	Fused PTS fructose transporter subunit IIa/HPr protein	Serratia plymuthica
WP_073440891.1	Trimethylamine N-oxide reductase catalytic subunit TorA	Serratia plymuthica
WP_073440955.1	Pyruvate dehydrogenase complex dihydrolipoyllysine-residue acetyltransferase	Serratia plymuthica
WP_073440965.1	Glutathione synthase	Serratia plymuthica
WP_073441180.1	Oxidoreductase	Serratia plymuthica
WP_073441184.1	Carbonic anhydrase	Serratia plymuthica
WP_073441200.1	Capsule assembly Wzi family protein	Serratia plymuthica
WP_073441219.1	N-acetylmuramoyl-L-alanine amidase	Serratia plymuthica
WP_073999785.1	Multifunctional acyl-CoA thioesterase I/protease I/lysophospholipase L1	Serratia plymuthica
WP_086012862.1	Molecular chaperone HtpG	Serratia plymuthica
WP_086012891.1	Aminopeptidase PepB	Serratia plymuthica
WP_086012895.1	Dihydrolipoyl dehydrogenase	Serratia sp.
WP_086016609.1	Glucans biosynthesis protein mdog	Serratia sp.
WP_110605874.1	Glucans biosynthesis protein MdoG	Serratia plymuthica
WP_110605936.1	Hypothetical protein	Serratia plymuthica
WP_111738404.1	Peptidoglycan glycosyltransferase FtsI	Serratia plymuthica
WP_111738524.1	Outer membrane channel protein TolC	Serratia plymuthica
WP_111738537.1	Bacterioferritin	Serratia plymuthica
WP_119802578.1	DUF2498 family protein	Serratia sp.
WP_119803235.1	NADP-dependent isocitrate dehydrogenase	Serratia sp.
WP_120978333.1	Transketolase	Serratia plymuthica
WP_120978363.1	Molecular chaperone HtpG	Serratia plymuthica
WP_120978375.1	Type 1 fimbrial protein	Serratia plymuthica
WP_122288565.1	Bifunctional glucose-1-phosphatase/inositol phosphatase	Serratia plymuthica
WP_122288636.1	YcjX family protein	Serratia plymuthica
WP_122288643.1	Heat shock protein HslJ	Serratia plymuthica
WP_122288665.1	Protein phosphatase CheZ	Serratia plymuthica
WP_122288670.1	Flagellar hook-associated protein FLgK	Serratia plymuthica
WP_122288686.1	Acid phosphatase	Serratia plymuthica
WP_122288733.1	Valine--tRNA ligase	Serratia plymuthica
WP_122288792.1	Small-conductance mechanosensitive channel MscS	Serratia plymuthica
WP_122288797.1	Xaa-Pro aminopeptidase	Serratia plymuthica
WP_122288801.1	Aminomethyl-transferring glycine dehydrogenase	Serratia plymuthica
WP_122288861.1	DUF3313 domain-containing protein	Serratia plymuthica
WP_122288872.1	S9 family peptidase	Serratia plymuthica
WP_122288873.1	4-hydroxy-tetrahydrodipicolinate synthase	Serratia plymuthica

Protein code	Protein identification	Reference organism
WP_122288887.1	Serine endoprotease DegQ	Serratia plymuthica
WP_122288888.1	Penicillin-binding protein activator	Serratia plymuthica
WP_122288902.1	Matrixin family metalloprotease	Serratia plymuthica
WP_122288941.1	Methionine synthase	Serratia plymuthica
WP_122288956.1	L,D-transpeptidase family protein	Serratia plymuthica
WP_122288989.1	Fe-S protein assembly chaperone HscA	Serratia plymuthica
WP_122289004.1	Glycoside hydrolase family 68 protein	Serratia plymuthica
WP_122289073.1	Multicopper oxidase CueO	Serratia plymuthica
WP_122289110.1	NAD(P)H nitroreductase	Serratia plymuthica
WP_122289113.1	Oligopeptide ABC transporter substrate-binding protein OppA	Serratia plymuthica
WP_122289146.1	Chitin-binding protein	Serratia plymuthica
WP_122289173.1	D-alanyl-D-alanine-carboxypeptidase/endopeptidase AmpH	Serratia plymuthica
WP_122289180.1	Phosphopentomutase	Serratia plymuthica
WP_122289187.1	LPS assembly protein LptD	Serratia plymuthica
WP_122289200.1	ABC transporter substrate-binding protein	Serratia plymuthica
WP_122289282.1	PTS N-acetyl glucosamine transporter subunit IIABC	Serratia plymuthica
WP_122289290.1	Endolytic peptidoglycan transglycosylase RlpA	Serratia plymuthica
WP_122289298.1	Family 20 glycosylhydrolase	Serratia plymuthica
WP_122289313.1	Proline--tRNA ligase	Serratia plymuthica
WP_122289361.1	Antirestriction protein	Serratia plymuthica
WP_122289388.1	Maltose/maltodextrin ABC transporter substrate-binding protein MalE	Serratia plymuthica
WP_122289463.1	Phenylalanine--tRNA ligase subunit beta	Serratia plymuthica
WP_122289546.1	Alanine--tRNA ligase	Serratia plymuthica
WP_122289575.1	Fe2+-enterobactin ABC transporter substrate-binding protein	Serratia plymuthica
WP_122289580.1	Cytidine deaminase	Serratia plymuthica
WP_122289584.1	Molybdopterin molybdotransferase MoeA	Serratia plymuthica
WP_122289609.1	Acetyl-CoA carboxylase, carboxyltransferase subunit beta	Serratia plymuthica
WP_122289614.1	NADH-quinone oxidoreductase subunit NuoF	Serratia plymuthica
WP_122289658.1	16S rRNA (cytosine(1402)-N(4))-methyltransferase RsmH	Serratia plymuthica
WP_122289663.1	Thiol:disulfide interchange protein DsbA	Serratia plymuthica
WP_122289706.1	Histidine phosphatase family protein	Serratia plymuthica
WP_122289728.1	Chaperonin GroEL	Serratia plymuthica
WP_122289729.1	Fumarate reductase (quinol) flavoprotein subunit	Serratia plymuthica
WP_122289796.1	DUF2974 domain-containing protein	Serratia plymuthica
WP_122289827.1	Xaa-Pro dipeptidase	Serratia plymuthica
WP_122289835.1	Matrixin family metalloprotease	Serratia plymuthica
WP_122289854.1	Redox-regulated ATPase YchF	Serratia plymuthica
WP_122290032.1	Peptidylprolyl isomerase a	Serratia plymuthica
WP_122290046.1	Hypothetical protein	Serratia plymuthica
WP_126479624.1	Transcription termination factor Rho	Serratia plymuthica
WP_126479690.1	Porphobilinogen synthase	Serratia plymuthica
WP_126479692.1	Dipeptidase PepE	Serratia plymuthica
WP_126479796.1	FtsH protease activity modulator HflK	Serratia plymuthica
WP_126479804.1	Ribonuclease R	Serratia plymuthica
WP_126479829.1	Outer membrane protein assembly factor	Serratia plymuthica
WP_126479856.1	Translation initiation factor IF-2	Serratia plymuthica
WP_126479860.1	DEAD/DEAH family ATP-dependent RNA helicase	Serratia plymuthica
WP_126480109.1	Thymidine phosphorylase	Serratia plymuthica
WP_126480153.1	Molecular chaperone DnaJ	Serratia plymuthica
WP_126480159.1	Isoleucine--trRNA ligase	Serratia plymuthica
WP_126480171.1	Glutamine-hydrolyzing carbamoyl-phosphate synthase small subunit	Serratia plymuthica
WP_126480223.1	Catabolite repressor/activator	Serratia plymuthica
WP_126480237.1	Undecaprenyldiphospho-muramoylpentapeptide beta-N-acetylglucosaminyltransferase	Serratia plymuthica
WP_126480247.1	Preprotein translocase subunit SecA	Serratia plymuthica
WP_126480265.1	Glutamate-1-semialdehyde 2,1-aminomutase	Serratia plymuthica
WP_126480329.1	Phosphoadenylyl-sulfate reductase	Serratia plymuthica
WP_126480368.1	Recombinase RecA	Serratia plymuthica
WP_126480386.1	DUF481 domain-containing protein	Serratia plymuthica
WP_126480399.1	ATP-dependent chaperone ClpB	Serratia plymuthica
WP_126480508.1	Na(+)-translocating NADH-quinone reductase subunit C	Serratia plymuthica
WP_126480520.1	Beta-Ala-His dipeptidase	Serratia plymuthica

Protein code	Protein identification	Reference organism
WP_126480584.1	DUF2076 domain-containing protein	Serratia plymuthica
WP_126480645.1	Gamma-glutamyltransferase [Serratia plymuthica]	Serratia plymuthica
WP_126480674.1	YajQ family cyclic di-GMP-binding protein	Serratia plymuthica
WP_126480684.1	Cytochrome o ubiquinol oxidase subunit II	Serratia plymuthica
WP_126480690.1	Endopeptidase La	Serratia plymuthica
WP_126480757.1	Multidrug efflux RND transporter periplasmic adaptor subunit SdeX	Serratia plymuthica
WP_126480797.1	Co-chaperone YbbN	Serratia plymuthica
WP_126480896.1	Glucosamine-6-phosphate deaminase	Serratia plymuthica
WP_126480952.1	Citrate synthase	Serratia plymuthica
WP_126480956.1	Succinate dehydrogenase flavoprotein subunit	Serratia plymuthica
WP_126480958.1	Succinate dehydrogenase iron-sulfur subunit	Serratia plymuthica
WP_126480972.1	Tol-Pal system protein TolB	Serratia plymuthica
WP_126480974.1	Cell division protein CpoB	Serratia plymuthica
WP_126481105.1	ABC transporter substrate-binding protein	Serratia plymuthica
WP_126481301.1	Glutamine ABC transporter substrate-binding protein GlnH	Serratia plymuthica
WP_126481381.1	ABC-f family ATPase	Serratia plymuthica
WP_126481398.1	S-formylglutathione hydrolase	Serratia plymuthica
WP_126481621.1	30S ribosomal protein S12 methylthiotransferase accessory protein YcaO	Serratia plymuthica
WP_126481625.1	3-phosphoserine/phosphohydroxythreonine transaminase	Serratia plymuthica
WP_126481629.1	(d)CMP kinase	Serratia plymuthica
WP_126481653.1	Aspartate/tyrosine/aromatic aminotransferase	Serratia plymuthica
WP_126481671.1	Quinone-dependent dihydroorotate dehydrogenase	Serratia plymuthica
WP_126481690.1	Porin OmpA	Serratia plymuthica
WP_126481850.1	Universal stress protein	Serratia plymuthica
WP_126481930.1	LuxR family transcriptional regulator	Serratia plymuthica
WP_126481956.1	ACP S-malonyltransferase	Serratia plymuthica
WP_126481958.1	3-oxoacyl-ACP reductase FabG	Serratia plymuthica
WP_126481970.1	PTS glucose transporter subunit IIbc	Serratia plymuthica
WP_126482134.1	Cupin domain-containing protein	Serratia plymuthica
WP_126482196.1	Nucleoside-specific channel-forming protein Tsx	Serratia plymuthica
WP_126482255.1	RNA chaperone ProQ	Serratia plymuthica
WP_126482297.1	Phenylalanine--tRNA ligase subunit beta	Serratia plymuthica
WP_126482406.1	Tyrosine--tRNA ligase	Serratia plymuthica
WP_126482500.1	DUF1283 family protein	Serratia plymuthica
WP_126482526.1	6-phospho-alpha-glucosidase	Serratia plymuthica
WP_126482598.1	Sugar ABC transporter substrate-binding protein	Serratia plymuthica
WP_126482879.1	Iron ABC transporter substrate-binding protein	Serratia plymuthica
WP_126482963.1	Serine protease	Serratia plymuthica
WP_126482993.1	Oligopeptide abc transporter substrate-binding protein OppA	Serratia plymuthica
WP_126483037.1	Exoribonuclease II	Serratia plymuthica
WP_126483066.1	Threonylcarbamoyl-AMP synthase	Serratia plymuthica
WP_126483126.1	Exodeoxyribonuclease III	Serratia plymuthica
WP_126483181.1	Fumarylacetoacetate hydrolase family protein	Serratia plymuthica
WP_126483185.1	Septum site-determining protein MinC	Serratia plymuthica
WP_126483203.1	Glucose-6-phosphate dehydrogenase	Serratia plymuthica
WP_126483215.1	YebC/PmpR family DNA-binding transcriptional regulator	Serratia plymuthica
WP_126483217.1	Aspartate--tRNA ligase	Serratia plymuthica
WP_126483270.1	PTS mannose transporter subunit IIAB	Serratia plymuthica
WP_126483286.1	Hypothetical protein	Serratia plymuthica
WP_126483290.1	Hypothetical protein	Serratia plymuthica
WP_126483508.1	NADP-dependent oxidoreductase	Serratia plymuthica
WP_126483916.1	Nitroreductase family protein	Serratia plymuthica
WP_126484047.1	1-phosphofructokinase	Serratia plymuthica
WP_126484117.1	DNA topoisomerase (ATP-hydrolyzing) subunit A	Serratia plymuthica
WP_126484181.1	Acetate kinase	Serratia plymuthica
WP_126484195.1	Histidine ABC transporter substrate-binding protein HisJ	Serratia plymuthica
WP_126484206.1	Acetyl-CoA carboxylase, carboxyltransferase subunit beta	Serratia plymuthica
WP_126484259.1	Acetyl-CoA C-acyltransferase FadI	Serratia plymuthica
WP_126484369.1	Phosphoenolpyruvate-protein phosphotransferase PtsI	Serratia plymuthica
WP_126484392.1	YgiW/YdeI family stress tolerance OB fold protein	Serratia plymuthica
WP_126484412.1	Transaldolase	Serratia plymuthica
WP_126484422.1	Nitrate reductase cytochrome c-type subunit	Serratia plymuthica

Protein code	Protein identification	Reference organism
WP_126484466.1	Phosphoribosylaminoimidazolesuccinocarboxamide synthase	Serratia plymuthica
WP_126484586.1	IMP dehydrogenase	Serratia plymuthica
WP_126484604.1	Outer membrane protein assembly factor BamB	Serratia plymuthica
WP_126484608.1	Histidine--tRNA ligase	Serratia plymuthica
WP_126484626.1	IscS subfamily cysteine desulfurase	Serratia plymuthica
WP_126484642.1	Two-component system QseEF-associated lipoprotein QseG	Serratia plymuthica
WP_126484735.1	DUF883 domain-containing protein	Serratia plymuthica
WP_126484810.1	Cytochrome c	Serratia plymuthica
WP_126484832.1	30S ribosomal protein S2	Serratia plymuthica
WP_126484836.1	2,3,4,5-tetrahydropyridine-2,6-dicarboxylate N-succinyltransferase	Serratia plymuthica
WP_126484840.1	Flavodoxin	Serratia plymuthica
WP_126484855.1	Murein transglycosylase A	Serratia plymuthica
WP_126484983.1	Cold-shock protein	Serratia plymuthica
WP_126484993.1	Lysine--tRNA ligase	Serratia plymuthica
WP_126485047.1	Aminomethyl-transferring glycine dehydrogenase	Serratia plymuthica
WP_126485049.1	Glycine cleavage system protein GcvH	Serratia plymuthica
WP_126485113.1	Methionine adenosyltransferase	Serratia plymuthica
WP_126485170.1	Fumarate hydratase	Serratia plymuthica
WP_126485184.1	XTP/dITP diphosphatase	Serratia plymuthica
WP_126485188.1	DUF2884 domain-containing protein	Serratia plymuthica
WP_126485323.1	Alcohol dehydrogenase	Serratia plymuthica
WP_126485325.1	2,5-didehydrogluconate reductase DkgA	Serratia plymuthica
WP_126485345.1	Antibiotic biosynthesis monooxygenase [Serratia plymuthica]	Serratia plymuthica
WP_126485476.1	Glutamate synthase large subunit	Serratia plymuthica
WP_126485488.1	UDP-N-acetylglucosamine 1-carboxyvinyltransferase	Serratia plymuthica
WP_126485494.1	Phospholipid-binding protein MlaC	Serratia plymuthica
WP_126485560.1	Rod shape-determining protein	Enterobacterales
WP_126485572.1	Acetyl-CoA carboxylase biotin carboxylase subunit	Serratia plymuthica
WP_126485607.1	Single-stranded DNA-binding protein SSB1	Serratia plymuthica
WP_126485701.1	FKBP-type peptidyl-prolyl cis-trans isomerase	Serratia plymuthica
WP_126485743.1	Tryptophan--tRNA ligase	Serratia plymuthica
WP_126485884.1	Inorganic phosphate transporter PitA	Serratia plymuthica
WP_126485956.1	ATP-independent periplasmic protein-refolding chaperone	Serratia plymuthica
WP_126485974.1	Ketol-acid reductoisomerase	Serratia plymuthica
WP_126486035.1	Glycerol kinase GlpK	Serratia plymuthica
WP_126486053.1	Protein-export chaperone SecB	Serratia plymuthica
WP_126486090.1	YicC family protein	Serratia plymuthica
WP_126486128.1	Ribosome-dependent GTPase TypA	Serratia plymuthica
WP_126486171.1	Phosphate ABC transporter substrate-binding protein PstS	Serratia plymuthica
WP_126486177.1	ABC transporter substrate-binding protein	Serratia plymuthica
WP_126486190.1	NAD(P)H-dependent oxidoreductase	Serratia plymuthica
WP_126486279.1	2-oxoglutarate dehydrogenase E1 component	Serratia plymuthica
WP_126486293.1	Methionine ABC transporter substrate-binding protein	Serratia plymuthica
WP_126486297.1	S-(hydroxymethyl)glutathione dehydrogenase/class III alcohol dehydrogenase	Serratia plymuthica
WP_126486343.1	Bifunctional UDP-4-amino-4-deoxy-l-arabinose formyltransferase/UDP-glucuronic acid oxidase ArnA	Serratia plymuthica
WP_126486374.1	Diaminobutyrate--2-oxoglutarate transaminase	Serratia plymuthica
WP_126486390.1	Zinc ABC transporter substrate-binding protein ZnuA	Serratia plymuthica
WP_126527292.1	DNA topoisomerase (ATP-hydrolyzing) subunit B	Serratia plymuthica
WP_126527305.1	Glycine--tRNA ligase subunit beta	Serratia plymuthica
WP_126527311.1	Superoxide dismutase [Mn]	Serratia plymuthica
WP_126527372.1	Uridine phosphorylase	Serratia plymuthica
WP_126527385.1	Aspartate ammonia-lyase	Serratia plymuthica
WP_126527387.1	Fumarate reductase subunit FrdC	Serratia plymuthica
WP_126527388.1	Succinate dehydrogenase/fumarate reductase iron-sulfur subunit	Serratia plymuthica
WP_126527389.1	Fumarate reductase (quinol) flavoprotein subunit	Serratia plymuthica
WP_126527395.1	N-acetylmuramoyl-L-alanine amidase AmiB	Serratia plymuthica
WP_126527408.1	N-acetyltransferase	Serratia plymuthica
WP_126527417.1	PTS trehalose transporter subunit IIbc	Serratia plymuthica
WP_126527429.1	Valine--tRNA ligase	Serratia plymuthica
WP_126527433.1	Carbonic anhydrase	Serratia plymuthica
WP_126527472.1	Molybdopterin adenylyltransferase	Serratia plymuthica

Protein code	Protein identification	Reference organism
WP_126527482.1	Thiamine ABC transporter substrate binding subunit	Serratia plymuthica
WP_126527496.1	Serine endoprotease DegP	Serratia plymuthica
WP_126527517.1	Murein hydrolase activator NlpD	Serratia plymuthica
WP_126527586.1	Hypothetical protein	Serratia plymuthica
WP_126527624.1	Lipoyl synthase	Serratia plymuthica
WP_126527625.1	D-alanyl-D-alanine carboxypeptidase DacA	Serratia plymuthica
WP_126527630.1	Leucine--tRNA ligase	Serratia plymuthica
WP_126527633.1	N-acetylglucosamine-6-phosphate deacetylase	Serratia plymuthica
WP_126527646.1	3-deoxy-7-phosphoheptulonate synthase AroG	Serratia plymuthica
WP_126527665.1	Adenosylhomocysteinase	Serratia plymuthica
WP_126527752.1	UTP--glucose-1-phosphate uridylyltransferase GalU	Serratia plymuthica
WP_126527781.1	Pyridoxal-phosphate dependent enzyme	Serratia plymuthica
WP_126527796.1	Cell division protein FtsK	Serratia plymuthica
WP_126527801.1	Formate transporter FocA	Serratia plymuthica
WP_126527862.1	Hypothetical protein	Serratia plymuthica
WP_126527876.1	Ribonuclease E	Serratia plymuthica
WP_126527887.1	Non-heme ferritin	Serratia plymuthica
WP_126527912.1	Trans-2-enoyl-CoA reductase family protein	Serratia plymuthica
WP_126528017.1	ATP-dependent dethiobiotin synthetase BioD	Serratia plymuthica
WP_126528198.1	Outer membrane protein OmpW	Serratia plymuthica
WP_126528222.1	Copper homeostasis protein CutC	Serratia plymuthica
WP_126528225.1	Arginine--tRNA ligase	Serratia plymuthica
WP_126528421.1	Citrate (pro-3S)-lyase subunit beta	Serratia plymuthica
WP_126528468.1	DNA topoisomerase (ATP-hydrolyzing) subunit A	Serratia plymuthica
WP_126528469.1	Ribonucleoside-diphosphate reductase subunit alpha	Serratia plymuthica
WP_126528470.1	1,4-dihydroxy-2-naphthoyl-CoA synthase	Serratia plymuthica
WP_126528531.1	Cysteine synthase A	Serratia plymuthica
WP_126528575.1	Class I fructose-bisphosphate aldolase	Serratia plymuthica
WP_126528619.1	DUF3131 domain-containing protein	Serratia plymuthica
WP_126528642.1	TRNA/rRNA methyltransferase	Serratia plymuthica
WP_126528651.1	Outer membrane protein assembly factor BamA	Serratia plymuthica
WP_126528657.1	YgdI/YgdR family lipoprotein	Serratia plymuthica
WP_126528703.1	Phosphoglycerate dehydrogenase	Serratia plymuthica
WP_126528704.1	Oxidative stress defense protein	Serratia plymuthica
WP_126528787.1	Glycerol dehydrogenase	Serratia plymuthica
WP_126528793.1	tRNA (adenosine(37)-N6)-threonylcarbamoyltransferase complex transferase subunit TsaD	Serratia plymuthica
WP_126528827.1	Acetyl-CoA carboxylase biotin carboxyl carrier protein	Serratia plymuthica
WP_126528838.1	Excinuclease ABC subunit UvrA	Serratia plymuthica
WP_126528846.1	Maltose/maltodextrin ABC transporter substrate-binding protein MalE	Serratia plymuthica
WP_126528863.1	Peptidylprolyl isomerase	Serratia plymuthica
WP_126528873.1	DUF853 family protein	Serratia plymuthica
WP_126528909.1	Autoinducer 2 ABC transporter substrate-binding protein	Serratia plymuthica
WP_126528913.1	Oligopeptidase A	Serratia plymuthica
WP_126528923.1	Dihydroxy-acid dehydratase	Serratia plymuthica
WP_126528932.1	Met regulon transcriptional regulator MetJ	Serratia plymuthica
WP_126528997.1	Galactokinase	Serratia plymuthica
WP_126529004.1	GalU regulator GalF	Serratia plymuthica
WP_126529065.1	Ribose ABC transporter substrate-binding protein RbsB	Serratia plymuthica
WP_145917148.1	Serine-type D-Ala-D-Ala carboxypeptidase	Serratia plymuthica
WP_148661052.1	Peptide-methionine (S)-S-oxide reductase MsrA	Serratia plymuthica
WP_148661053.1	Sulfate adenylyltransferase subunit CysN	Serratia plymuthica
WP_148661070.1	Glucokinase	Serratia plymuthica
WP_148669238.1	Formate dehydrogenase-N subunit alpha	Serratia plymuthica
WP_148669243.1	3-hydroxyacyl-ACP dehydratase FabZ	Serratia plymuthica

Table S3. Differential expression of proteins in Ra4 (*Rahnella aquatilis*) treated with 0.4% NaCl vs Control.

Row	Index	Protein code	Protein identification	Reference organism	p-value	Fold Change	Log (Fold Change)
157	169	WP_113877445.1	FliC/FljB family flagellin	Rahnella aquatilis	0.0048	0.013243427	-1.877999625
250	588	WP_013575778.1	MULTISPECIES: FliC/FljB family flagellin	Rahnella sp.	0.01703	0.017814571	-1.749224629
56	326	WP_047609302.1	chemotaxis protein CheW	Rahnella aquatilis	0.00032	0.03040935	-1.516992867
11	613	WP_112288147.1	MULTISPECIES: peptide deformylase	Rahnella sp.	3.66E-05	0.043071557	-1.365809425
289	423	WP_112290707.1	MULTISPECIES: PTS glucose transporter subunit IIA	Rahnella sp.	0.02871	0.05096507	-1.292727376
18	478	WP_131638206.1	phosphoribosylformylglycinamidine synthase	Rahnella aquatilis	6.72E-05	0.075575677	-1.121617954
159	643	WP_112197122.1	MULTISPECIES: undecaprenyl-phosphate 4-deoxy-4-formamido-L-arabinose transferase	Rahnella sp.	0.00492	0.076578968	-1.115890491
245	120	WP_122288902.1	matrixin family metalloprotease	Serratia plymuthica	0.01577	0.08052534	-1.094067434
264	402	WP_073439293.1	flagellar hook assembly protein FlgD	Serratia plymuthica	0.01948	0.086506604	-1.062950738
32	374	AGO55878.1	alpha-galactosidase MelA	Serratia plymuthica 4Rx13	0.00013	0.089424298	-1.048544463
8	17	WP_122288670.1	flagellar hook-associated protein FlgK	Serratia plymuthica	2.47E-05	0.094506119	-1.02454007
23	103	WP_112197123.1	MULTISPECIES: bifunctional UDP-4-amino-4-deoxy-L-arabinose formyltransferase/UDP-glucuronic acid oxidase ArnA	Rahnella sp.	9.51E-05	0.101017683	-0.995602596
256	218	WP_065506321.1	MULTISPECIES: pyruvate kinase	Serratia sp.	0.01821	0.104143274	-0.982368773
182	560	WP_131638012.1	phage tail sheath protein	Rahnella aquatilis	0.00797	0.104880764	-0.979304156
91	158	WP_122289835.1	matrixin family metalloprotease	Serratia plymuthica	0.00097	0.115805986	-0.936268991
117	621	WP_014333680.1	HAMP domain-containing protein	Rahnella aquatilis	0.00185	0.117243237	-0.9309122
315	386	WP_113876955.1	glutamate--tRNA ligase	Rahnella aquatilis	0.03761	0.12898579	-0.889458132
326	614	WP_037433008.1	outer membrane protein OmpW	Serratia plymuthica	0.04322	0.129590449	-0.887427004
150	659	WP_006324221.1	MULTISPECIES: D-alanine--D-alanine ligase	Serratia sp.	0.00448	0.16959818	-0.770578812
88	710	WP_126528873.1	DUF853 family protein	Serratia plymuthica	0.00093	0.202929681	-0.692654427
336	353	WP_015698114.1	succinate dehydrogenase iron-sulfur subunit	Rahnella aquatilis	0.04814	0.205885606	-0.686374016
153	377	WP_126485956.1	ATP-independent periplasmic protein-refolding chaperone	Serratia plymuthica	0.00458	0.224236154	-0.649294363
340	807	WP_062869793.1	DUF2501 domain-containing protein	Serratia plymuthica	0.04981	0.226595451	-0.644748814
283	853	WP_006322514.1	MULTISPECIES: 23S rRNA accumulation protein YceD	Serratia sp.	0.02529	0.2364057	-0.626342057
320	618	WP_006322046.1	MULTISPECIES: elongation factor P	Serratia sp.	0.04012	0.238759467	-0.622039399
319	379	WP_122289388.1	maltose/maltodextrin ABC transporter substrate-binding protein MalE	Serratia plymuthica	0.03908	0.238969634	-0.621657282
274	474	WP_112289342.1	MULTISPECIES: glycerol kinase GlpK	Rahnella sp.	0.02204	0.239061508	-0.621490346
90	48	WP_112290864.1	MULTISPECIES: glutamine--fructose-6-phosphate transaminase (isomerizing)	Rahnella sp.	0.00096	0.261280956	-0.582892243
323	7	WP_004945953.1	MULTISPECIES: flagellar filament capping protein FliD	Serratia sp.	0.0419	0.262298201	-0.581204689
44	309	WP_126528787.1	glycerol dehydrogenase	Serratia plymuthica	0.00018	0.271924037	-0.565552401

Row	Index	Protein code	Protein identification	Reference organism	p-value	Fold Change	Log (Fold Change)
180	821	WP_006319070.1	MULTISPECIES: ribulose-phosphate 3-epimerase	Serratia sp.	0.00714	0.277532813	-0.556685662
110	628	WP_131638522.1	guanylate kinase	Rahnella aquatilis	0.00157	0.277999707	-0.555955663
252	790	WP_126529004.1	GalU regulator GalF	Serratia plymuthica	0.01745	0.282958724	-0.548276912
103	281	WP_113878254.1	aminomethyl-transferring glycine dehydrogenase	Rahnella aquatilis	0.00137	0.285681729	-0.544117535
16	185	WP_112290710.1	MULTISPECIES: cysteine synthase A	Rahnella sp.	5.71E-05	0.286049602	-0.543558652
279	348	WP_126481625.1	3-phosphoserine/phosphohydroxythreonine transaminase	Serratia plymuthica	0.0236	0.28889226	-0.539264093
249	249	WP_013577652.1	MULTISPECIES: glutamate--ammonia ligase	Rahnella sp.	0.01611	0.294674486	-0.530657465
302	358	WP_112290918.1	MULTISPECIES: xanthine phosphoribosyltransferase	Rahnella sp.	0.03308	0.310067868	-0.508543237
25	567	WP_015698255.1	phosphoribosylformylglycinamidine cyclo-ligase	Rahnella aquatilis	9.63E-05	0.316959074	-0.49899681
120	611	WP_013812178.1	MULTISPECIES: galactose/glucose ABC transporter substrate-binding protein MglB	Serratia sp.	0.002	0.324112862	-0.489303734
209	739	WP_126484047.1	1-phosphofructokinase	Serratia plymuthica	0.01103	0.328934239	-0.482890918
163	237	WP_131638766.1	ATP-dependent Clp protease ATP-binding subunit ClpA	Rahnella aquatilis	0.00507	0.330828472	-0.480397121
333	276	WP_013573634.1	MULTISPECIES: cAMP-activated global transcriptional regulator CRP	Yersiniaceae	0.047	0.334773782	-0.475248561
277	489	WP_073440262.1	aspartate-semialdehyde dehydrogenase	Serratia plymuthica	0.02332	0.336726414	-0.472722815
221	401	WP_113877256.1	diaminobutyrate--2-oxoglutarate transaminase	Rahnella aquatilis	0.0122	0.34086544	-0.46741703
324	128	WP_013811266.1	MULTISPECIES: malate dehydrogenase	Serratia sp.	0.04221	0.34299365	-0.46471392
299	146	WP_006317741.1	MULTISPECIES: triose-phosphate isomerase	Serratia sp.	0.03216	0.34528417	-0.461823332
195	785	WP_126482255.1	RNA chaperone ProQ	Serratia plymuthica	0.00944	0.346126942	-0.460764594
141	781	WP_062868532.1	phosphoglycerate dehydrogenase	Serratia plymuthica	0.00385	0.346548292	-0.460236237
205	470	WP_015696652.1	formate transporter FocA	Rahnella aquatilis	0.01072	0.350113543	-0.45579109
179	344	WP_095925026.1	MULTISPECIES: uracil phosphoribosyltransferase	Rahnella sp.	0.00703	0.355261021	-0.449452441
31	336	WP_119261297.1	MetQ/NlpA family lipoprotein	Rahnella aquatilis	0.00012	0.357408909	-0.446834626
332	460	WP_047608889.1	phosphoribosylaminoimidazolesuccinocarboxamide synthase	Rahnella aquatilis	0.04596	0.359224323	-0.444634264
154	236	WP_073439268.1	PLP-dependent transferase	Serratia plymuthica	0.00462	0.364174638	-0.438690303
35	512	WP_126485047.1	aminomethyl-transferring glycine dehydrogenase	Serratia plymuthica	0.00013	0.36628356	-0.436182574
235	390	WP_006321796.1	MULTISPECIES: F0F1 ATP synthase subunit gamma	Serratia sp.	0.01407	0.374340327	-0.426733385
134	398	WP_013577537.1	MULTISPECIES: thioredoxin TrxA	Yersiniaceae	0.00332	0.374934306	-0.42604482
337	112	WP_112289181.1	MULTISPECIES: succinate dehydrogenase flavoprotein subunit	Rahnella sp.	0.04837	0.37616433	-0.424622389
84	466	WP_112168192.1	MULTISPECIES: two-component system response regulator ArcA	Rahnella sp.	0.00088	0.377163081	-0.423470826
118	795	WP_063177426.1	acetolactate synthase AlsS	Serratia plymuthica	0.00188	0.378852069	-0.421530337
52	13	WP_006325792.1	bifunctional acetaldehyde-CoA/alcohol dehydrogenase	Serratia plymuthica	0.00027	0.379442539	-0.420853982
237	51	WP_004946072.1	MULTISPECIES: flagellar hook-filament junction protein FlgL	Serratia sp.	0.01426	0.380159224	-0.420034467
149	255	WP_004952855.1	MULTISPECIES: transcription termination/antitermination protein NusA	Serratia sp.	0.00448	0.388432791	-0.410684114
262	137	WP_131637279.1	porin OmpC	Rahnella aquatilis	0.01885	0.389544746	-0.409442649

Row	Index	Protein code	Protein identification	Reference organism	p-value	Fold Change	Log (Fold Change)
28	22	WP_015697077.1	bifunctional acetaldehyde-CoA/alcohol dehydrogenase	Rahnella aquatilis	0.00011	0.391342469	-0.407443019
82	285	WP_065506483.1	MULTISPECIES: serine hydroxymethyltransferase	Serratia sp.	0.00081	0.393619308	-0.404923606
220	818	WP_037433374.1	GSH-dependent disulfide bond oxidoreductase	Serratia plymuthica	0.01219	0.394341782	-0.404127205
216	351	WP_063200622.1	phosphoenolpyruvate carboxykinase (ATP)	Serratia plymuthica	0.01201	0.394390643	-0.404073397
230	363	WP_015696434.1	amidophosphoribosyltransferase	Rahnella aquatilis	0.01312	0.398048611	-0.400063887
130	258	WP_013577593.1	MULTISPECIES: triose-phosphate isomerase	Rahnella sp.	0.00274	0.401240229	-0.396595531
107	221	WP_063198636.1	energy-dependent translational throttle protein EttA	Serratia plymuthica	0.00151	0.402378869	-0.395364834
218	235	WP_122289073.1	multicopper oxidase CueO	Serratia plymuthica	0.01204	0.403629267	-0.394017351
233	658	WP_126480223.1	catabolite repressor/activator	Serratia plymuthica	0.01391	0.405613065	-0.391888065
297	302	WP_015698930.1	acetyl-CoA carboxylase biotin carboxylase subunit	Rahnella aquatilis	0.03155	0.409216652	-0.388046702
301	801	WP_073439566.1	peptidylprolyl isomerase	Serratia plymuthica	0.03258	0.409942937	-0.387276592
310	296	WP_047609936.1	glycine--tRNA ligase subunit beta	Rahnella aquatilis	0.03612	0.410035135	-0.387178928
99	284	WP_015697179.1	glucose-6-phosphate dehydrogenase	Rahnella aquatilis	0.0012	0.411042894	-0.386112855
128	153	WP_037432618.1	asparagine--tRNA ligase	Serratia plymuthica	0.00242	0.414649962	-0.38231837
57	657	WP_004932501.1	MULTISPECIES: 30S ribosomal protein S16	Enterobacterales	0.00032	0.417234937	-0.379619333
41	67	WP_037035301.1	MULTISPECIES: pyruvate kinase PykF	Rahnella sp.	0.00016	0.419066368	-0.377717192
303	52	WP_113877103.1	MULTISPECIES: malate dehydrogenase	Rahnella sp.	0.03352	0.423359123	-0.373291078
69	465	WP_013815047.1	MULTISPECIES: glutamate--ammonia ligase	Serratia sp.	0.00055	0.425977717	-0.370613119
271	516	WP_126528846.1	maltose/maltodextrin ABC transporter substrate-binding protein MalE	Serratia plymuthica	0.02084	0.430363754	-0.366164313
2	26	WP_095924049.1	MULTISPECIES: F0F1 ATP synthase subunit beta	Rahnella sp.	5.28E-06	0.432188265	-0.364327029
321	509	WP_126481850.1	universal stress protein	Serratia plymuthica	0.04023	0.43496145	-0.361549232
244	204	WP_015696675.1	asparagine--tRNA ligase	Rahnella aquatilis	0.01552	0.437841776	-0.358682803
261	680	WP_122288801.1	aminomethyl-transferring glycine dehydrogenase	Serratia plymuthica	0.01856	0.437928352	-0.358596937
47	305	WP_131638044.1	ribonuclease E	Rahnella aquatilis	0.00023	0.440899751	-0.355660146
286	238	WP_013577239.1	MULTISPECIES: 50S ribosomal protein L13	Yersiniaceae	0.02749	0.443030743	-0.353566136
9	268	WP_013576134.1	MULTISPECIES: 50S ribosomal protein L20	Yersiniaceae	2.87E-05	0.449540058	-0.347231602
140	168	WP_004947678.1	MULTISPECIES: cysteine synthase A	Serratia sp.	0.0038	0.45437863	-0.342582102
106	98	WP_126527385.1	aspartate ammonia-lyase	Serratia plymuthica	0.00149	0.458567358	-0.338596862
188	16	WP_014333640.1	chaperonin GroEL	Rahnella aquatilis	0.00861	0.458878989	-0.338301827
142	475	WP_126486128.1	ribosome-dependent GTPase TypA	Serratia plymuthica	0.00392	0.460052231	-0.337192859
61	292	WP_062870646.1	pyruvate kinase PykF	Serratia plymuthica	0.00038	0.460963137	-0.336333804
89	541	WP_047609862.1	aspartate-semialdehyde dehydrogenase	Rahnella aquatilis	0.00094	0.462128288	-0.335237447
62	726	WP_004933536.1	MULTISPECIES: ribosome assembly RNA-binding protein YhbY	Enterobacterales	0.00038	0.465599351	-0.331987634
78	28	WP_013577682.1	MULTISPECIES: F0F1 ATP synthase subunit alpha	Rahnella sp.	0.00066	0.466245846	-0.331385024
36	147	WP_112289290.1	MULTISPECIES: ribosome-dependent GTPase TypA	Rahnella sp.	0.00014	0.466767875	-0.330899041

Row	Index	Protein code	Protein identification	Reference organism	p-value	Fold Change	Log (Fold Change)
334	449	WP_112288379.1	MULTISPECIES: UDP-N-acetylglucosamine 1-carboxyvinyltransferase	Rahnella sp.	0.04741	0.466784874	-0.330883226
116	623	WP_126483217.1	aspartate--tRNA ligase	Serratia plymuthica	0.00181	0.466935894	-0.33074274
194	838	WP_126527624.1	lipoyl synthase	Serratia plymuthica	0.00927	0.471989067	-0.326068061
87	459	WP_113876048.1	glutamine--tRNA ligase	Rahnella aquatilis	0.00091	0.472267976	-0.325811503
200	217	WP_013577410.1	MULTISPECIES: 50S ribosomal protein L7/L12	Yersiniaceae	0.01006	0.472850637	-0.325276021
291	697	WP_119261434.1	aspartate/tyrosine/aromatic aminotransferase	Rahnella aquatilis	0.02895	0.473858624	-0.324351211
66	441	WP_004947686.1	MULTISPECIES: PTS glucose transporter subunit IIA	Serratia sp.	0.00047	0.47520989	-0.323114529
254	92	WP_005969574.1	MULTISPECIES: 30S ribosomal protein S7	Enterobacterales	0.01762	0.475654901	-0.322708024
335	665	WP_013573696.1	MULTISPECIES: 30S ribosomal protein S14	Yersiniaceae	0.04781	0.476134346	-0.32227049
95	350	WP_095924936.1	MULTISPECIES: 6,7-dimethyl-8-ribityllumazine synthase	Rahnella sp.	0.00113	0.478070016	-0.320508494
38	472	WP_004931195.1	MULTISPECIES: 50S ribosomal protein L28	Enterobacterales	0.00015	0.478382723	-0.320224513
170	27	WP_015695890.1	phosphopyruvate hydratase	Rahnella aquatilis	0.00565	0.47886815	-0.319784048
139	671	WP_126528470.1	1,4-dihydroxy-2-naphthoyl-CoA synthase	Serratia plymuthica	0.00372	0.480385944	-0.318409709
162	769	WP_006318638.1	MULTISPECIES: MetQ/NlpA family lipoprotein	Serratia sp.	0.005	0.483056516	-0.316002055
3	590	WP_056772785.1	MULTISPECIES: 30S ribosomal protein S16	Yersiniaceae	7.43E-06	0.486097279	-0.31327681
189	471	WP_004943136.1	MULTISPECIES: trans-2-enoyl-CoA reductase family protein	Serratia sp.	0.00893	0.487288391	-0.312213935
127	224	WP_002221949.1	MULTISPECIES: transcription antiterminator/RNA stability regulator CspE	Enterobacterales	0.00241	0.496734815	-0.3038754
176	754	WP_126479796.1	FtsH protease activity modulator HflK	Serratia plymuthica	0.00642	0.497667424	-0.303060786
199	72	WP_062870323.1	NADP-dependent phosphogluconate dehydrogenase	Serratia plymuthica	0.00987	0.500853765	-0.300289057
86	173	WP_006321797.1	MULTISPECIES: F0F1 ATP synthase subunit alpha	Serratia sp.	0.0009	0.504219584	-0.29738029
129	506	WP_122289463.1	phenylalanine--tRNA ligase subunit beta	Serratia plymuthica	0.00248	0.504554547	-0.297091876
213	548	WP_126527876.1	ribonuclease E	Serratia plymuthica	0.0116	0.508080271	-0.294067669
55	89	WP_119261052.1	DNA topoisomerase (ATP-hydrolyzing) subunit B	Rahnella aquatilis	0.0003	0.510944048	-0.291626656
60	37	WP_002919219.1	MULTISPECIES: DNA-directed RNA polymerase subunit alpha	Enterobacterales	0.00038	0.514073917	-0.288974431
125	148	WP_113877073.1	aspartate ammonia-lyase	Rahnella aquatilis	0.00216	0.515686621	-0.287614136
305	269	WP_004951161.1	MULTISPECIES: 50S ribosomal protein L14	Serratia sp.	0.03458	0.517956656	-0.285706582
268	440	WP_004945505.1	pyruvate kinase	Serratia plymuthica	0.02026	0.519408293	-0.284491121
101	199	WP_004943793.1	MULTISPECIES: glycine zipper 2TM domain-containing protein	Serratia sp.	0.00128	0.521596152	-0.282665621
145	287	WP_015690190.1	MULTISPECIES: acyl carrier protein	Yersiniaceae	0.0042	0.522644471	-0.281793639
311	703	WP_004943598.1	MULTISPECIES: phenylalanine--tRNA ligase subunit alpha	Serratia sp.	0.03682	0.526929851	-0.278247197
243	187	WP_004951135.1	MULTISPECIES: 50S ribosomal protein L15	Serratia sp.	0.01548	0.527006247	-0.278184237
164	387	WP_126484836.1	2,3,4,5-tetrahydropyridine-2,6-dicarboxylate N-succinyltransferase	Serratia plymuthica	0.00509	0.529296342	-0.276301107
143	117	WP_047607355.1	serine hydroxymethyltransferase	Rahnella aquatilis	0.00407	0.534246719	-0.272258136
270	212	WP_006323137.1	MULTISPECIES: 50S ribosomal protein L7/L12	Serratia sp.	0.02043	0.534864269	-0.271756414
215	343	WP_004952787.1	MULTISPECIES: inorganic diphosphatase	Serratia sp.	0.01187	0.536129513	-0.270730285

Row	Index	Protein code	Protein identification	Reference organism	p-value	Fold Change	Log (Fold Change)
167	291	WP_047606852.1	3-deoxy-8-phosphooctulonate synthase	Rahnella aquatilis	0.00528	0.537667363	-0.269486325
183	45	WP_013811522.1	MULTISPECIES: phosphopyruvate hydratase	Serratia sp.	0.00818	0.538947063	-0.26845389
68	21	WP_062791875.1	MULTISPECIES: elongation factor Tu, partial	Serratia sp.	0.00052	0.539219266	-0.268234599
173	176	WP_071823626.1	MULTISPECIES: ribose-phosphate pyrophosphokinase	Rahnella sp.	0.00626	0.545575537	-0.263145112
295	99	WP_113878239.1	MULTISPECIES: phosphoglycerate kinase	Rahnella sp.	0.03059	0.545839251	-0.262935237
70	270	WP_004943595.1	MULTISPECIES: 50S ribosomal protein L20	Serratia sp.	0.00056	0.54774473	-0.261421793
72	116	WP_013573700.1	MULTISPECIES: 30S ribosomal protein S5	Yersiniaceae	0.00059	0.547920559	-0.261282403
131	335	WP_112287508.1	MULTISPECIES: ATP-dependent protease ATP-binding subunit ClpX	Rahnella sp.	0.00277	0.548765261	-0.260613389
285	495	WP_062869945.1	citrate synthase	Serratia plymuthica	0.02643	0.550249061	-0.25944069
160	676	WP_015690312.1	MULTISPECIES: protein translocase subunit SecD	Rahnella sp.	0.00497	0.551412829	-0.258523134
309	694	WP_062868807.1	DUF883 domain-containing protein	Serratia plymuthica	0.0359	0.552545184	-0.257632202
266	660	WP_037033577.1	MULTISPECIES: transaldolase	Rahnella sp.	0.02001	0.554099452	-0.256412279
246	681	WP_086935265.1	MULTISPECIES: 3-hydroxyacyl-ACP dehydratase FabZ	Rahnella sp.	0.01585	0.556938798	-0.254192527
325	151	WP_004952226.1	MULTISPECIES: 30S ribosomal protein S2	Serratia sp.	0.04262	0.558323238	-0.253114296
223	734	WP_095925051.1	MULTISPECIES: bifunctional methylenetetrahydrofolate dehydrogenase/methenyltetrahydrofolate cyclohydrolase FolD	Rahnella sp.	0.01249	0.559440643	-0.252245986
171	11	WP_013573670.1	MULTISPECIES: elongation factor Tu	Rahnella sp.	0.00609	0.560662376	-0.251298587
45	558	WP_126486035.1	glycerol kinase GlpK	Serratia plymuthica	0.00018	0.563671718	-0.248973756
202	178	WP_113877398.1	CTP synthase (glutamine hydrolyzing)	Rahnella aquatilis	0.01023	0.5649762	-0.247969847
156	492	WP_002210155.1	MULTISPECIES: 30S ribosomal protein S18	Enterobacterales	0.00478	0.566877055	-0.246511121
228	729	WP_113877923.1	3-deoxy-7-phosphoheptulonate synthase AroG	Rahnella aquatilis	0.01295	0.566925395	-0.246474089
161	677	WP_013573791.1	MULTISPECIES: inorganic diphosphatase	Rahnella sp.	0.00499	0.567072466	-0.246361439
136	454	WP_064798761.1	phosphomannomutase CpsG	Serratia plymuthica	0.0034	0.568212802	-0.245488985
77	453	WP_126527389.1	fumarate reductase (quinol) flavoprotein subunit	Serratia plymuthica	0.00065	0.569304293	-0.244655542
147	141	WP_015699040.1	50S ribosomal protein L1	Rahnella aquatilis	0.0043	0.56979219	-0.244283508
284	124	WP_122289796.1	DUF2974 domain-containing protein	Serratia plymuthica	0.02548	0.57004908	-0.244087751
318	69	WP_047609285.1	NADP-dependent phosphogluconate dehydrogenase	Rahnella aquatilis	0.03902	0.573815053	-0.241228063
212	672	WP_013574367.1	MULTISPECIES: nucleoside-diphosphate kinase	Yersiniaceae	0.01122	0.576171437	-0.239448275
229	394	WP_126528198.1	outer membrane protein OmpW	Serratia plymuthica	0.01303	0.578891158	-0.237403084
276	82	WP_113876357.1	MULTISPECIES: pyruvate dehydrogenase complex dihydrolipoyllysine-residue acetyltransferase	Rahnella sp.	0.02328	0.581000916	-0.235823183
175	646	WP_004951171.1	MULTISPECIES: 50S ribosomal protein L16	Serratia sp.	0.00639	0.581323642	-0.235582014
196	525	WP_004953928.1	MULTISPECIES: transcription termination/antitermination protein NusG	Serratia sp.	0.00953	0.582461033	-0.234733124
192	431	WP_113876187.1	MULTISPECIES: elongation factor 4	Rahnella sp.	0.00915	0.582832537	-0.234456212
292	154	WP_063198834.1	F0F1 ATP synthase subunit beta	Serratia plymuthica	0.02959	0.582906271	-0.234401273

Row	Index	Protein code	Protein identification	Reference organism	p-value	Fold Change	Log (Fold Change)
83	214	WP_006317766.1	argininosuccinate synthase	Serratia plymuthica	0.00087	0.583708657	-0.233803866
197	310	WP_065505698.1	MULTISPECIES: thioredoxin TrxA	Serratia sp.	0.00981	0.584850459	-0.232955165
30	61	WP_004952224.1	MULTISPECIES: elongation factor Ts	Serratia sp.	0.00012	0.586149741	-0.231991423
207	25	WP_013811127.1	MULTISPECIES: DNA-directed RNA polymerase subunit beta'	Serratia sp.	0.01084	0.587053255	-0.2313225
204	405	WP_013811241.1	MULTISPECIES: protease modulator HflC	Serratia sp.	0.01045	0.591028883	-0.228391295
190	496	WP_119261854.1	alpha-D-glucose phosphate-specific phosphoglucomutase	Rahnella aquatilis	0.00902	0.592428065	-0.227364376
316	55	WP_113877112.1	MULTISPECIES: polyribonucleotide nucleotidyltransferase	Rahnella sp.	0.03799	0.595684042	-0.224984034
109	18	WP_015699039.1	MULTISPECIES: DNA-directed RNA polymerase subunit beta	Rahnella sp.	0.00156	0.596572799	-0.224336553
81	439	WP_013575324.1	MULTISPECIES: septum site-determining protein MinD	Rahnella sp.	0.00077	0.600882041	-0.221210776
313	298	WP_004942906.1	MULTISPECIES: universal stress protein	Serratia sp.	0.03752	0.601092735	-0.221058521
296	4	WP_095924286.1	MULTISPECIES: DNA-directed RNA polymerase subunit beta'	Rahnella sp.	0.03075	0.604831856	-0.218365343
330	544	WP_126527912.1	trans-2-enoyl-CoA reductase family protein	Serratia plymuthica	0.04491	0.608022047	-0.216080673
317	395	WP_119261922.1	glycine cleavage system aminomethyltransferase GcvT	Rahnella aquatilis	0.03835	0.61036089	-0.214413302
185	95	WP_015698322.1	MULTISPECIES: endopeptidase La	Rahnella sp.	0.00846	0.611209103	-0.213810186
119	149	WP_004951140.1	MULTISPECIES: 30S ribosomal protein S5	Serratia sp.	0.00193	0.617386406	-0.209442938
206	589	WP_126483203.1	glucose-6-phosphate dehydrogenase	Serratia plymuthica	0.01073	0.618311167	-0.20879291
307	108	WP_004391423.1	MULTISPECIES: 50S ribosomal protein L22	Enterobacterales	0.03556	0.618498095	-0.208661634
39	616	WP_113877630.1	glycine C-acetyltransferase	Rahnella aquatilis	0.00015	0.621651498	-0.206453016
247	223	WP_112286795.1	MULTISPECIES: ATP-dependent zinc metalloprotease FtsH	Rahnella sp.	0.016	0.623193039	-0.205377406
211	60	WP_063203045.1	DNA-directed RNA polymerase subunit beta'	Serratia plymuthica	0.01118	0.62759014	-0.202323888
253	290	WP_020837234.1	MULTISPECIES: 50S ribosomal protein L18	Serratia sp.	0.01757	0.631488538	-0.199634528
294	216	WP_112289497.1	MULTISPECIES: DNA topoisomerase (ATP-hydrolyzing) subunit A	Rahnella sp.	0.0299	0.634678288	-0.197446358
193	62	WP_013573691.1	MULTISPECIES: 30S ribosomal protein S3	Rahnella sp.	0.00925	0.635566926	-0.196838711
255	83	WP_037431412.1	phosphate acetyltransferase	Serratia plymuthica	0.01804	0.636137948	-0.196448696
281	198	WP_014333606.1	MULTISPECIES: 30S ribosomal protein S4	Rahnella sp.	0.02447	0.641597931	-0.192737045
293	687	WP_073440429.1	alpha-D-glucose phosphate-specific phosphoglucomutase	Serratia plymuthica	0.02985	0.642219415	-0.192316569
236	86	WP_004951125.1	MULTISPECIES: 30S ribosomal protein S4	Serratia sp.	0.01409	0.642235936	-0.192305398
275	584	WP_121019733.1	phosphomannomutase CpsG	Rahnella aquatilis	0.02262	0.644760396	-0.190601646
135	312	WP_008457164.1	MULTISPECIES: 50S ribosomal protein L14	Enterobacterales	0.00334	0.649753172	-0.187251592
146	144	WP_004951155.1	MULTISPECIES: 50S ribosomal protein L5	Serratia sp.	0.00428	0.650922925	-0.186470432
273	230	WP_006323136.1	MULTISPECIES: 50S ribosomal protein L10	Serratia sp.	0.02145	0.653638868	-0.184662131
225	19	WP_014333586.1	elongation factor G	Rahnella aquatilis	0.01265	0.65451755	-0.184078704
94	143	WP_013577536.1	MULTISPECIES: transcription termination factor Rho	Rahnella sp.	0.00106	0.659975087	-0.180472458
217	696	WP_004949671.1	MULTISPECIES: glutamine--tRNA ligase	Serratia sp.	0.01203	0.663159877	-0.178381758
272	253	WP_015698881.1	30S ribosomal protein S9	Rahnella aquatilis	0.02101	0.666256533	-0.176358519
238	316	WP_013577396.1	MULTISPECIES: DNA-binding protein HU-alpha	Yersiniaceae	0.01448	1.576601763	0.197722008

Row	Index	Protein code	Protein identification	Reference organism	p-value	Fold Change	Log (Fold Change)
339	135	WP_122288872.1	S9 family peptidase	Serratia plymuthica	0.04872	1.720951053	0.235768518
181	418	WP_126124940.1	iron uptake system protein EfeO	Rahnella aquatilis	0.00779	1.722714371	0.236213277
227	689	WP_015697849.1	carboxy terminal-processing peptidase	Rahnella aquatilis	0.01291	1.776202002	0.249492355
258	329	WP_004949394.1	MULTISPECIES: bifunctional UDP-sugar hydrolase/5'-nucleotidase	Serratia sp.	0.01846	1.791971144	0.253331012
201	524	WP_006317468.1	MULTISPECIES: peptidyl-prolyl cis-trans isomerase	Serratia sp.	0.01011	1.795750268	0.25424594
298	367	WP_006322224.1	hypothetical protein	Serratia plymuthica	0.03177	1.806914556	0.256937616
24	101	WP_122288887.1	serine endoprotease DegQ	Serratia plymuthica	9.61E-05	1.810642603	0.257832735
331	417	WP_126529065.1	ribose ABC transporter substrate-binding protein RbsB	Serratia plymuthica	0.04493	1.830177826	0.262493289
329	362	WP_126481301.1	glutamine ABC transporter substrate-binding protein GlnH	Serratia plymuthica	0.04409	1.862063189	0.269994415
241	12	WP_006324584.1	porin OmpA	Serratia plymuthica	0.01512	1.864457737	0.270552543
242	542	WP_126482196.1	nucleoside-specific channel-forming protein Tsx	Serratia plymuthica	0.01518	1.889072948	0.276248729
138	114	WP_126485974.1	ketol-acid reductoisomerase	Serratia plymuthica	0.00355	1.88983737	0.276424433
314	522	WP_065506637.1	MULTISPECIES: divisome-associated lipoprotein YraP	Serratia sp.	0.03755	1.914822763	0.282128582
132	814	WP_126480896.1	glucosamine-6-phosphate deaminase	Serratia plymuthica	0.00305	1.950842602	0.290222231
155	58	WP_122288888.1	penicillin-binding protein activator	Serratia plymuthica	0.00472	1.955365293	0.291227902
124	133	WP_015696692.1	porin OmpA	Rahnella aquatilis	0.00216	1.975045692	0.295577147
67	145	WP_073439860.1	carboxy terminal-processing peptidase	Serratia plymuthica	0.00048	2.015027992	0.304281083
133	297	WP_062791335.1	murein hydrolase activator NlpD	Serratia plymuthica	0.0032	2.205477714	0.343502674
290	714	WP_126484642.1	two-component system QseEF-associated lipoprotein QseG	Serratia plymuthica	0.02891	2.284952298	0.358877138
282	532	WP_112291485.1	MULTISPECIES: nucleoside-specific channel-forming protein Tsx	Rahnella sp.	0.025	2.324300774	0.366292327
260	106	WP_004942247.1	MULTISPECIES: outer membrane protein OmpX	Serratia sp.	0.01852	2.357094868	0.372377062
53	140	WP_013811470.1	MULTISPECIES: peptidylprolyl isomerase SurA	Serratia sp.	0.00027	2.380199184	0.376613302
71	38	WP_006326771.1	porin OmpC	Serratia plymuthica	0.00059	2.380979938	0.376755736
232	256	WP_006328507.1	MULTISPECIES: peptidylprolyl isomerase A	Serratia sp.	0.01391	2.429083703	0.38544248
203	207	WP_004952521.1	MULTISPECIES: oxidative stress defense protein	Serratia sp.	0.01045	2.530742803	0.40324801
248	64	WP_006323248.1	bifunctional 2',3'-cyclic-nucleotide 2'-phosphodiesterase/3'-nucleotidase	Serratia plymuthica	0.01604	2.543382296	0.405411644
257	97	WP_004950859.1	MULTISPECIES: insulinase family protein	Serratia sp.	0.01845	2.641644195	0.421874322
208	193	WP_122288956.1	L,D-transpeptidase family protein	Serratia plymuthica	0.01098	2.698326481	0.431094496
304	311	WP_004942530.1	MULTISPECIES: arginine ABC transporter substrate-binding protein	Serratia sp.	0.03381	2.716006908	0.43393087
327	718	WP_113877411.1	MULTISPECIES: murein hydrolase activator NlpD	Rahnella sp.	0.04345	2.76662515	0.441950321
172	768	WP_004950614.1	MULTISPECIES: OmpA family lipoprotein	Serratia sp.	0.00615	2.780940359	0.444191675
251	649	WP_013577556.1	MULTISPECIES: ABC transporter substrate-binding protein	Rahnella sp.	0.01703	2.85280164	0.455271575
65	664	WP_073440218.1	thioredoxin domain-containing protein	Serratia plymuthica	0.00043	2.907251454	0.463482596
187	597	WP_112290844.1	MULTISPECIES: ribose ABC transporter substrate-binding protein RbsB	Rahnella sp.	0.00848	2.922095685	0.465694433

Row	Index	Protein code	Protein identification	Reference organism	p-value	Fold Change	Log (Fold Change)
113	189	WP_006323429.1	MULTISPECIES: L,D-transpeptidase family protein	Serratia sp.	0.00171	2.984388967	0.474855426
112	257	WP_013576150.1	MULTISPECIES: carboxy terminal-processing peptidase	Rahnella sp.	0.00162	3.057842469	0.485415108
214	183	WP_063201108.1	oligopeptide ABC transporter substrate-binding protein OppA	Serratia plymuthica	0.01169	3.185314922	0.503152376
151	534	WP_062868083.1	porin	Serratia plymuthica	0.00452	3.206291528	0.506003007
102	652	WP_126528657.1	YgdI/YgdR family lipoprotein	Serratia plymuthica	0.00128	3.231149266	0.509357021
224	678	WP_062870112.1	ABC transporter substrate-binding protein	Serratia plymuthica	0.01264	3.296664992	0.518074816
114	604	WP_004942697.1	MULTISPECIES: L,D-transpeptidase	Serratia sp.	0.00177	3.340162566	0.523767604
126	389	WP_073440018.1	beta-glucosidase BglX	Serratia plymuthica	0.00219	3.509310594	0.545221807
42	6	WP_063200275.1	pitrilysin	Serratia plymuthica	0.00017	3.550336361	0.5502695
63	202	WP_065505781.1	MULTISPECIES: polyribonucleotide nucleotidyltransferase	Serratia sp.	0.00039	3.568289591	0.552460093
308	741	WP_126484412.1	transaldolase	Serratia plymuthica	0.03589	3.59308685	0.555467715
22	174	WP_122289663.1	thiol:disulfide interchange protein DsbA	Serratia plymuthica	9.27E-05	3.80273492	0.580096053
263	488	RMN17366.1	hypothetical protein ALQ63_02794	Serratia plymuthica	0.01893	4.057867722	0.608297886
148	46	WP_126527496.1	serine endoprotease DegP	Serratia plymuthica	0.0044	4.06061716	0.608592046
111	162	WP_063199821.1	serine protease inhibitor ecotin	Serratia plymuthica	0.00158	4.072858357	0.609899307
14	50	WP_047607744.1	cystine ABC transporter substrate-binding protein	Rahnella aquatilis	5.35E-05	4.128761639	0.615819811
184	391	WP_119262138.1	Do family serine endopeptidase	Rahnella aquatilis	0.0083	4.186826049	0.621884917
75	49	WP_004949773.1	MULTISPECIES: Tol-Pal system protein TolB	Serratia sp.	0.00063	4.191728086	0.622393103
33	385	WP_004949986.1	protein CreA	Serratia plymuthica	0.00013	4.191848586	0.622405587
74	110	WP_006328854.1	sulfate ABC transporter substrate-binding protein	Serratia plymuthica	0.00061	4.200643876	0.623315864
104	243	WP_047609790.1	murein transglycosylase	Rahnella aquatilis	0.00143	4.243471768	0.627721317
27	54	WP_015698105.1	Tol-Pal system protein TolB	Rahnella aquatilis	0.0001	4.302778428	0.633748983
226	612	WP_047612859.1	pitrilysin	Rahnella aquatilis	0.01278	4.37373876	0.640852839
37	226	WP_063201917.1	cystine ABC transporter substrate-binding protein	Serratia plymuthica	0.00015	4.424907603	0.645904207
123	5	WP_086016609.1	MULTISPECIES: glucans biosynthesis protein MdoG	Serratia sp.	0.00212	4.432558925	0.646654518
280	839	WP_013813653.1	MULTISPECIES: ATP-grasp domain-containing protein	Serratia sp.	0.0241	4.459216611	0.649258569
105	210	WP_047612656.1	translation initiation factor IF-2	Rahnella aquatilis	0.00148	4.647907673	0.667257493
54	282	WP_065505971.1	MULTISPECIES: cell division protein CpoB	Serratia sp.	0.00029	4.684299104	0.670644618
76	244	WP_004952410.1	MULTISPECIES: bifunctional protein-disulfide isomerase/oxidoreductase DsbC	Serratia sp.	0.00063	4.728756285	0.674746932
10	632	WP_131638597.1	phospholipid-binding protein MlaC	Rahnella aquatilis	3.32E-05	4.738399328	0.675631658
115	211	WP_112289187.1	MULTISPECIES: Tol-Pal system protein TolB	Rahnella sp.	0.00179	4.77021637	0.678538078
152	483	WP_113877622.1	MULTISPECIES: sulfate ABC transporter substrate-binding protein	Rahnella sp.	0.00455	4.867403359	0.687297337
100	3	WP_063198645.1	murein transglycosylase	Serratia plymuthica	0.00123	4.984050244	0.697582412
17	104	WP_004944994.1	DUF1471 domain-containing protein	Serratia plymuthica	6.18E-05	5.046065988	0.702952926
19	675	WP_073440332.1	glutathione ABC transporter substrate-binding protein GsiB	Serratia plymuthica	7.60E-05	5.071734469	0.705156508

Row	Index	Protein code	Protein identification	Reference organism	p-value	Fold Change	Log (Fold Change)
40	721	WP_126480972.1	Tol-Pal system protein TolB	Serratia plymuthica	0.00015	5.221408901	0.717787705
50	356	WP_004952213.1	MULTISPECIES: molecular chaperone Skp	Serratia sp.	0.00025	5.449391379	0.736348
43	250	WP_004942614.1	MULTISPECIES: outer membrane lipoprotein chaperone LolA	Serratia sp.	0.00018	5.505599761	0.740804636
265	283	WP_113878243.1	MULTISPECIES: oxidative stress defense protein	Rahnella sp.	0.01965	5.622030046	0.749893162
7	142	WP_062790903.1	MULTISPECIES: ABC transporter substrate-binding protein	Serratia sp.	1.79E-05	5.869243593	0.768582135
96	551	WP_126486390.1	zinc ABC transporter substrate-binding protein ZnuA	Serratia plymuthica	0.00113	5.890530507	0.77015441
58	442	WP_112289283.1	MULTISPECIES: thiol:disulfide interchange protein DsbA	Rahnella sp.	0.00033	6.308634418	0.799935361
122	289	WP_013573626.1	MULTISPECIES: peptidylprolyl isomerase A	Rahnella sp.	0.00207	6.454504701	0.809862921
64	433	WP_006321789.1	MULTISPECIES: phosphate ABC transporter substrate-binding protein PstS	Serratia sp.	0.00043	6.63285313	0.821700381
34	445	WP_006324721.1	MULTISPECIES: penicillin-binding protein activator LpoB	Serratia sp.	0.00013	6.794495274	0.832157201
137	380	WP_112287456.1	MULTISPECIES: ABC transporter substrate-binding protein	Rahnella sp.	0.00352	7.020911855	0.846393521
121	674	WP_064798698.1	3',5'-cyclic-nucleotide phosphodiesterase	Serratia plymuthica	0.00203	7.332126731	0.865229963
26	167	WP_126486177.1	ABC transporter substrate-binding protein	Serratia plymuthica	9.96E-05	7.341655103	0.865793978
59	76	WP_013814237.1	MULTISPECIES: glycine betaine/L-proline ABC transporter substrate-binding protein ProX	Serratia sp.	0.00034	7.507339623	0.875486063
20	71	WP_004950512.1	MULTISPECIES: ABC transporter substrate-binding protein	Serratia sp.	8.50E-05	7.624817875	0.882229475
93	36	WP_063203173.1	FKBP-type peptidyl-prolyl cis-trans isomerase	Serratia plymuthica	0.00105	7.944312129	0.900056299
15	201	WP_112290698.1	MULTISPECIES: sulfate ABC transporter substrate-binding protein	Rahnella sp.	5.52E-05	8.337536155	0.92103773
4	419	WP_110605874.1	glucans biosynthesis protein MdoG	Serratia plymuthica	7.48E-06	9.325084299	0.969652767
1	225	WP_062869220.1	glycerophosphodiester phosphodiesterase	Serratia plymuthica	9.82E-07	9.579085515	0.98132405
5	73	WP_015697963.1	glucan biosynthesis protein G	Rahnella aquatilis	1.50E-05	9.940460565	0.997406507
49	39	WP_113877246.1	FKBP-type peptidyl-prolyl cis-trans isomerase	Rahnella aquatilis	0.00025	10.51864281	1.021959708
85	485	WP_121020019.1	MULTISPECIES: insulinase family protein	Rahnella sp.	0.00088	11.42080863	1.057696855
13	371	WP_004943016.1	MULTISPECIES: YceI family protein	Serratia sp.	5.17E-05	12.49223956	1.096640304
21	384	WP_126485701.1	FKBP-type peptidyl-prolyl cis-trans isomerase	Serratia plymuthica	8.70E-05	13.34956587	1.125467143
29	759	WP_126484392.1	YgiW/YdeI family stress tolerance OB fold protein	Serratia plymuthica	0.00011	13.38550567	1.126634782
6	497	RMN19543.1	hypothetical protein ALQ63_00888	Serratia plymuthica	1.70E-05	14.66443248	1.16626526
12	300	WP_113876396.1	MULTISPECIES: serine endoprotease DegP	Rahnella sp.	4.57E-05	16.69979518	1.222711145
51	583	WP_112289032.1	MULTISPECIES: molecular chaperone OsmY	Rahnella sp.	0.00026	17.38745721	1.240236074
48	213	WP_013575568.1	MULTISPECIES: DUF1471 domain-containing protein	Rahnella sp.	0.00024	22.07861059	1.34397174
97	315	WP_113877533.1	glycine betaine/L-proline ABC transporter substrate-binding protein ProX	Rahnella aquatilis	0.00116	25.29775174	1.403081926

Table S4. Differential expression of proteins in Sp2 (*Serratia plymuthica*) treated with 0.4% NaCl vs Control.

Row	Index	Protein code	Protein identification	Reference organism	p-value	Fold Change	Log (Fold Change)
224	169	WP_113877445.1	FliC/FljB family flagellin	Rahnella aquatilis	0.00978	0.00241122	-2.617763184
45	1	WP_004945950.1	FliC/FljB family flagellin	Serratia plymuthica	0.0002	0.003444712	-2.462847032
18	588	WP_013575778.1	MULTISPECIES: FliC/FljB family flagellin	Rahnella sp.	5.13E-05	0.003530602	-2.452151194
48	376	WP_020439242.1	MULTISPECIES: FliC/FljB family flagellin	Serratia sp.	0.00024	0.004886339	-2.311016406
62	7	WP_004945953.1	MULTISPECIES: flagellar filament capping protein FliD	Serratia sp.	0.00033	0.008367229	-2.077418336
56	51	WP_004946072.1	MULTISPECIES: flagellar hook-filament junction protein FlgL	Serratia sp.	0.0003	0.009732362	-2.011781767
3	17	WP_122288670.1	flagellar hook-associated protein FlgK	Serratia plymuthica	2.41E-07	0.020905445	-1.679740587
200	550	WP_004946081.1	MULTISPECIES: flagellar basal-body rod protein FlgG	Serratia sp.	0.00613	0.026367569	-1.578929913
1	402	WP_073439293.1	flagellar hook assembly protein FlgD	Serratia plymuthica	3.46E-09	0.029276202	-1.533485262
146	124	WP_122289796.1	DUF2974 domain-containing protein	Serratia plymuthica	0.00242	0.04704924	-1.327447392
265	747	WP_037432192.1	phage major tail tube protein	Serratia plymuthica	0.01949	0.049618339	-1.304357778
183	139	WP_006323695.1	MULTISPECIES: phage major tail tube protein	Serratia sp.	0.00461	0.060931609	-1.215157353
52	411	WP_064798956.1	flagellar basal body rod protein FlgF	Serratia plymuthica	0.00028	0.062110821	-1.20683273
11	111	WP_013813492.1	MULTISPECIES: flagellar hook protein FlgE	Serratia sp.	2.51E-05	0.065475037	-1.183924248
32	357	WP_013812357.1	MULTISPECIES: DNA/RNA non-specific endonuclease	Serratia sp.	0.00012	0.065811467	-1.181698429
75	326	WP_047609302.1	chemotaxis protein CheW	Rahnella aquatilis	0.00056	0.083574178	-1.077927885
96	9	WP_062869988.1	hypothetical protein	Serratia plymuthica	0.0009	0.091187879	-1.040062887
148	30	AHY09490.1	hemolysin	Serratia plymuthica	0.00256	0.093045916	-1.031302685
141	134	WP_063201803.1	hypothetical protein	Serratia plymuthica	0.00231	0.10009042	-0.999607487
12	480	WP_037429949.1	hypothetical protein	Serratia plymuthica	2.93E-05	0.111779023	-0.95163969
285	354	WP_063198800.1	baseplate assembly protein	Serratia plymuthica	0.02433	0.112257632	-0.949784123
124	146	WP_006317741.1	MULTISPECIES: triose-phosphate isomerase	Serratia sp.	0.00175	0.11485185	-0.939862005
197	158	WP_122289835.1	matrixin family metalloprotease	Serratia plymuthica	0.00595	0.12427981	-0.90559942
275	637	WP_047606070.1	alcohol dehydrogenase AdhP	Rahnella aquatilis	0.02189	0.135028781	-0.869573652
215	131	AHY07005.1	hypothetical protein sch_10780	Serratia plymuthica	0.00772	0.136266714	-0.865610216
345	725	WP_004089944.1	MULTISPECIES: integration host factor subunit alpha	Enterobacterales	0.0488	0.144295566	-0.840747014
49	466	WP_112168192.1	MULTISPECIES: two-component system response regulator ArcA	Rahnella sp.	0.00024	0.145443237	-0.837306467
290	618	WP_006322046.1	MULTISPECIES: elongation factor P	Serratia sp.	0.02515	0.1605798	-0.794309087
280	505	WP_009639183.1	MULTISPECIES: 50S ribosomal protein L16	Yersiniaceae	0.02269	0.177241197	-0.751435327
235	294	WP_015699041.1	50S ribosomal protein L11	Rahnella aquatilis	0.01201	0.191365385	-0.718136617
153	557	WP_065506613.1	MULTISPECIES: formate dehydrogenase subunit alpha	Serratia sp.	0.0027	0.198060693	-0.703201707
253	238	WP_013577239.1	MULTISPECIES: 50S ribosomal protein L13	Yersiniaceae	0.01558	0.201275437	-0.696209221
226	388	WP_126480690.1	endopeptidase La	Serratia plymuthica	0.01028	0.202981689	-0.692543137
135	120	WP_122288902.1	matrixin family metalloprotease	Serratia plymuthica	0.00204	0.218265373	-0.661015159
121	314	WP_004951488.1	fimbrial protein	Serratia plymuthica	0.00169	0.219506808	-0.658552005
261	365	WP_015695926.1	S-ribosylhomocysteine lyase	Rahnella aquatilis	0.01908	0.229404286	-0.639398471
328	475	WP_126486128.1	ribosome-dependent GTPase TypA	Serratia plymuthica	0.04095	0.231086363	-0.636225683
144	160	WP_064798816.1	aldehyde dehydrogenase	Serratia plymuthica	0.00238	0.235098295	-0.628750521
147	194	WP_073440881.1	N-acetylmuramoyl-L-alanine amidase	Serratia plymuthica	0.00252	0.235566654	-0.627886186
98	254	WP_122289004.1	glycoside hydrolase family 68 protein	Serratia plymuthica	0.0009	0.237941148	-0.623530448
107	511	WP_004950476.1	MULTISPECIES: F0F1 ATP synthase subunit B	Serratia sp.	0.00119	0.247725278	-0.606029676
317	378	AGP46727.1	hypothetical protein M621_02115	Serratia plymuthica S13	0.03609	0.252529531	-0.597687828
116	228	WP_112286790.1	MULTISPECIES: transcription termination/antitermination protein NusA	Rahnella sp.	0.00153	0.26019989	-0.584692891
171	136	WP_126480368.1	recombinase RecA	Serratia plymuthica	0.00361	0.284331906	-0.546174404
302	399	WP_004946879.1	MULTISPECIES: DUF1440 domain-containing protein	Serratia sp.	0.02949	0.290132674	-0.537403359
287	302	WP_015698930.1	acetyl-CoA carboxylase biotin carboxylase subunit	Rahnella aquatilis	0.02442	0.299188717	-0.524054788
320	613	WP_112288147.1	MULTISPECIES: peptide deformylase	Rahnella sp.	0.03778	0.300969088	-0.521478108
223	152	WP_112291457.1	MULTISPECIES: recombinase RecA	Rahnella sp.	0.00964	0.326115255	-0.486628886
157	258	WP_013577593.1	MULTISPECIES: triose-phosphate isomerase	Rahnella sp.	0.00299	0.332597975	-0.4780804
100	22	WP_015697077.1	bifunctional acetaldehyde-CoA/alcohol dehydrogenase	Rahnella aquatilis	0.00099	0.334522542	-0.475574612
331	666	WP_073439042.1	NADH:flavin oxidoreductase/NADH oxidase	Serratia plymuthica	0.04115	0.335248769	-0.474632808
191	268	WP_013576134.1	MULTISPECIES: 50S ribosomal protein L20	Yersiniaceae	0.00525	0.341302198	-0.466860915
321	816	WP_126483270.1	PTS mannose transporter subunit IIAB	Serratia plymuthica	0.03784	0.345069812	-0.462093033
73	16	WP_014333640.1	chaperonin GroEL	Rahnella aquatilis	0.00053	0.357707669	-0.446471749
272	179	WP_004953623.1	MULTISPECIES: autonomous glycyl radical cofactor GrcA	Serratia sp.	0.02119	0.36487024	-0.437861558
256	726	WP_004933536.1	MULTISPECIES: ribosome assembly RNA-binding protein YhbY	Enterobacterales	0.01697	0.365652809	-0.436931086
279	113	WP_013575313.1	MULTISPECIES: pyruvate kinase	Rahnella sp.	0.02253	0.369361107	-0.432548836
281	218	WP_065506321.1	MULTISPECIES: pyruvate kinase	Serratia sp.	0.02282	0.375183868	-0.425755844
293	324	WP_112288177.1	MULTISPECIES: autonomous glycyl radical cofactor GrcA	Rahnella sp.	0.02612	0.376311873	-0.424452079
301	838	WP_126527624.1	lipoyl synthase	Serratia plymuthica	0.0291	0.391449431	-0.407324334
239	11	WP_013573670.1	MULTISPECIES: elongation factor Tu	Rahnella sp.	0.01222	0.409129155	-0.388139571
324	92	WP_005969574.1	MULTISPECIES: 30S ribosomal protein S7	Enterobacterales	0.0401	0.413468025	-0.38355807
335	770	WP_126527646.1	3-deoxy-7-phosphoheptulonate synthase AroG	Serratia plymuthica	0.04418	0.420734973	-0.375991387
22	416	WP_004951154.1	MULTISPECIES: 30S ribosomal protein S14	Serratia sp.	5.78E-05	0.422454365	-0.374220199
50	590	WP_056772785.1	MULTISPECIES: 30S ribosomal protein S16	Yersiniaceae	0.00025	0.430438101	-0.366089294
168	13	WP_006325792.1	bifunctional acetaldehyde-CoA/alcohol dehydrogenase	Serratia plymuthica	0.00341	0.435495093	-0.361016734
211	2	WP_013574828.1	MULTISPECIES: formate C-acetyltransferase	Rahnella sp.	0.0075	0.435656497	-0.360855805
286	10	WP_006324547.1	MULTISPECIES: formate C-acetyltransferase	Serratia sp.	0.02436	0.435750854	-0.360761753
154	738	WP_004093983.1	MULTISPECIES: 50S ribosomal protein L34	Enterobacterales	0.00275	0.435777745	-0.360734953
299	147	WP_112289290.1	MULTISPECIES: ribosome-dependent GTPase TypA	Rahnella sp.	0.02766	0.452316076	-0.344557977
242	742	WP_065505783.1	MULTISPECIES: DEAD/DEAH family ATP-dependent RNA helicase	Serratia sp.	0.0135	0.453124235	-0.34378271
93	657	WP_004932501.1	MULTISPECIES: 30S ribosomal protein S16	Enterobacterales	0.00086	0.455302688	-0.341699786
88	636	WP_004951128.1	MULTISPECIES: 30S ribosomal protein S11	Serratia sp.	0.00073	0.45951402	-0.337701233
133	185	WP_112290710.1	MULTISPECIES: cysteine synthase A	Rahnella sp.	0.00197	0.463173626	-0.334256178
260	98	WP_126527385.1	aspartate ammonia-lyase	Serratia plymuthica	0.01823	0.478213344	-0.320378309
244	265	WP_001144069.1	MULTISPECIES: 30S ribosomal protein S21	Bacteria	0.0136	0.479039876	-0.319628334
26	198	WP_014333606.1	MULTISPECIES: 30S ribosomal protein S4	Rahnella sp.	8.11E-05	0.485097738	-0.31417075
109	155	WP_013573668.1	MULTISPECIES: 30S ribosomal protein S12	Yersiniaceae	0.00122	0.494010779	-0.306263575
307	431	WP_113876187.1	MULTISPECIES: elongation factor 4	Rahnella sp.	0.03249	0.494230936	-0.306070074
254	844	WP_013814788.1	MULTISPECIES: large-conductance mechanosensitive channel protein MscL	Serratia sp.	0.01604	0.496722798	-0.303885907
243	53	WP_004951177.1	MULTISPECIES: 50S ribosomal protein L2	Serratia sp.	0.0136	0.500888256	-0.300259151
65	248	WP_004950372.1	MULTISPECIES: 50S ribosomal protein L19	Serratia sp.	0.00045	0.510015314	-0.292416783
219	224	WP_002221949.1	MULTISPECIES: transcription antiterminator/RNA stability regulator CspE	Enterobacterales	0.00887	0.512553155	-0.290261089
176	292	WP_062870646.1	pyruvate kinase PykF	Serratia plymuthica	0.00411	0.513067446	-0.28982554
203	90	WP_112288137.1	MULTISPECIES: 50S ribosomal protein L2	Rahnella sp.	0.00641	0.513592986	-0.289380916
199	441	WP_004947686.1	MULTISPECIES: PTS glucose transporter subunit IIA	Serratia sp.	0.00605	0.519228228	-0.284641705

Row	Index	Protein code	Protein identification	Reference organism	p-value	Fold Change	Log (Fold Change)
232	86	WP_004951125.1	MULTISPECIES: 30S ribosomal protein S4	Serratia sp.	0.01152	0.520862765	-0.283276688
259	48	WP_112290864.1	MULTISPECIES: glutamine--fructose-6-phosphate transaminase (isomerizing)	Rahnella sp.	0.0182	0.527335207	-0.277913232
131	116	WP_013573700.1	MULTISPECIES: 30S ribosomal protein S5	Yersiniaceae	0.00189	0.5300413	-0.275690289
137	591	WP_047612676.1	alpha,alpha-phosphotrehalase	Rahnella aquatilis	0.0022	0.531639174	-0.274383025
274	707	WP_013575246.1	MULTISPECIES: transcription antiterminator/RNA stability regulator CspE	Yersiniaceae	0.02143	0.535331654	-0.271377077
229	151	WP_004952226.1	MULTISPECIES: 30S ribosomal protein S2	Serratia sp.	0.01103	0.539599949	-0.2679281
312	270	WP_004943595.1	MULTISPECIES: 50S ribosomal protein L20	Serratia sp.	0.0334	0.546193906	-0.26265315
145	343	WP_004952787.1	MULTISPECIES: inorganic diphosphatase	Serratia sp.	0.0024	0.547551599	-0.261574949
295	607	WP_004951166.1	MULTISPECIES: 30S ribosomal protein S17	Serratia sp.	0.02647	0.548173316	-0.261082109
263	190	WP_120978375.1	type 1 fimbrial protein	Serratia plymuthica	0.01945	0.551576059	-0.258394592
291	82	WP_113876357.1	MULTISPECIES: pyruvate dehydrogenase complex dihydrolipoyllysine-residue acetyltransferase	Rahnella sp.	0.02544	0.557571273	-0.25369961
326	785	WP_126482255.1	RNA chaperone ProQ	Serratia plymuthica	0.04067	0.559676654	-0.252062809
267	672	WP_013574367.1	MULTISPECIES: nucleoside-diphosphate kinase	Yersiniaceae	0.0203	0.56831625	-0.245409926
158	677	WP_013573791.1	MULTISPECIES: inorganic diphosphatase	Rahnella sp.	0.00303	0.569952637	-0.244161233
247	231	WP_126527311.1	superoxide dismutase [Mn]	Serratia plymuthica	0.01475	0.578802516	-0.23746959
255	472	WP_004931195.1	MULTISPECIES: 50S ribosomal protein L28	Enterobacterales	0.01653	0.579434975	-0.236995294
89	27	WP_015695890.1	phosphopyruvate hydratase	Rahnella aquatilis	0.00079	0.582193825	-0.234932405
251	26	WP_095924049.1	MULTISPECIES: F0F1 ATP synthase subunit beta	Rahnella sp.	0.01519	0.587528554	-0.230971022
59	20	WP_004950026.1	MULTISPECIES: molecular chaperone DnaK	Serratia sp.	0.00031	0.587796453	-0.230773039
225	492	WP_002210155.1	MULTISPECIES: 30S ribosomal protein S18	Enterobacterales	0.00991	0.590077149	-0.229091203
190	28	WP_013577682.1	MULTISPECIES: F0F1 ATP synthase subunit alpha	Rahnella sp.	0.00518	0.591902256	-0.227750005
248	37	WP_002919219.1	MULTISPECIES: DNA-directed RNA polymerase subunit alpha	Enterobacterales	0.01493	0.598595783	-0.222866347
343	149	WP_004951140.1	MULTISPECIES: 30S ribosomal protein S5	Serratia sp.	0.04727	0.603686983	-0.219188188
230	253	WP_015698881.1	30S ribosomal protein S9	Rahnella aquatilis	0.01104	0.606680177	-0.217040195
167	31	WP_131638370.1	molecular chaperone DnaK	Rahnella aquatilis	0.00331	0.606728196	-0.217005822
245	443	WP_041416326.1	MULTISPECIES: ribosome recycling factor	Serratia sp.	0.01433	0.616357617	-0.210167233
339	148	WP_113877073.1	aspartate ammonia-lyase	Rahnella aquatilis	0.04582	0.616497212	-0.210068883
276	330	WP_004933561.1	MULTISPECIES: 50S ribosomal protein L21	Enterobacterales	0.02211	0.634553542	-0.197531727
187	43	WP_126480399.1	ATP-dependent chaperone ClpB	Serratia plymuthica	0.00484	0.637601778	-0.195450481
151	153	WP_037432618.1	asparagine--tRNA ligase	Serratia plymuthica	0.00266	0.656062085	-0.18305506
296	173	WP_006321797.1	MULTISPECIES: F0F1 ATP synthase subunit alpha	Serratia sp.	0.02662	0.659414539	-0.180841482
266	779	WP_004948431.1	MULTISPECIES: DUF883 domain-containing protein	Serratia sp.	0.01966	1.536463235	0.186522173
150	513	WP_004943122.1	MULTISPECIES: CopC domain-containing protein YobA	Serratia sp.	0.00261	1.54084068	0.187757736
334	512	WP_126485047.1	aminomethyl-transferring glycine dehydrogenase	Serratia plymuthica	0.04355	1.556544586	0.192161565
318	709	WP_126482406.1	tyrosine--tRNA ligase	Serratia plymuthica	0.03705	1.563182247	0.194009614
282	413	WP_064798650.1	MULTISPECIES: two-component system response regulator ArcA	Serratia sp.	0.02307	1.573059093	0.196745038
297	744	WP_073440145.1	adenylosuccinate lyase	Serratia plymuthica	0.0269	1.595448229	0.202882716
292	414	WP_015689898.1	MULTISPECIES: thiol peroxidase	Rahnella sp.	0.02547	1.611182796	0.207144816
305	732	WP_126527517.1	murein hydrolase activator NlpD	Serratia plymuthica	0.03164	1.618988571	0.209243783
222	271	WP_081481112.1	MULTISPECIES: transcription antiterminator/RNA stability regulator CspE	Rahnella sp.	0.00932	1.630555476	0.212335579
273	793	WP_126527372.1	uridine phosphorylase	Serratia plymuthica	0.02138	1.643531148	0.215777939
315	526	WP_062792216.1	MULTISPECIES: NAD(P)H-dependent oxidoreductase	Serratia sp.	0.03439	1.651172803	0.217792526
304	369	WP_063202862.1	L-asparaginase 2, partial	Serratia plymuthica	0.03115	1.709982084	0.23299156
237	74	WP_004944077.1	alpha-glucosidase	Serratia plymuthica	0.01219	1.735842833	0.239510401
214	486	WP_006325948.1	MULTISPECIES: peptidoglycan-binding protein LysM	Serratia sp.	0.0077	1.786416294	0.251982671
330	316	WP_013577396.1	MULTISPECIES: DNA-binding protein HU-alpha	Yersiniaceae	0.04114	1.807519105	0.257082896
284	554	WP_073440890.1	fused PTS fructose transporter subunit IIA/HPr protein	Serratia plymuthica	0.0235	1.852949746	0.267863641
108	199	WP_004943793.1	MULTISPECIES: glycine zipper 2TM domain-containing protein	Serratia sp.	0.0012	1.923644662	0.284124852
322	436	WP_013811573.1	MULTISPECIES: ribosome-associated translation inhibitor RaiA	Serratia sp.	0.03968	1.925534099	0.284551214
77	91	WP_062870397.1	porin OmpC	Serratia plymuthica	0.0006	1.946176227	0.289182163
288	286	WP_112198666.1	MULTISPECIES: ribonucleotide-diphosphate reductase subunit beta	Rahnella sp.	0.02443	1.987086199	0.298216707
332	575	WP_113877028.1	MULTISPECIES: acetyl-CoA carboxylase, carboxyltransferase subunit beta	Rahnella sp.	0.0424	2.014193945	0.304101286
314	77	WP_006323817.1	MULTISPECIES: ABC transporter substrate-binding protein	Serratia sp.	0.03422	2.032897012	0.308115378
336	753	WP_063201285.1	endonuclease	Serratia plymuthica	0.04488	2.112572527	0.324811628
333	830	WP_013814033.1	MULTISPECIES: nitrate reductase catalytic subunit NapA	Serratia sp.	0.0425	2.12409673	0.32717429
240	425	WP_073441200.1	capsule assembly Wzi family protein	Serratia plymuthica	0.01228	2.124827572	0.327323693
294	473	WP_126480386.1	DUF481 domain-containing protein	Serratia plymuthica	0.02615	2.137966009	0.330000796
289	528	WP_004949246.1	MULTISPECIES: preprotein translocase subunit YajC	Serratia sp.	0.02466	2.139055609	0.330222075
310	252	WP_013577542.1	MULTISPECIES: ketol-acid reductoisomerase	Rahnella sp.	0.03272	2.153573006	0.333159599
309	293	WP_020438738.1	MULTISPECIES: type 1 fimbrial protein	Serratia sp.	0.0327	2.165518968	0.335561992
341	841	WP_126483126.1	exodeoxyribonuclease III	Serratia plymuthica	0.04627	2.184753627	0.339402469
134	107	WP_013576426.1	MULTISPECIES: peptidoglycan-associated lipoprotein Pal	Rahnella sp.	0.00198	2.209027038	0.344201031
119	177	WP_013814922.1	MULTISPECIES: oligopeptidase A	Serratia sp.	0.00155	2.257356166	0.353600088
264	585	WP_126527586.1	hypothetical protein	Serratia plymuthica	0.01949	2.302625124	0.362223239
278	331	WP_015696270.1	glutamine-hydrolyzing GMP synthase	Rahnella aquatilis	0.02244	2.36302271	0.373467896
316	620	WP_013811512.1	MULTISPECIES: glutamate-1-semialdehyde 2,1-aminomutase	Serratia sp.	0.03459	2.390296767	0.378451824
338	325	WP_145917148.1	serine-type D-Ala-D-Ala carboxypeptidase	Serratia plymuthica	0.04559	2.403393016	0.380824795
300	545	WP_126485188.1	DUF2884 domain-containing protein	Serratia plymuthica	0.02807	2.456273961	0.390276804
308	532	WP_112291485.1	MULTISPECIES: nucleoside-specific channel-forming protein Tsx	Rahnella sp.	0.03266	2.4616921	0.391233732
122	706	WP_126484195.1	histidine ABC transporter substrate-binding protein HisJ	Serratia plymuthica	0.00169	2.489848454	0.396172914
327	600	WP_113876936.1	MULTISPECIES: glutathione-disulfide reductase	Rahnella sp.	0.04081	2.506897826	0.399136634
325	627	WP_126484855.1	murein transglycosylase A	Serratia plymuthica	0.0402	2.522097382	0.401761851
238	669	WP_073439459.1	ABC transporter substrate-binding protein	Serratia plymuthica	0.01221	2.552730113	0.407004902
180	856	WP_006323187.1	MULTISPECIES: entericidin A/B family lipoprotein	Serratia sp.	0.00447	2.576061594	0.410956243
78	313	WP_122289290.1	endolytic peptidoglycan transglycosylase RlpA	Serratia plymuthica	0.0006	2.635439716	0.420853086
227	332	WP_043912966.1	ferrichrome porin FhuA	Serratia plymuthica	0.01032	2.665565351	0.425789334
337	524	WP_006317468.1	MULTISPECIES: peptidyl-prolyl cis-trans isomerase	Serratia sp.	0.04542	2.770092334	0.442494245
233	696	WP_004949671.1	MULTISPECIES: glutamine--tRNA ligase	Serratia sp.	0.01154	2.774332222	0.443158466
164	225	WP_062869220.1	glycerophosphodiester phosphodiesterase	Serratia plymuthica	0.00329	2.784058807	0.444678405
192	109	WP_013814057.1	MULTISPECIES: outer membrane protein assembly factor BamC	Serratia sp.	0.00543	2.809616964	0.448647116
178	517	WP_037037332.1	MULTISPECIES: ACP S-malonyltransferase	Rahnella sp.	0.00424	2.820850002	0.450379993
105	363	WP_015696434.1	amidophosphoribosyltransferase	Rahnella aquatilis	0.00112	2.841909836	0.453610295
126	66	WP_073439813.1	aldose 1-epimerase family protein	Serratia plymuthica	0.00184	2.849572153	0.454779658
204	122	WP_112289500.1	MULTISPECIES: ribonucleoside-diphosphate reductase subunit alpha	Rahnella sp.	0.00645	2.862303257	0.456715645
206	114	WP_126485974.1	ketol-acid reductoisomerase	Serratia plymuthica	0.00666	2.874541995	0.458568658
136	577	WP_122288861.1	DUF3313 domain-containing protein	Serratia plymuthica	0.00215	2.931462276	0.46708431
268	6	WP_063200275.1	pitrilysin	Serratia plymuthica	0.02048	2.987017894	0.475237824

Row	Index	Protein code	Protein identification	Reference organism	p-value	Fold Change	Log (Fold Change)
160	741	WP_126484412.1	transaldolase	Serratia plymuthica	0.00311	2.991263854	0.475854723
270	824	WP_126480584.1	DUF2076 domain-containing protein	Serratia plymuthica	0.02074	3.027976568	0.48115251
249	135	WP_122288872.1	S9 family peptidase	Serratia plymuthica	0.01497	3.068592189	0.486939175
181	542	WP_126482196.1	nucleoside-specific channel-forming protein Tsx	Serratia plymuthica	0.00452	3.075684728	0.487941816
303	68	WP_112286943.1	MULTISPECIES: cell division protein FtsZ	Rahnella sp.	0.03062	3.089456851	0.489882134
271	180	WP_073440048.1	catalase	Serratia plymuthica	0.02091	3.123961564	0.494705682
115	604	WP_004942697.1	MULTISPECIES: L,D-transpeptidase	Serratia sp.	0.00142	3.131163223	0.495705707
36	226	WP_063201917.1	cystine ABC transporter substrate-binding protein	Serratia plymuthica	0.00014	3.177897073	0.502139827
55	748	WP_063200032.1	betaine-aldehyde dehydrogenase	Serratia plymuthica	0.00029	3.268735745	0.514379812
209	403	WP_062871046.1	MipA/OmpV family protein	Serratia plymuthica	0.00702	3.271939577	0.514805275
163	58	WP_122288888.1	penicillin-binding protein activator	Serratia plymuthica	0.00325	3.272335718	0.514857853
27	311	WP_004942530.1	MULTISPECIES: arginine ABC transporter substrate-binding protein	Serratia sp.	8.14E-05	3.277435414	0.515534142
262	222	WP_073441219.1	N-acetylmuramoyl-L-alanine amidase	Serratia plymuthica	0.01933	3.288536507	0.517002667
166	297	WP_062791335.1	murein hydrolase activator NlpD	Serratia plymuthica	0.0033	3.290016913	0.517198131
58	782	WP_073440375.1	peptidase inhibitor I78 family protein	Serratia plymuthica	0.0003	3.324351595	0.52170695
155	320	WP_047606344.1	aminopeptidase N	Rahnella aquatilis	0.00278	3.331605281	0.522653542
269	715	WP_113876926.1	peptidyl-dipeptidase Dcp	Rahnella aquatilis	0.02066	3.377280678	0.528567155
57	94	WP_013814266.1	MULTISPECIES: outer membrane protein assembly factor BamA	Serratia sp.	0.0003	3.384643748	0.529512964
221	220	WP_112289028.1	MULTISPECIES: thymidine phosphorylase	Rahnella sp.	0.00914	3.42218428	0.534303392
189	631	WP_063196854.1	osmotically-inducible lipoprotein OsmE	Serratia plymuthica	0.00515	3.431747075	0.535515272
130	457	WP_110605936.1	hypothetical protein	Serratia plymuthica	0.00187	3.492174567	0.543095945
186	140	WP_013811470.1	MULTISPECIES: peptidylprolyl isomerase SurA	Serratia sp.	0.0048	3.516999249	0.546172276
95	508	WP_004943172.1	hypothetical protein	Serratia plymuthica	0.00089	3.528838346	0.547631764
91	612	WP_047612859.1	pitrilysin	Rahnella aquatilis	0.00082	3.537416532	0.548686201
123	189	WP_006323429.1	MULTISPECIES: L,D-transpeptidase family protein	Serratia sp.	0.00172	3.563478481	0.551874141
104	215	WP_006322974.1	ABC transporter substrate-binding protein	Serratia plymuthica	0.00109	3.583692584	0.554330748
177	685	WP_122289575.1	Fe2+-enterobactin ABC transporter substrate-binding protein	Serratia plymuthica	0.00423	3.584560802	0.554435951
195	834	WP_126527408.1	N-acetyltransferase	Serratia plymuthica	0.00558	3.650214871	0.56231843
198	409	WP_004949342.1	MULTISPECIES: hypothetical protein	Serratia sp.	0.00601	3.652117965	0.562544797
110	829	WP_073440327.1	MULTISPECIES: ribosome maturation factor RimP	Serratia sp.	0.00123	3.713822029	0.569821088
236	583	WP_112289032.1	MULTISPECIES: molecular chaperone OsmY	Rahnella sp.	0.01209	3.862654539	0.586885868
196	750	WP_037432055.1	uroporphyrinogen-III C-methyltransferase	Serratia plymuthica	0.00562	3.872032702	0.587939017
4	504	WP_004945257.1	MULTISPECIES: BON domain-containing protein	Serratia sp.	1.40E-06	3.890673826	0.590024823
20	664	WP_073440218.1	thioredoxin domain-containing protein	Serratia plymuthica	5.21E-05	3.922946742	0.593612412
101	702	WP_041417326.1	methionine ABC transporter substrate-binding protein	Serratia plymuthica	0.00101	3.932409043	0.594658686
76	275	WP_006324520.1	arginine ABC transporter substrate-binding protein	Serratia plymuthica	0.00057	3.977959286	0.599660334
188	133	WP_015696692.1	porin OmpA	Rahnella aquatilis	0.00504	3.984142459	0.600334859
212	852	WP_073440264.1	P1 family peptidase	Serratia plymuthica	0.00758	4.00871568	0.603005255
127	580	WP_122289187.1	LPS assembly protein LptD	Serratia plymuthica	0.00184	4.028014771	0.605091054
74	267	WP_122288565.1	bifunctional glucose-1-phosphatase/inositol phosphatase	Serratia plymuthica	0.00054	4.034718315	0.60581322
139	183	WP_063201108.1	oligopeptide ABC transporter substrate-binding protein OppA	Serratia plymuthica	0.00228	4.036752358	0.606032108
156	84	WP_126482993.1	oligopeptide ABC transporter substrate-binding protein OppA	Serratia plymuthica	0.00286	4.051134078	0.607576617
246	175	WP_062870706.1	class C beta-lactamase	Serratia plymuthica	0.01434	4.061174123	0.60865161
87	191	WP_004952064.1	MULTISPECIES: outer membrane protein assembly factor BamB	Serratia sp.	0.00072	4.087892825	0.611499501
205	596	WP_073440841.1	glutamine-hydrolyzing GMP synthase	Serratia plymuthica	0.00647	4.093675959	0.612113462
19	50	WP_047607744.1	cystine ABC transporter substrate-binding protein	Rahnella aquatilis	5.19E-05	4.108997371	0.613735863
31	167	WP_126486177.1	ABC transporter substrate-binding protein	Serratia plymuthica	9.92E-05	4.113974265	0.614261571
128	329	WP_004949394.1	MULTISPECIES: bifunctional UDP-sugar hydrolase/5'-nucleotidase	Serratia sp.	0.00185	4.165095166	0.619624929
132	362	WP_126481301.1	glutamine ABC transporter substrate-binding protein GlnH	Serratia plymuthica	0.00189	4.293372345	0.632798555
175	783	WP_062870578.1	hypothetical protein	Serratia plymuthica	0.00397	4.304847357	0.633957757
217	256	WP_006328507.1	MULTISPECIES: peptidylprolyl isomerase A	Serratia sp.	0.00813	4.329657128	0.636453505
25	12	WP_006324584.1	porin OmpA	Serratia plymuthica	7.25E-05	4.346752532	0.638164916
64	367	WP_006322224.1	hypothetical protein	Serratia plymuthica	0.00042	4.378094872	0.641285168
311	802	WP_063198203.1	hypothetical protein	Serratia plymuthica	0.03277	4.383350374	0.641806186
120	689	WP_015697849.1	carboxy terminal-processing peptidase	Rahnella aquatilis	0.00158	4.393475874	0.642808246
42	633	WP_004949970.1	MULTISPECIES: OmpA family protein	Serratia sp.	0.00019	4.425110758	0.645924145
210	582	WP_073999785.1	multifunctional acyl-CoA thioesterase I/protease I/lysophospholipase L1	Serratia plymuthica	0.00712	4.42860929	0.646267367
201	448	WP_020439668.1	glycine betaine/L-proline ABC transporter ATP-binding protein ProV	Serratia plymuthica	0.00617	4.680707461	0.670311499
97	80	WP_006328168.1	outer membrane channel protein TolC	Serratia plymuthica	0.0009	4.70265873	0.672343463
80	193	WP_122288956.1	L,D-transpeptidase family protein	Serratia plymuthica	0.00061	4.708365847	0.672870201
140	235	WP_122289073.1	multicopper oxidase CueO	Serratia plymuthica	0.0023	4.76044217	0.677647294
90	282	WP_065505971.1	MULTISPECIES: cell division protein CpoB	Serratia sp.	0.00082	4.832876651	0.684205711
29	581	WP_111738524.1	outer membrane channel protein TolC	Serratia plymuthica	9.12E-05	4.949766466	0.694584709
165	728	WP_073440858.1	DUF1090 domain-containing protein	Serratia plymuthica	0.00329	4.963085772	0.695751781
194	663	WP_126480797.1	co-chaperone YbbN	Serratia plymuthica	0.00553	4.979904148	0.697220984
68	360	WP_112288331.1	MULTISPECIES: outer membrane protein assembly factor BamA	Rahnella sp.	0.00049	4.997285468	0.698734159
169	561	WP_122289113.1	oligopeptide ABC transporter substrate-binding protein OppA	Serratia plymuthica	0.00345	5.018554478	0.700578643
103	71	WP_004950512.1	MULTISPECIES: ABC transporter substrate-binding protein	Serratia sp.	0.00109	5.044140765	0.702787198
85	145	WP_073439860.1	carboxy terminal-processing peptidase	Serratia plymuthica	0.00069	5.054789889	0.703703108
162	385	WP_004949986.1	protein CreA	Serratia plymuthica	0.0032	5.120243887	0.709290648
185	243	WP_047609790.1	murein transglycosylase	Rahnella aquatilis	0.00464	5.13031446	0.710143986
83	698	WP_126486190.1	NAD(P)H-dependent oxidoreductase	Serratia plymuthica	0.00064	5.17321183	0.713760262
39	522	WP_065506637.1	MULTISPECIES: divisome-associated lipoprotein YraP	Serratia sp.	0.00017	5.197880879	0.715826322
117	629	WP_004945038.1	MULTISPECIES: 2-hydroxyacid dehydrogenase	Serratia sp.	0.00153	5.209832366	0.716823749
33	54	WP_015698105.1	Tol-Pal system protein TolB	Rahnella aquatilis	0.00013	5.246720291	0.719887912
258	674	WP_064798698.1	3',5'-cyclic-nucleotide phosphodiesterase	Serratia plymuthica	0.01769	5.279804439	0.722617837
193	787	WP_126528909.1	autoinducer 2 ABC transporter substrate-binding protein	Serratia plymuthica	0.00545	5.321336519	0.726020724
129	483	WP_113877622.1	MULTISPECIES: sulfate ABC transporter substrate-binding protein	Rahnella sp.	0.00185	5.337523292	0.727339783
111	380	WP_112287456.1	MULTISPECIES: ABC transporter substrate-binding protein	Rahnella sp.	0.00128	5.378557423	0.73066581
86	3	WP_063198645.1	murein transglycosylase	Serratia plymuthica	0.0007	5.401787852	0.732537524
30	174	WP_122289663.1	thiol:disulfide interchange protein DsbA	Serratia plymuthica	9.65E-05	5.436655079	0.735331781
213	662	WP_112288890.1	MULTISPECIES: transketolase	Rahnella sp.	0.00761	5.505347725	0.740784755
38	106	WP_004942247.1	MULTISPECIES: outer membrane protein OmpX	Serratia sp.	0.00017	5.636147282	0.750982333
82	497	RMN19543.1	hypothetical protein ALQ63_00888	Serratia plymuthica	0.00064	5.64073456	0.751335663
112	622	WP_063197910.1	LPS assembly lipoprotein LptE	Serratia plymuthica	0.00131	5.644273676	0.751608063
113	455	WP_122289110.1	NAD(P)H nitroreductase	Serratia plymuthica	0.00132	5.725114064	0.757784144
138	101	WP_122288887.1	serine endoprotease DegQ	Serratia plymuthica	0.00222	6.036711615	0.780800429
9	652	WP_126528657.1	YgdI/YgdR family lipoprotein	Serratia plymuthica	1.38E-05	6.125022545	0.787107692

Row	Index	Protein code	Protein identification	Reference organism	p-value	Fold Change	Log (Fold Change)
202	266	WP_004948518.1	MULTISPECIES: phospholipid-binding protein MlaC	Serratia sp.	0.00633	6.125597037	0.787148424
84	389	WP_073440018.1	beta-glucosidase BglX	Serratia plymuthica	0.00067	6.135973572	0.78788348
125	718	WP_113877411.1	MULTISPECIES: murein hydrolase activator NlpD	Rahnella sp.	0.0018	6.183115326	0.791207347
21	251	WP_013812141.1	MULTISPECIES: fimbria/pilus periplasmic chaperone	Serratia sp.	5.38E-05	6.203088729	0.792607993
79	714	WP_126484642.1	two-component system QseEF-associated lipoprotein QseG	Serratia plymuthica	0.0006	6.256348451	0.796320929
149	488	RMN17366.1	hypothetical protein ALQ63_02794	Serratia plymuthica	0.00257	6.284137499	0.798245679
37	574	WP_006323718.1	MULTISPECIES: outer membrane protein assembly factor BamD	Serratia sp.	0.00015	6.381012661	0.804889606
173	568	WP_013811875.1	MULTISPECIES: LPS assembly lipoprotein LptE	Serratia sp.	0.00376	6.476574538	0.811345368
71	201	WP_112290698.1	MULTISPECIES: sulfate ABC transporter substrate-binding protein	Rahnella sp.	0.00051	6.591565975	0.818988603
51	534	WP_062868083.1	porin	Serratia plymuthica	0.00026	6.62563516	0.821227518
43	668	WP_013813948.1	MULTISPECIES: phospholipid-binding lipoprotein MlaA	Serratia sp.	0.00019	6.800748397	0.832556708
10	207	WP_004952521.1	MULTISPECIES: oxidative stress defense protein	Serratia sp.	2.34E-05	6.903136677	0.839046472
40	38	WP_006326771.1	porin OmpC	Serratia plymuthica	0.00019	6.908777779	0.839401224
184	211	WP_112289187.1	MULTISPECIES: Tol-Pal system protein TolB	Rahnella sp.	0.00461	6.967309906	0.843065128
23	49	WP_004949773.1	MULTISPECIES: Tol-Pal system protein TolB	Serratia sp.	6.00E-05	6.978868086	0.843784989
16	104	WP_004944994.1	DUF1471 domain-containing protein	Serratia plymuthica	5.08E-05	7.030992123	0.847016611
142	250	WP_004942614.1	MULTISPECIES: outer membrane lipoprotein chaperone LolA	Serratia sp.	0.00233	7.0423015	0.847714614
6	721	WP_126480972.1	Tol-Pal system protein TolB	Serratia plymuthica	3.65E-06	7.141716013	0.853802577
15	630	WP_112287581.1	MULTISPECIES: outer membrane protein assembly factor BamC	Rahnella sp.	4.59E-05	7.381006408	0.868115582
159	649	WP_013577556.1	MULTISPECIES: ABC transporter substrate-binding protein	Rahnella sp.	0.00307	7.482269603	0.874033353
172	437	WP_006328270.1	MULTISPECIES: lipopolysaccharide ABC transporter substrate-binding protein LptA	Serratia sp.	0.00364	7.689392446	0.885892027
13	244	WP_004952410.1	MULTISPECIES: bifunctional protein-disulfide isomerase/oxidoreductase DsbC	Serratia sp.	3.20E-05	8.021905031	0.904277516
66	110	WP_006328854.1	sulfate ABC transporter substrate-binding protein	Serratia plymuthica	0.00048	8.042700305	0.905401886
47	142	WP_062790903.1	MULTISPECIES: ABC transporter substrate-binding protein	Serratia sp.	0.00024	8.327147677	0.920496267
114	15	WP_004942332.1	glutathione ABC transporter substrate-binding protein GsiB	Serratia plymuthica	0.00136	8.385614917	0.923534915
94	64	WP_006323248.1	bifunctional 2',3'-cyclic-nucleotide 2'-phosphodiesterase/3'-nucleotidase	Serratia plymuthica	0.00087	8.97895138	0.95322562
63	418	WP_126124940.1	iron uptake system protein EfeO	Rahnella aquatilis	0.00037	9.158153311	0.96180791
99	97	WP_004950859.1	MULTISPECIES: insulinase family protein	Serratia sp.	0.00091	9.392893244	0.972799386
102	371	WP_004943016.1	MULTISPECIES: YceI family protein	Serratia sp.	0.00108	9.610932742	0.982765538
182	467	WP_122288686.1	acid phosphatase	Serratia plymuthica	0.00457	9.669461334	0.985402281
60	632	WP_131638597.1	phospholipid-binding protein MlaC	Rahnella aquatilis	0.00031	9.678725003	0.985818151
72	708	WP_112288332.1	MULTISPECIES: molecular chaperone Skp	Rahnella sp.	0.00051	10.01749002	1.000758918
44	719	WP_037432390.1	molybdate ABC transporter substrate-binding protein	Serratia plymuthica	0.00019	10.10882072	1.004700494
118	355	WP_064798747.1	polysaccharide export protein	Serratia plymuthica	0.00154	10.11376017	1.004912651
170	762	WP_126527395.1	N-acetylmuramoyl-L-alanine amidase AmiB	Serratia plymuthica	0.00352	10.15971548	1.006881546
53	551	WP_126486390.1	zinc ABC transporter substrate-binding protein ZnuA	Serratia plymuthica	0.00029	10.48579293	1.020601277
14	765	WP_126527482.1	thiamine ABC transporter substrate binding subunit	Serratia plymuthica	3.71E-05	10.55771217	1.023569818
81	605	WP_073439559.1	M48 family metallopeptidase	Serratia plymuthica	0.00061	10.80287224	1.03353924
231	283	WP_113878243.1	MULTISPECIES: oxidative stress defense protein	Rahnella sp.	0.01148	10.88321786	1.036757323
41	445	WP_006324721.1	MULTISPECIES: penicillin-binding protein activator LpoB	Serratia sp.	0.00019	12.10328532	1.082903271
92	759	WP_126484392.1	YgiW/YdeI family stress tolerance OB fold protein	Serratia plymuthica	0.00083	12.83015523	1.108231911
34	162	WP_063199821.1	serine protease inhibitor ecotin	Serratia plymuthica	0.00013	12.96815822	1.112878301
174	356	WP_004952213.1	MULTISPECIES: molecular chaperone Skp	Serratia sp.	0.00384	13.70948267	1.137021067
24	257	WP_013576150.1	MULTISPECIES: carboxy terminal-processing peptidase	Rahnella sp.	6.97E-05	13.80712906	1.140103384
234	289	WP_013573626.1	MULTISPECIES: peptidylprolyl isomerase A	Rahnella sp.	0.01155	14.1272829	1.150058642
5	36	WP_063203173.1	FKBP-type peptidyl-prolyl cis-trans isomerase	Serratia plymuthica	1.94E-06	14.32480427	1.156088697
208	46	WP_126527496.1	serine endoprotease DegP	Serratia plymuthica	0.00693	14.79160285	1.170015238
257	809	WP_126481690.1	porin OmpA	Serratia plymuthica	0.01751	16.04628403	1.205374475
7	5	WP_086016609.1	MULTISPECIES: glucans biosynthesis protein MdoG	Serratia sp.	4.09E-06	16.43149673	1.215677125
54	566	WP_004953231.1	MULTISPECIES: periplasmic heavy metal sensor	Serratia sp.	0.00029	16.44987141	1.216162507
35	700	WP_047608839.1	LPS assembly lipoprotein LptE	Rahnella aquatilis	0.00013	17.09842115	1.23295601
179	300	WP_113876396.1	MULTISPECIES: serine endoprotease DegP	Rahnella sp.	0.00426	18.44922621	1.265978156
207	391	WP_119262138.1	Do family serine endopeptidase	Rahnella aquatilis	0.00673	21.12496564	1.324796011
152	675	WP_073440332.1	glutathione ABC transporter substrate-binding protein GsiB	Serratia plymuthica	0.00267	21.29586745	1.328295335
2	396	WP_006320904.1	serine protease inhibitor ecotin	Serratia plymuthica	4.23E-08	21.98383196	1.342103396
70	76	WP_013814237.1	MULTISPECIES: glycine betaine/L-proline ABC transporter substrate-binding protein ProX	Serratia sp.	0.0005	27.99334481	1.447054794
46	39	WP_113877246.1	FKBP-type peptidyl-prolyl cis-trans isomerase	Rahnella aquatilis	0.00024	30.93814185	1.490494226
17	73	WP_015697963.1	glucan biosynthesis protein G	Rahnella aquatilis	5.10E-05	32.86184741	1.516691975
61	384	WP_126485701.1	FKBP-type peptidyl-prolyl cis-trans isomerase	Serratia plymuthica	0.00032	37.56282577	1.574758256
28	377	WP_126485956.1	ATP-independent periplasmic protein-refolding chaperone	Serratia plymuthica	8.86E-05	40.39854708	1.606365746
8	419	WP_110605874.1	glucans biosynthesis protein MdoG	Serratia plymuthica	5.29E-06	54.67386872	1.737779806
69	442	WP_112289283.1	MULTISPECIES: thiol:disulfide interchange protein DsbA	Rahnella sp.	0.00049	127.1569922	2.104340246

Table S5. GO terms of proteins overexpressed both in Ra4 (*Rahnella aquatilis*) and in Sp2 (*Serratia plymuthica*) treated with 0.4% NaCl vs Control.

GO Term	Description	P-value_Rahnella	P-value_Serratia
GO:0000099	sulfur amino acid transmembrane transporter activity	0.006548134	0.02537498
GO:0000270	peptidoglycan metabolic process	0.006752674	0.00286326
GO:0000413	protein peptidyl-prolyl isomerization	0.02157472	0.04108586
GO:0003333	amino acid transmembrane transport	0.0352436	0.01416171
GO:0003755	peptidyl-prolyl cis-trans isomerase activity	0.02157472	0.04108586
GO:0003756	protein disulfide isomerase activity	0.008688366	0.01483782
GO:0004112	cyclic-nucleotide phosphodiesterase activity	0.006548134	0.02537498
GO:0004175	endopeptidase activity	0.001542671	0.00037194
GO:0004180	carboxypeptidase activity	0.04103834	0.02996041
GO:0004252	serine-type endopeptidase activity	0.01431698	0.01483782
GO:0005215	transporter activity	0.002096246	0.002192641
GO:0006022	aminoglycan metabolic process	0.006752674	0.00286326
GO:0006073	cellular glucan metabolic process	0.004614553	0.03125707
GO:0006457	protein folding	4.33E-05	4.23E-05
GO:0006508	proteolysis	0.005070263	0.001565315
GO:0006791	sulfur utilization	0.006548134	0.02537498
GO:0006810	transport	9.54E-05	1.93E-09
GO:0006811	ion transport	0.006810814	9.47E-06
GO:0006820	anion transport	0.009891719	2.24E-05
GO:0006970	response to osmotic stress	3.09E-05	2.87E-05
GO:0006972	hyperosmotic response	0.000577283	0.003660424
GO:0008081	phosphoric diester hydrolase activity	0.000521021	0.01416171
GO:0008104	protein localization	0.004867352	0.000181888
GO:0008233	peptidase activity	0.005070263	0.001565315
GO:0008234	cysteine-type peptidase activity	0.001961127	0.004014615
GO:0008236	serine-type peptidase activity	0.000669939	0.004872788
GO:0008272	sulfate transport	0.001961127	0.03125707
GO:0008933	lytic transglycosylase activity	0.01860236	0.01416171
GO:0009057	macromolecule catabolic process	0.000775487	0.01407373
GO:0009250	glucan biosynthetic process	0.004614553	0.03125707
GO:0009273	peptidoglycan-based cell wall biogenesis	0.01218291	0.004872788
GO:0009274	peptidoglycan-based cell wall	0.006548134	0.02537498
GO:0009279	cell outer membrane	0.03105572	4.07E-13
GO:0009719	response to endogenous stimulus	4.10E-05	0.00063225
GO:0010243	response to organonitrogen compound	0.000540739	0.0027648
GO:0015031	protein transport	0.006752674	0.007509891
GO:0015171	amino acid transmembrane transporter activity	0.006548134	0.02537498
GO:0015179	L-amino acid transmembrane transporter activity	0.006548134	0.02537498
GO:0015184	L-cystine transmembrane transporter activity	0.006548134	0.02537498
GO:0015695	organic cation transport	0.008688366	0.03125707
GO:0015696	ammonium transport	0.008688366	0.03125707
GO:0015697	quaternary ammonium group transport	0.004614553	0.01416171
GO:0015698	inorganic anion transport	0.004614553	0.01483782
GO:0015804	neutral amino acid transport	0.006548134	0.02537498
GO:0015811	L-cystine transport	0.006548134	0.02537498
GO:0015833	peptide transport	0.001542671	2.92E-05
GO:0015838	amino-acid betaine transport	0.004614553	0.01416171
GO:0015893	drug transport	0.02781516	0.000735212
GO:0016755	transferase activity, transferring amino-acyl groups	0.001961127	0.004014615
GO:0016807	cysteine-type carboxypeptidase activity	0.001961127	0.004014615
GO:0016859	cis-trans isomerase activity	0.02157472	0.04108586
GO:0016864	intramolecular oxidoreductase activity, transposing S-S bonds	0.008688366	0.01483782
GO:0017038	protein import	4.10E-05	0.0027648
GO:0017171	serine hydrolase activity	0.000669939	0.004872788
GO:0018104	peptidoglycan-protein cross-linking	0.01860236	0.02537498
GO:0018208	peptidyl-proline modification	0.02157472	0.04108586
GO:0019534	toxin transmembrane transporter activity	4.10E-05	0.00063225

GO Term	Description	P-value_Rahnella	P-value_Serratia
GO:0019867	outer membrane	0.03105572	4.07E-13
GO:0022857	transmembrane transporter activity	0.02281951	0.0104204
GO:0030163	protein catabolic process	0.006752674	0.002421263
GO:0030203	glycosaminoglycan metabolic process	0.006752674	0.00286326
GO:0030288	outer membrane-bounded periplasmic space	2.11E-26	1.69E-35
GO:0030313	cell envelope	4.64E-26	1.79E-48
GO:0031460	glycine betaine transport	0.004614553	0.01416171
GO:0031647	regulation of protein stability	0.0352436	0.01416171
GO:0031975	envelope	4.64E-26	1.79E-48
GO:0032153	cell division site	0.000262603	0.001894049
GO:0033036	macromolecule localization	0.025298	6.69E-07
GO:0042493	response to drug	0.03408151	0.008961024
GO:0042546	cell wall biogenesis	0.01218291	0.004872788
GO:0042578	phosphoric ester hydrolase activity	0.03048673	0.01251551
GO:0042597	periplasmic space	9.79E-32	1.37E-40
GO:0042886	amide transport	0.001542671	2.92E-05
GO:0042891	obsolete antibiotic transport	0.000540739	0.02601521
GO:0043164	Gram-negative-bacterium-type cell wall biogenesis	0.001961127	0.004014615
GO:0043199	sulfate binding	0.001961127	0.03125707
GO:0043213	bacteriocin transport	4.10E-05	0.0027648
GO:0044036	cell wall macromolecule metabolic process	0.02171932	0.01107193
GO:0044042	glucan metabolic process	0.000540739	0.007259702
GO:0044257	cellular protein catabolic process	0.04103834	0.02001884
GO:0044462	obsolete external encapsulating structure part	0.03105572	4.07E-13
GO:0045184	establishment of protein localization	0.004867352	0.000105546
GO:0046677	response to antibiotic	0.009891719	0.02672135
GO:0046678	response to bacteriocin	4.10E-05	0.00063225
GO:0050821	protein stabilization	0.0352436	0.01416171
GO:0051179	localization	0.000604922	5.28E-08
GO:0051234	establishment of localization	7.87E-05	5.20E-11
GO:0051273	beta-glucan metabolic process	0.000521021	0.004014615
GO:0051274	beta-glucan biosynthetic process	0.000521021	0.004014615
GO:0051603	proteolysis involved in cellular protein catabolic process	0.03048673	0.007176907
GO:0055085	transmembrane transport	0.03906357	0.01088411
GO:0061077	chaperone-mediated protein folding	0.009656712	0.001183254
GO:0070004	cysteine-type exopeptidase activity	0.001961127	0.004014615
GO:0070011	peptidase activity, acting on L-amino acid peptides	0.001255046	0.001042228
GO:0071236	cellular response to antibiotic	0.000192177	0.0027648
GO:0071237	cellular response to bacteriocin	4.10E-05	0.00063225
GO:0071310	cellular response to organic substance	0.00118355	0.01483782
GO:0071417	cellular response to organonitrogen compound	4.10E-05	0.00063225
GO:0071495	cellular response to endogenous stimulus	4.10E-05	0.00063225
GO:0071554	cell wall organization or biogenesis	0.02171932	0.01571855
GO:0071702	organic substance transport	0.000213493	3.02E-08
GO:0071705	nitrogen compound transport	3.89E-06	4.06E-09
GO:0071972	peptidoglycan L,D-transpeptidase activity	0.001961127	0.004014615
GO:0072337	modified amino acid transport	5.86E-05	0.00051735
GO:0072348	sulfur compound transport	0.000308292	0.001838301
GO:0072349	modified amino acid transmembrane transporter activity	0.006548134	0.02537498
GO:0140096	catalytic activity, acting on a protein	9.00E-05	0.000300407
GO:1901652	response to peptide	4.10E-05	0.00063225
GO:1901653	cellular response to peptide	4.10E-05	0.00063225
GO:1901682	sulfur compound transmembrane transporter activity	0.006548134	0.02537498
GO:1901698	response to nitrogen compound	0.000540739	0.0027648
GO:1901699	cellular response to nitrogen compound	4.10E-05	0.00063225
GO:1901700	response to oxygen-containing compound	0.000280749	0.02160075

GO Term	Description	P-value_Rahnella	P-value_Serratia
GO:1901701	cellular response to oxygen-containing compound	3.93E-05	0.006100628
GO:1901998	toxin transport	4.10E-05	0.00063225
GO:1902475	L-alpha-amino acid transmembrane transport	0.0352436	0.01416171

Table S6. GO terms of proteins overexpressed only in Ra4 (*Rahnella aquatilis*) treated with 0.4% NaCl vs Control.

GO Term	Description	P-value (Rahnella)
GO:0000101	sulfur amino acid transport	0.0352436
GO:0000302	response to reactive oxygen species	0.02157472
GO:0000920	septum digestion after cytokinesis	0.0352436
GO:0001896	autolysis	0.01860236
GO:0004185	serine-type carboxypeptidase activity	0.01431698
GO:0005342	organic acid transmembrane transporter activity	0.0352436
GO:0005976	polysaccharide metabolic process	0.02171932
GO:0006026	aminoglycan catabolic process	0.01860236
GO:0006027	glycosaminoglycan catabolic process	0.01860236
GO:0008514	organic anion transmembrane transporter activity	0.0352436
GO:0008932	lytic endotransglycosylase activity	0.006548134
GO:0009253	peptidoglycan catabolic process	0.01860236
GO:0009628	response to abiotic stimulus	0.02308372
GO:0019904	protein domain specific binding	0.03483599
GO:0034605	cellular response to heat	0.01860236
GO:0042026	protein refolding	0.008688366
GO:0043085	positive regulation of catalytic activity	0.04103834
GO:0044093	positive regulation of molecular function	0.04103834
GO:0046943	carboxylic acid transmembrane transporter activity	0.0352436
GO:0048029	monosaccharide binding	0.01860236
GO:0050896	response to stimulus	0.03459633
GO:0051336	regulation of hydrolase activity	0.03048673
GO:0051345	positive regulation of hydrolase activity	0.01860236
GO:0070008	serine-type exopeptidase activity	0.01431698
GO:0071474	cellular hyperosmotic response	0.0352436
GO:1901565	organonitrogen compound catabolic process	0.03484065

Table S7. GO terms of proteins overexpressed only in Sp2 (*Serratia plymuthica*) treated with 0.4% NaCl vs Control.

GO Term	Description	P-value (Serratia)
GO:0000041	transition metal ion transport	0.03125707
GO:0000746	conjugation	0.004014615
GO:0001530	lipopolysaccharide binding	9.91E-05
GO:0003921	GMP synthase activity	0.02537498
GO:0003922	GMP synthase (glutamine-hydrolyzing) activity	0.02537498
GO:0004866	endopeptidase inhibitor activity	0.02537498
GO:0004867	serine-type endopeptidase inhibitor activity	0.02537498
GO:0006023	aminoglycan biosynthetic process	0.00984618
GO:0006024	glycosaminoglycan biosynthetic process	0.00984618
GO:0006857	oligopeptide transport	0.00051735
GO:0006865	amino acid transport	0.01483782
GO:0006869	lipid transport	0.000462792
GO:0007165	signal transduction	0.02601521
GO:0008252	nucleotidase activity	0.02537498
GO:0008745	N-acetylmuramoyl-L-alanine amidase activity	0.02537498
GO:0009252	peptidoglycan biosynthetic process	0.00984618
GO:0009597	detection of virus	0.004014615
GO:0009615	response to virus	0.004014615
GO:0010033	response to organic substance	0.0375981
GO:0010466	negative regulation of peptidase activity	0.02537498
GO:0010876	lipid localization	0.000857191
GO:0010951	negative regulation of endopeptidase activity	0.02537498
GO:0014070	response to organic cyclic compound	0.03125707
GO:0015267	channel activity	0.001730817
GO:0015288	porin activity	0.000186527
GO:0015688	Siderophore-dependent iron import into cell	0.01416171
GO:0015711	organic anion transport	0.000601766
GO:0015748	organophosphate ester transport	0.03125707
GO:0015807	L-amino acid transport	0.0027648
GO:0015891	siderophore transport	0.01416171
GO:0015914	phospholipid transport	0.03125707
GO:0015920	lipopolysaccharide transport	0.00051735
GO:0016021	integral component of membrane	0.03699752
GO:0019058	viral life cycle	0.02001884
GO:0022803	passive transmembrane transporter activity	0.001730817
GO:0022829	wide pore channel activity	0.000186527
GO:0030260	entry into host	0.00051735
GO:0030312	external encapsulating structure	0.000661262
GO:0030414	peptidase inhibitor activity	0.02537498
GO:0031224	intrinsic component of membrane	0.006235302
GO:0031230	intrinsic component of cell outer membrane	0.007176907
GO:0031241	periplasmic side of cell outer membrane	6.47E-05
GO:0031246	intrinsic component of periplasmic side of cell outer membrane	0.001576114
GO:0032978	protein insertion into membrane from inner side	0.02537498
GO:0034220	ion transmembrane transport	0.03775271
GO:0034613	cellular protein localization	0.02996041
GO:0042277	peptide binding	0.01483782
GO:0042930	enterobactin transport	0.01416171
GO:0042938	dipeptide transport	0.004014615
GO:0043163	cell envelope organization	2.26E-09
GO:0043165	Gram-negative-bacterium-type cell outer membrane assembly	2.26E-09
GO:0044038	cell wall macromolecule biosynthetic process	0.00984618
GO:0044091	membrane biogenesis	2.26E-09
GO:0044409	entry into host	0.00051735
GO:0045229	external encapsulating structure organization	1.36E-08
GO:0045861	negative regulation of proteolysis	0.02537498

GO Term	Description	P-value (Serratia)
GO:0046718	viral entry into host cell	0.00051735
GO:0046930	pore complex	0.00051735
GO:0051181	cofactor transport	0.03125707
GO:0051205	protein insertion into membrane	0.0027648
GO:0051701	interaction with host	0.02001884
GO:0051806	entry into host	0.00051735
GO:0051828	entry into other organism involved in symbiotic interaction	0.00051735
GO:0052547	regulation of peptidase activity	0.02537498
GO:0052548	regulation of endopeptidase activity	0.02537498
GO:0061024	membrane organization	3.56E-10
GO:0061134	peptidase regulator activity	0.02537498
GO:0061135	endopeptidase regulator activity	0.02537498
GO:0061783	peptidoglycan muralytic activity	0.00051735
GO:0070589	cellular component macromolecule biosynthetic process	0.00984618
GO:0070727	cellular macromolecule localization	0.02996041
GO:0071709	membrane assembly	2.26E-09
GO:0072657	protein localization to membrane	0.003660424
GO:0090150	establishment of protein localization to membrane	0.007259702
GO:0098552	side of membrane	0.001099312
GO:0098656	anion transmembrane transport	0.04108586
GO:0120009	intermembrane lipid transfer	0.03125707
GO:0120010	intermembrane phospholipid transfer	0.03125707
GO:1901264	carbohydrate derivative transport	0.000443946
GO:1901678	iron coordination entity transport	0.01416171
GO:1990063	Bam protein complex	0.0027648

Table S8. GO terms of proteins underexpressed both in Ra4 (*Rahnella aquatilis*) and in Sp2 (*Serratia plymuthica*) treated with 0.4% NaCl vs Control.

GO Term	Description	P-value (Rahnella)	P-value (Serratia)
GO:0000217	DNA secondary structure binding	0.01305778	0.004660561
GO:0000400	four-way junction DNA binding	0.01305778	0.004660561
GO:0004743	pyruvate kinase activity	0.01238103	0.04065939
GO:0004807	triose-phosphate isomerase activity	0.03622317	0.004660561
GO:0006066	alcohol metabolic process	0.002867256	0.003154162
GO:0006071	glycerol metabolic process	0.01238103	0.04065939
GO:0006115	ethanol biosynthetic process	0.03622317	0.004660561
GO:0008774	acetaldehyde dehydrogenase (acetylating) activity	0.03622317	0.004660561
GO:0019400	alditol metabolic process	0.01238103	0.04065939
GO:0019405	alditol catabolic process	0.01238103	0.02552491
GO:0019563	glycerol catabolic process	0.01238103	0.02552491
GO:0034309	primary alcohol biosynthetic process	0.03622317	0.004660561
GO:0040011	locomotion	0.02008507	0.000294434
GO:0046165	alcohol biosynthetic process	0.03622317	0.004660561
GO:0046166	glyceraldehyde-3-phosphate biosynthetic process	0.03622317	0.004660561
GO:0046184	aldehyde biosynthetic process	0.01238103	0.02552491
GO:1901615	organic hydroxy compound metabolic process	0.002781958	0.01572703
GO:1901617	organic hydroxy compound biosynthetic process	0.01238103	0.04065939

Table S9. GO terms of proteins underexpressed only in Ra4 (*Rahnella aquatilis*) treated with 0.4% NaCl vs Control.

GO Term	Description	P-value (Rahnella)
GO:0000096	sulfur amino acid metabolic process	0.004778599
GO:0000097	sulfur amino acid biosynthetic process	0.002867256
GO:0000966	RNA 5'-end processing	0.03622317
GO:0000967	rRNA 5'-end processing	0.03622317
GO:0003824	catalytic activity	0.004035336
GO:0004073	aspartate-semialdehyde dehydrogenase activity	0.01305778
GO:0004124	cysteine synthase activity	0.03622317
GO:0004356	glutamate-ammonia ligase activity	0.03622317
GO:0004375	glycine dehydrogenase (decarboxylating) activity	0.001475094
GO:0004470	malic enzyme activity	0.03622317
GO:0004816	asparagine-tRNA ligase activity	0.01305778
GO:0005622	intracellular	0.002424878
GO:0005737	cytoplasm	0.00930345
GO:0005829	cytosol	0.008273604
GO:0005975	carbohydrate metabolic process	0.01402159
GO:0006082	organic acid metabolic process	0.003733665
GO:0006090	pyruvate metabolic process	0.01341953
GO:0006096	glycolytic process	0.002644354
GO:0006108	malate metabolic process	0.03622317
GO:0006116	NADH oxidation	0.01305778
GO:0006163	purine nucleotide metabolic process	0.000936824
GO:0006164	purine nucleotide biosynthetic process	0.00040464
GO:0006165	nucleoside diphosphate phosphorylation	0.003468464
GO:0006421	asparaginyl-tRNA aminoacylation	0.01305778
GO:0006520	cellular amino acid metabolic process	0.00991025
GO:0006535	cysteine biosynthetic process from serine	0.03622317
GO:0006541	glutamine metabolic process	0.004380063
GO:0006542	glutamine biosynthetic process	0.03622317
GO:0006563	L-serine metabolic process	0.002867256
GO:0006725	cellular aromatic compound metabolic process	0.02721901
GO:0006732	coenzyme metabolic process	0.02120952
GO:0006733	oxidoreduction coenzyme metabolic process	0.01902246
GO:0006734	NADH metabolic process	0.01305778
GO:0006753	nucleoside phosphate metabolic process	0.01519071
GO:0006754	ATP biosynthetic process	0.000339656
GO:0006757	ATP generation from ADP	0.002644354
GO:0006793	phosphorus metabolic process	0.008471227
GO:0006796	phosphate-containing compound metabolic process	0.01151261
GO:0006935	chemotaxis	0.007988557
GO:0008152	metabolic process	0.03327529
GO:0008198	ferrous iron binding	0.01305778
GO:0008652	cellular amino acid biosynthetic process	0.006700054
GO:0009058	biosynthetic process	0.002741617
GO:0009069	serine family amino acid metabolic process	0.008564943
GO:0009070	serine family amino acid biosynthetic process	0.000644798
GO:0009086	methionine biosynthetic process	0.03644501
GO:0009088	threonine biosynthetic process	0.01305778
GO:0009089	lysine biosynthetic process via diaminopimelate	0.03644501
GO:0009090	homoserine biosynthetic process	0.03622317
GO:0009092	homoserine metabolic process	0.03622317
GO:0009108	coenzyme biosynthetic process	0.004646654
GO:0009117	nucleotide metabolic process	0.01519071
GO:0009123	nucleoside monophosphate metabolic process	0.00040464
GO:0009124	nucleoside monophosphate biosynthetic process	0.000133828
GO:0009126	purine nucleoside monophosphate metabolic process	0.00036557
GO:0009127	purine nucleoside monophosphate biosynthetic process	0.000241849
GO:0009132	nucleoside diphosphate metabolic process	0.001934503
GO:0009135	purine nucleoside diphosphate metabolic process	0.001471131
GO:0009141	nucleoside triphosphate metabolic process	0.004646654
GO:0009142	nucleoside triphosphate biosynthetic process	0.001010332

GO Term	Description	P-value (Rahnella)
GO:0009144	purine nucleoside triphosphate metabolic process	0.00254214
GO:0009145	purine nucleoside triphosphate biosynthetic process	0.000599766
GO:0009150	purine ribonucleotide metabolic process	0.001920768
GO:0009152	purine ribonucleotide biosynthetic process	0.001082809
GO:0009156	ribonucleoside monophosphate biosynthetic process	7.67E-05
GO:0009161	ribonucleoside monophosphate metabolic process	0.000124709
GO:0009165	nucleotide biosynthetic process	0.001818302
GO:0009166	nucleotide catabolic process	0.011015
GO:0009167	purine ribonucleoside monophosphate metabolic process	0.00036557
GO:0009168	purine ribonucleoside monophosphate biosynthetic process	0.000241849
GO:0009179	purine ribonucleoside diphosphate metabolic process	0.001471131
GO:0009185	ribonucleoside diphosphate metabolic process	0.001471131
GO:0009199	ribonucleoside triphosphate metabolic process	0.003829339
GO:0009201	ribonucleoside triphosphate biosynthetic process	0.001010332
GO:0009205	purine ribonucleoside triphosphate metabolic process	0.00254214
GO:0009206	purine ribonucleoside triphosphate biosynthetic process	0.000599766
GO:0009259	ribonucleotide metabolic process	0.002599612
GO:0009260	ribonucleotide biosynthetic process	0.001610841
GO:0009333	cysteine synthase complex	0.03622317
GO:0009593	detection of chemical stimulus	0.03622317
GO:0009730	detection of carbohydrate stimulus	0.03622317
GO:0009743	response to carbohydrate	0.03622317
GO:0015766	disaccharide transport	0.03622317
GO:0015768	maltose transport	0.03622317
GO:0015772	oligosaccharide transport	0.03622317
GO:0015774	polysaccharide transport	0.03622317
GO:0015821	methionine transport	0.01305778
GO:0016052	carbohydrate catabolic process	0.001157467
GO:0016053	organic acid biosynthetic process	0.001213802
GO:0016211	ammonia ligase activity	0.03622317
GO:0016310	phosphorylation	0.003257328
GO:0016477	cell migration	0.03622317
GO:0016491	oxidoreductase activity	0.02075898
GO:0016614	oxidoreductase activity, acting on CH-OH group of donors	0.01428636
GO:0016615	malate dehydrogenase activity	0.03622317
GO:0016620	oxidoreductase activity, acting on the aldehyde or oxo group of donors, NAD or NADP as acceptor	0.007988557
GO:0016638	oxidoreductase activity, acting on the CH-NH2 group of donors	0.001475094
GO:0016642	oxidoreductase activity, acting on the CH-NH2 group of donors, disulfide as acceptor	0.001475094
GO:0016740	transferase activity	0.02307052
GO:0016765	transferase activity, transferring alkyl or aryl (other than methyl) groups	0.004778599
GO:0016879	ligase activity, forming carbon-nitrogen bonds	0.03308006
GO:0016880	acid-ammonia (or amide) ligase activity	0.03622317
GO:0016903	oxidoreductase activity, acting on the aldehyde or oxo group of donors	0.0392069
GO:0017144	drug metabolic process	0.003784371
GO:0018130	heterocycle biosynthetic process	0.005195686
GO:0019344	cysteine biosynthetic process	0.03622317
GO:0019359	nicotinamide nucleotide biosynthetic process	0.002644354
GO:0019362	pyridine nucleotide metabolic process	0.01359257
GO:0019363	pyridine nucleotide biosynthetic process	0.002644354
GO:0019438	aromatic compound biosynthetic process	0.007948905
GO:0019439	aromatic compound catabolic process	0.04476282
GO:0019637	organophosphate metabolic process	0.01501704
GO:0019674	NAD metabolic process	0.01305778
GO:0019676	ammonia assimilation cycle	0.03622317
GO:0019693	ribose phosphate metabolic process	0.003845109
GO:0019740	nitrogen utilization	0.03622317
GO:0019751	polyol metabolic process	0.02270289
GO:0019752	carboxylic acid metabolic process	0.003733665
GO:0019898	extrinsic component of membrane	0.002059172
GO:0030170	pyridoxal phosphate binding	0.03631771

GO Term	Description	P-value (Rahnella)
GO:0031234	extrinsic component of cytoplasmic side of plasma membrane	0.01305778
GO:0033037	polysaccharide localization	0.03622317
GO:0033178	proton-transporting two-sector ATPase complex, catalytic domain	0.03644501
GO:0034022	3-(hydroxyamino)phenol mutase activity	0.03622317
GO:0034285	response to disaccharide	0.03622317
GO:0034286	response to maltose	0.03622317
GO:0034288	detection of disaccharide stimulus	0.03622317
GO:0034289	detection of maltose stimulus	0.03622317
GO:0034404	nucleobase-containing small molecule biosynthetic process	0.01338439
GO:0034471	ncRNA 5'-end processing	0.03622317
GO:0034654	nucleobase-containing compound biosynthetic process	0.009931168
GO:0034655	nucleobase-containing compound catabolic process	0.04476282
GO:0042330	taxis	0.007988557
GO:0042537	benzene-containing compound metabolic process	0.03622317
GO:0042866	pyruvate biosynthetic process	0.002644354
GO:0042940	D-amino acid transport	0.01305778
GO:0042956	maltodextrin transport	0.03622317
GO:0043190	ATP-binding cassette (ABC) transporter complex	0.004380063
GO:0043436	oxoacid metabolic process	0.003733665
GO:0043648	dicarboxylic acid metabolic process	0.001983787
GO:0044249	cellular biosynthetic process	0.004313104
GO:0044270	cellular nitrogen compound catabolic process	0.04476282
GO:0044272	sulfur compound biosynthetic process	0.04754571
GO:0044281	small molecule metabolic process	2.23E-05
GO:0044283	small molecule biosynthetic process	0.000119999
GO:0044424	obsolete intracellular part	0.002424878
GO:0044444	obsolete cytoplasmic part	0.01184802
GO:0045260	plasma membrane proton-transporting ATP synthase complex	0.03644501
GO:0045261	proton-transporting ATP synthase complex, catalytic core F(1)	0.03644501
GO:0045262	plasma membrane proton-transporting ATP synthase complex, catalytic core F(1)	0.03644501
GO:0045281	succinate dehydrogenase complex	0.03622317
GO:0045282	plasma membrane succinate dehydrogenase complex	0.03622317
GO:0045912	negative regulation of carbohydrate metabolic process	0.01305778
GO:0046031	ADP metabolic process	0.004482494
GO:0046034	ATP metabolic process	0.001632573
GO:0046164	alcohol catabolic process	0.02270289
GO:0046174	polyol catabolic process	0.02270289
GO:0046390	ribose phosphate biosynthetic process	0.001610841
GO:0046394	carboxylic acid biosynthetic process	0.001213802
GO:0046434	organophosphate catabolic process	0.01341953
GO:0046483	heterocycle metabolic process	0.04421
GO:0046496	nicotinamide nucleotide metabolic process	0.01359257
GO:0046658	anchored component of plasma membrane	0.01305778
GO:0046939	nucleotide phosphorylation	0.005714416
GO:0048030	disaccharide binding	0.03622317
GO:0048473	D-methionine transport	0.01305778
GO:0050486	intramolecular transferase activity, transferring hydroxy groups	0.03622317
GO:0051188	cofactor biosynthetic process	0.01106151
GO:0051606	detection of stimulus	0.03622317
GO:0055052	ATP-binding cassette (ABC) transporter complex, substrate-binding subunit-containing	0.000753188
GO:0055086	nucleobase-containing small molecule metabolic process	0.01359174
GO:0055114	oxidation-reduction process	0.04831034
GO:0060326	cell chemotaxis	0.03622317
GO:0070279	vitamin B6 binding	0.03631771
GO:0070403	NAD+ binding	0.01305778
GO:0070492	oligosaccharide binding	0.03622317
GO:0072330	monocarboxylic acid biosynthetic process	0.03762314
GO:0072521	purine-containing compound metabolic process	0.002216701
GO:0072522	purine-containing compound biosynthetic process	0.000501629
GO:0072524	pyridine-containing compound metabolic process	0.006617782
GO:0072525	pyridine-containing compound biosynthetic process	0.001102393

GO Term	Description	P-value (Rahnella)
GO:0080146	L-cysteine desulfhydrase activity	0.03622317
GO:0090407	organophosphate biosynthetic process	0.002707148
GO:0098533	ATPase dependent transmembrane transport complex	0.004380063
GO:0098797	plasma membrane protein complex	0.002644354
GO:1901135	carbohydrate derivative metabolic process	0.04819767
GO:1901137	carbohydrate derivative biosynthetic process	0.004888293
GO:1901292	nucleoside phosphate catabolic process	0.011015
GO:1901293	nucleoside phosphate biosynthetic process	0.001818302
GO:1901360	organic cyclic compound metabolic process	0.01621869
GO:1901362	organic cyclic compound biosynthetic process	0.003742089
GO:1901566	organonitrogen compound biosynthetic process	0.000889639
GO:1901576	organic substance biosynthetic process	0.00254786
GO:1901605	alpha-amino acid metabolic process	0.008698654
GO:1901607	alpha-amino acid biosynthetic process	0.007976329
GO:1901616	organic hydroxy compound catabolic process	0.02270289
GO:1901982	maltose binding	0.03622317
GO:1902495	transmembrane transporter complex	0.0392069
GO:1904949	ATPase complex	0.02862085
GO:1990351	transporter complex	0.0392069

Table S10. GO terms of proteins underexpressed only in Sp2 (*Serratia plymuthica*) treated with 0.4% NaCl vs Control.

GO Term	Description	P-value (Serratia)
GO:0000027	ribosomal large subunit assembly	0.01413104
GO:0000028	ribosomal small subunit assembly	0.000351472
GO:0001539	cilium or flagellum-dependent cell motility	0.009156847
GO:0003676	nucleic acid binding	0.007824212
GO:0003723	RNA binding	0.04663955
GO:0003735	structural constituent of ribosome	0.009758061
GO:0004022	alcohol dehydrogenase (NAD+) activity	0.000312574
GO:0004520	endodeoxyribonuclease activity	0.04065939
GO:0005198	structural molecule activity	0.009758061
GO:0005576	extracellular region	0.001255743
GO:0005840	ribosome	0.01065786
GO:0006067	ethanol metabolic process	0.01335653
GO:0006310	DNA recombination	0.04065939
GO:0006417	regulation of translation	0.03205971
GO:0006566	threonine metabolic process	0.001996164
GO:0006567	threonine catabolic process	0.000617077
GO:0006928	movement of cell or subcellular component	0.000585109
GO:0006996	organelle organization	0.000868432
GO:0007154	cell communication	0.02406593
GO:0008861	formate C-acetyltransferase activity	9.81E-05
GO:0009063	cellular amino acid catabolic process	0.03619223
GO:0009066	aspartate family amino acid metabolic process	0.01596951
GO:0009068	aspartate family amino acid catabolic process	0.001169839
GO:0009288	bacterial-type flagellum	3.00E-09
GO:0009314	response to radiation	0.0433478
GO:0009409	response to cold	0.03010927
GO:0009424	bacterial-type flagellum hook	0.001188158
GO:0009425	bacterial-type flagellum basal body	0.01335653
GO:0009432	SOS response	0.02552491
GO:0009893	positive regulation of metabolic process	0.0215086
GO:0010212	response to ionizing radiation	0.03444911
GO:0010604	positive regulation of macromolecule metabolic process	0.0215086
GO:0010608	posttranscriptional regulation of gene expression	0.04554022
GO:0010628	positive regulation of gene expression	0.04929504
GO:0015935	small ribosomal subunit	0.003133877
GO:0016043	cellular component organization	0.01725526
GO:0016407	acetyltransferase activity	0.00668461
GO:0016408	C-acyltransferase activity	0.001996164
GO:0016453	C-acetyltransferase activity	0.000617077
GO:0017148	negative regulation of translation	0.02822709
GO:0019843	rRNA binding	0.002618538
GO:0022607	cellular component assembly	0.00978186
GO:0022613	ribonucleoprotein complex biogenesis	0.000930684
GO:0022618	ribonucleoprotein complex assembly	0.000229534
GO:0022626	cytosolic ribosome	0.01065786
GO:0022627	cytosolic small ribosomal subunit	0.003133877
GO:0031323	regulation of cellular metabolic process	0.04814551
GO:0031325	positive regulation of cellular metabolic process	0.01596951
GO:0032268	regulation of cellular protein metabolic process	0.006029083
GO:0032269	negative regulation of cellular protein metabolic process	0.0433478
GO:0032270	positive regulation of cellular protein metabolic process	0.009156847
GO:0034248	regulation of cellular amide metabolic process	0.04554022
GO:0034249	negative regulation of cellular amide metabolic process	0.02822709
GO:0034308	primary alcohol metabolic process	0.02552491
GO:0034622	cellular protein-containing complex assembly	0.000359931
GO:0042254	ribosome biogenesis	0.000718876
GO:0042255	ribosome assembly	0.000167364

GO Term	Description	P-value (Serratia)
GO:0042273	ribosomal large subunit biogenesis	0.01413104
GO:0042274	ribosomal small subunit biogenesis	0.000752232
GO:0042995	cell projection	3.00E-09
GO:0043226	organelle	8.01E-06
GO:0043228	non-membrane-bounded organelle	9.70E-06
GO:0043229	intracellular organelle	0.02928948
GO:0043232	intracellular non-membrane-bounded organelle	0.02367969
GO:0043933	protein-containing complex subunit organization	0.003362565
GO:0044085	cellular component biogenesis	0.0389506
GO:0044391	ribosomal subunit	0.01065786
GO:0044422	obsolete organelle part	0.000452334
GO:0044445	obsolete cytosolic part	0.02481009
GO:0044446	obsolete intracellular organelle part	0.01375375
GO:0044461	obsolete bacterial-type flagellum part	9.81E-05
GO:0044463	obsolete cell projection part	9.81E-05
GO:0048518	positive regulation of biological process	0.01226333
GO:0048522	positive regulation of cellular process	0.006543907
GO:0048870	cell motility	0.000585109
GO:0051173	positive regulation of nitrogen compound metabolic process	0.01150958
GO:0051246	regulation of protein metabolic process	0.006029083
GO:0051247	positive regulation of protein metabolic process	0.009156847
GO:0051248	negative regulation of protein metabolic process	0.0433478
GO:0051674	localization of cell	0.000585109
GO:0065003	protein-containing complex assembly	0.002788147
GO:0070925	organelle assembly	0.000149083
GO:0071826	ribonucleoprotein complex subunit organization	0.000229534
GO:0071973	bacterial-type flagellum-dependent cell motility	0.009156847
GO:0071978	bacterial-type flagellum-dependent swarming motility	0.000279027
GO:0097588	archaeal or bacterial-type flagellum-dependent cell motility	0.009156847
GO:1901606	alpha-amino acid catabolic process	0.03010927
GO:1990904	ribonucleoprotein complex	0.01492792
